# The Role of Yes‐Associated Protein in Inflammatory Diseases and Cancer

**DOI:** 10.1002/mco2.70128

**Published:** 2025-03-10

**Authors:** Bing Zhong, Jintao Du, Feng Liu, Silu Sun

**Affiliations:** ^1^ Department of Otolaryngology‐Head and Neck Surgery West China Hospital Sichuan University Chengdu Sichuan China; ^2^ State Key Laboratory of Oral Diseases National Clinical Research Center for Oral Diseases Chinese Academy of Medical Sciences Research Unit of Oral Carcinogenesis and Management West China Hospital of Stomatology Sichuan University Chengdu Sichuan China

**Keywords:** cancer, Hippo pathway, inflammatory diseases, Yes‐associated protein

## Abstract

Yes‐associated protein (YAP) plays a central role in the Hippo pathway, primarily governing cell proliferation, differentiation, and apoptosis. Its significance extends to tumorigenesis and inflammatory conditions, impacting disease initiation and progression. Given the increasing relevance of YAP in inflammatory disorders and cancer, this study aims to elucidate its pathological regulatory functions in these contexts. Specifically, we aim to investigate the involvement and molecular mechanisms of YAP in various inflammatory diseases and cancers. We particularly focus on how YAP activation, whether through Hippo‐dependent or independent pathways, triggers the release of inflammation and inflammatory mediators in respiratory, cardiovascular, and digestive inflammatory conditions. In cancer, YAP not only promotes tumor cell proliferation and differentiation but also modulates the tumor immune microenvironment, thereby fostering tumor metastasis and progression. Additionally, we provide an overview of current YAP‐targeted therapies. By emphasizing YAP's role in inflammatory diseases and cancer, this study aims to enhance our understanding of the protein's pivotal involvement in disease processes, elucidate the intricate pathological mechanisms of related diseases, and contribute to future drug development strategies targeting YAP.

## Graphical abstract

The activation and malfunction of Yes‐associated protein (YAP) play a role in enhancing the release of inflammation and inflammatory mediators in respiratory, cardiovascular, and digestive inflammatory disorders. In cancer, YAP drives the growth and differentiation of tumor cells in head and neck, digestive system, and urinary system cancers, influencing the tumor immune microenvironment and facilitating tumor metastasis and advancement.

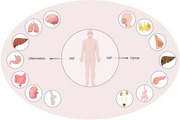

## 1 Introduction

**FIGURE 1:**
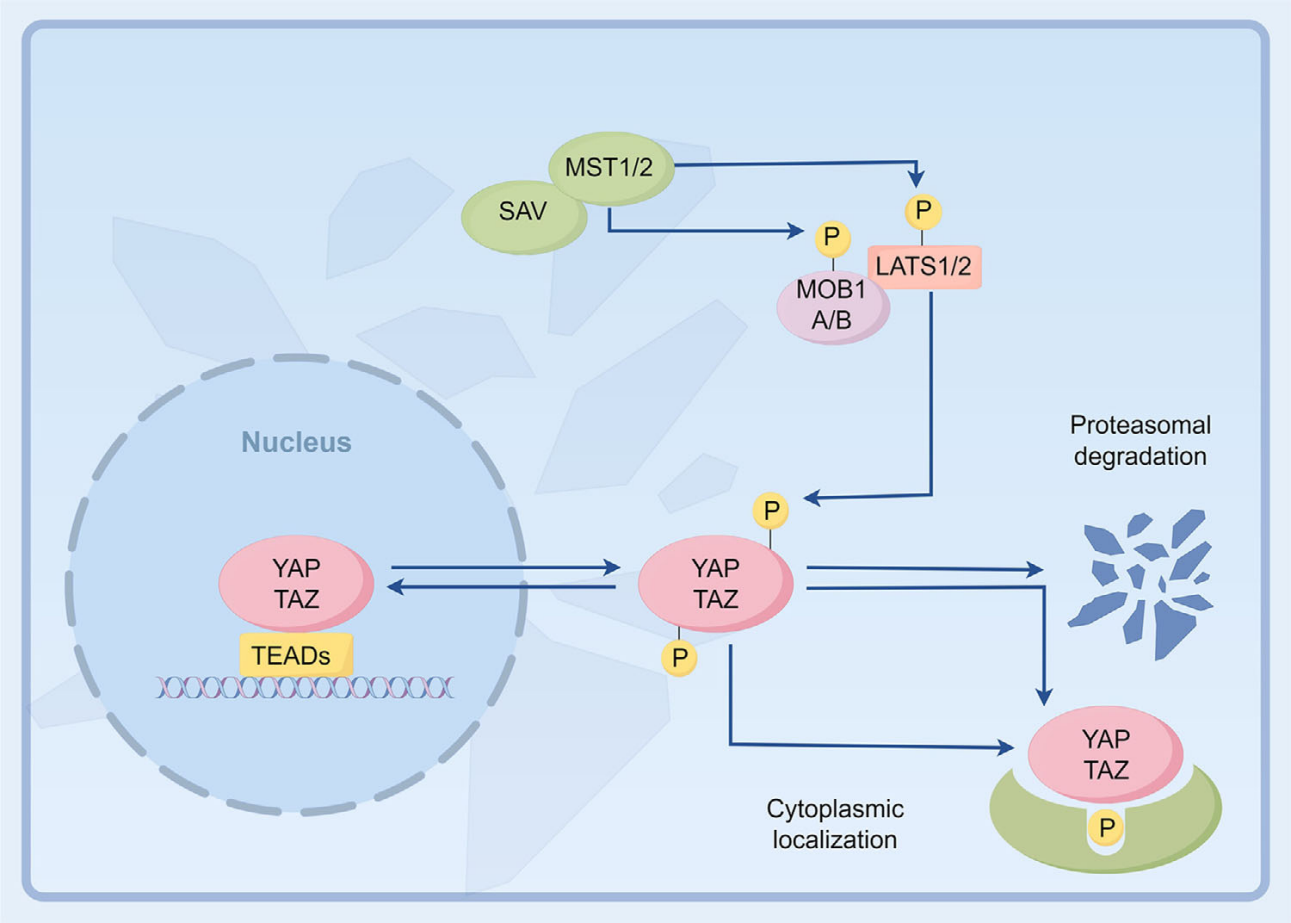
Schematic diagram of the mechanism of hippo pathway. When the Hippo pathway is activated, it triggers the phosphorylation of MST1/2 and its scaffold protein SAV1. This phosphorylation, in turn, promotes the phosphorylation of LATS1/2 and its scaffold protein MOB1A/B. These phosphorylation events ultimately lead to the phosphorylation of YAP. Consequently, YAP either undergoes degradation or binds to 14‐3‐3 proteins, causing its retention in the cytoplasm. On the other hand, when the Hippo pathway is deactivated, YAP translocates to the nucleus and interacts with TEAD1–4. This interaction facilitates downstream gene transcription, thereby enabling YAP to exert its biological activities. YAP, Yes‐associated protein; TAZ, transcriptional coactivator with PDZ‐binding motif; SAV, protein salvador homolog 1; MST1/2, mammalian sterile 20‐like protein kinase 1/2; MOB1 A/B, MOB kinase activator 1A; LATS1/2, large tumor suppressor homolog 1/2; TEADs, TEA domain transcription factors. The figure is created by Figdraw.

The Hippo pathway is a critical regulatory network that governs cell proliferation, differentiation, tissue development, and immune homeostasis. At its core, the Yes‐associated protein (YAP) plays a central role in this pathway [1, 2, 3, 4]. In an active state of the Hippo pathway, LATS1/2 and their scaffold MOB1A/B are phosphorylated by MST1/2 and their scaffold protein SAV1. This phosphorylation event subsequently leads to the phosphorylation of YAP, inhibiting its activation and translocation, resulting in its sequestration in the cytoplasm and eventual degradation via the proteasome pathway. Conversely, when the Hippo pathway is inactive, the phosphorylation of MST1/2 and LATS1/2 is suppressed, leading to reduced phosphorylation of YAP. Consequently, YAP translocates to the nucleus, where it interacts with TEAD 1–4 transcription factors, initiating downstream gene transcription [5, 6, 7, 8, 9] (Figure 1). As a pivotal gene in the Hippo pathway, YAP plays a critical role in the initiation and progression of various inflammatory conditions in the body [10, 11, 12]. YAP serves as a crucial regulator in macrophage inflammation and the sensing of stiffness [13]. The activation of the ataxia telangiectasia mutated (ATM)–YAP1–pro‐IL‐18 pathway in epithelial cells, induced by telomere dysfunction, serves as a significant trigger for tissue inflammation [14]. Moreover, the YAP–TEAD transcriptional activity enhances autophagy flux and lysosomal acidification, thereby strengthening defenses against intracellular Staphylococcus aureus through an innate immune response [15]. YAP also maintains the stability of NOD‐like receptor protein 3 (NLRP3) by preventing its binding to the E3 ligase β‐TrCP1, thereby promoting inflammation [16]. Furthermore, YAP plays a role in the body's immune response to viral infections. The upregulation of ATP6V0d2 due to impaired serine metabolism leads to YAP lysosomal degradation, alleviating YAP‐mediated inhibition of the TBK1–interferon regulatory factor 3 (IRF3) axis and enhancing interferon‐β (IFN‐β)‐mediated antiviral innate immunity [11]. Additionally, lysophosphatidic acid induces Epstein–Barr virus lytic infection in epithelial cells through a YAP/TAZ‐dependent mechanism [17]. YAP has garnered significant attention as a key player in tumor development and metastasis. It has been implicated in the progression and inflammatory responses of malignant tumors such as liver cancer [18], lung cancer [19], and breast cancer [20].

The activated state of YAP exerts a pivotal role in propelling the proliferation and metastasis of cancer cells. Moreover, it regulates the tumor immune microenvironment and inflammatory milieu by triggering inflammatory pathways. Nevertheless, the expression patterns and molecular pathways underlying YAP activation, whether they are Hippo pathway dependent or independent, vary significantly among different cell types in diverse diseases, and there is currently a dearth of comprehensive overviews. In light of the intricate role of YAP in disease pathogenesis, this study comprehensively summarizes the impacts of Hippo‐pathologically dependent and independent activation of YAP on various cell types in the context of inflammation and cancer, and delves into its correlations with these diseases. This encompasses an exploration of whether YAP activation is Hippo pathway dependent or independent in inflammatory diseases, such as chronic rhinosinusitis (CRS), asthma, cystic fibrosis (CF) and pulmonary fibrosis, chronic obstructive pulmonary disease (COPD), viral infection in airway, cardiovascular inflammatory diseases, liver inflammatory diseases, colitis, nerve damage and cerebral hemorrhage, kidney injury, osteoarthritis, and diabetes. It also investigates YAP's role in different cancers, including head and neck cancer, nasopharyngeal carcinoma, thyroid cancer, malignant peripheral nerve sheath tumors, melanoma, breast cancer, gastric cancer, liver cancer, cholangiocarcinoma, pancreatic cancer, colorectal cancer, lung cancer, renal cancer, bladder cancer, and prostate cancer (Table 1). Additionally, the study summarizes the treatment strategies targeting different aspects of YAP in these diseases.

**TABLE 1:** Expression of YAP in respiratory diseases.

Diseases	Cell types	Activated YAP (Yes‐associated protein)
YAP in inflammatory diseases		
Chronic rhinosinusitis	Epithelial cells	↑
Asthma	Epithelial cells	↑
	Fibroblasts	↑
	Smooth muscle cells	↑
	Th17 cells	↑
Cystic fibrosis	Epithelial cells	↑
	Fibroblasts	↑
Pulmonary fibrosis	Epithelial cells	↑
	Fibroblasts	↑
Chronic obstructive pulmonary disease	Fibroblasts	↑
Influenza A	Human alveolar basal epithelial cells	↑
Pulmonary hypertension	Smooth muscle cells	↑
Atherosclerosis	Endothelial cells	↑
Myocardial infarction	Cardiomyocyte	↑(Promote cell proliferation)
Cardiac reperfusion injury	Cardiomyocyte	↑(Damage suppression)
Liver ischemia–reperfusion injury	Epithelial cells	↑(Damage suppression)
	Liver cells	↑(Damage suppression)
	Macrophage	↓(Promote inflammation)
Hepatitis	Liver cells	↑
	Mesenchymal stem cells	↑
	Macrophage	↑
Colitis	Epithelial cells	↑
	Fibroblasts	↑
	Smooth muscle cells	↑
	Macrophage	↑
Nerve damage and cerebral hemorrhage	Neurocyte	↓(Promote inflammation)
	Astrocytes	↑(Nerve damage)
	Vascular endothelial cells	↑(Angiogenesis)
Kidney injury	Renal tubular cells	↑
Osteoarthritis	Synovial cells	↑
	Chondrocyte	↑
Sepsis	Endothelial cells	↑(Suppress inflammation)
	Macrophage	↑(Promote endothelial cell permeability)
Diabetes	Endothelial cells	↑(Promote Atherosclerosis)
	Renal podocyte	↓(Promote diabetic nephropathy)
	Mesangial cells	↑(Promote diabetic nephropathy)
	Renal tubular cells	↑(Promote diabetic nephropathy)
YAP in cancers		
Nasopharyngeal carcinoma	Cancer cells	↑
Thyroid cancer	Cancer cells	↑
Malignant peripheral nerve sheath tumors	Schwann cells	↑
Melanoma	Cancer cells	↑
	Fibroblasts	↑
Breast cancer	Cancer cells	↑
Gastric cancer	Cancer cells	↑
Liver cancer	Cancer cells	↑
	Myeloid cells	↑
Cholangiocarcinoma	Cancer cells	↑
Pancreatic cancer	Cancer cells	↑
Colorectal cancer	Cancer cells	↑
Lung cancer	Cancer cells	↑
Renal cancer	Cancer cells	↑
Bladder cancer	Cancer cells	↑
Prostate cancer	Cancer cells	↑

## 2 YAP in Inflammatory Diseases

In the context of inflammatory diseases, the coexistence of aberrant cell proliferation and the initiation of inflammatory pathways is a common occurrence. Studies have confirmed that the activation of YAP can stimulate cell proliferation and differentiation within the inflammatory environment, regulate the activation of inflammatory pathways, modulate the secretion of inflammatory factors, and influence the immune landscape of the body. In different bodily systems such as the respiratory, digestive, circulatory, urinary, and skeletal systems, the activation of YAP emerges as a key player, playing a substantial role in disease progression and immune responses.

### 2.1 Mechanism of YAP Activation in Inflammation

The activation mechanism of YAP in the context of inflammation is indeed highly complex. Understanding the role that YAP plays in inflammatory diseases requires considering multiple perspectives. Research has highlighted that the stiffness of the extracellular matrix plays a crucial role in regulating YAP activity. A softer extracellular matrix environment can decrease YAP's nuclear localization, leading to its retention in the cytoplasm. Conversely, a stiffer extracellular matrix can enhance YAP's nuclear localization and activation. This interplay between the extracellular matrix stiffness and YAP activity adds another layer of complexity to the regulatory mechanisms involved in inflammatory responses [13, 21, 22]. Additionally, in the context of inflammation, a variety of cytokines and growth factors have the ability to activate YAP [23, 24]. For example, tumor necrosis factor‐alpha (TNF‐α) enhances YAP activity through the activation of the nuclear factor kappa B (NF‐κB) pathway [25, 26, 27], while both interleukin (IL)‐6 and IL‐13 have the capacity to facilitate the nuclear translocation of YAP [28, 29]. Both hypoxia and oxidative stress can reduce YAP phosphorylation and activate YAP by inhibiting the upstream kinase of the Hippo pathway [30, 31, 32, 33].

### 2.2 Chronic Rhinosinusitis

CRS encompasses subtypes with nasal polyps (CRSwNP) and without nasal polyps [34, 35]. The primary pathological characteristics include nasal mucosa edema and infiltration of inflammatory cells [36, 37]. YAP, a pivotal factor in CRS [38], is predominantly investigated for its involvement in the proliferation and differentiation of nasal epithelial cells [39] (Figure S1A). Multiple stimuli activate YAP, leading to the pathological condition of the nasal mucosa epithelium. Specifically, IL‐17A is notably elevated in the nasal epithelium of CRSwNP, negatively affecting active cilia morphogenesis by activating YAP [40]. Stimulation with lipopolysaccharide (LPS) and Poly(I:C) also boosts YAP expression, promoting nasal epithelial cell proliferation and remodeling, which can ultimately contribute to the formation of CRSwNP [41]. Furthermore, IL‐13 induces YAP overexpression, facilitating the proliferation and differentiation of basal cells and goblet cell proliferation in the nasal mucosa epithelium [28, 42]. Increased YAP levels play a role in CRSwNP epithelial barrier damage via the transforming growth factor‐β1 (TGF‐β1) signaling pathway, leading to the downregulation of TGF‐β1, zonula occludens‐1, and E‐cadherin protein levels [43]. This elevation also promotes goblet cell and basal cell proliferation [41, 44] and may influence the regulation of epithelial–mesenchymal transition (EMT) [45]. Inhibiting YAP activity with verteporfin (VP) suppresses nasal epithelial cell proliferation in CRSwNP and decreases the expression of epithelial cytokines [28, 42, 46]. However, prior studies on CRS have primarily focused on the role of YAP without delving into the involvement of the Hippo pathway or other signaling cascades.

### 2.3 Asthma

In asthma, airway obstruction arises from the activation of various immune cells, including dendritic cells, eosinophils, neutrophils, and mast cells [47, 48]. This activation triggers the release of inflammatory factors, leading to airway hyperreactivity [49, 50, 51]. Dysregulation of the YAP/TAZ signaling pathway is linked to the onset and progression of COPD and asthma [52, 53] (Figure S1B). The FERM domain containing 6 (FRMD6)/Hippo/YAP1 pathway may be involved in asthma pathogenesis [54]. Thrombin induces the doublecortin like kinase 1/Ras homolog gene family, member A (RhoA) signaling pathway, activating YAP and forming YAP/p65, which enhances IL‐8/chemokine (C–X–C motif) ligand 8 (CXCL8) expression through NF‐κB binding, contributing to asthma development [55]. However, some studies have reported a decrease in YAP1 expression in asthma [56], possibly due to variations in YAP expression and regulation across different tissues and cell types.

#### 2.3.1 Bronchial Epithelial Cells

The bronchial epithelium plays a crucial role in the pathogenesis and progression of asthma. When the epithelial barrier is compromised and inflammatory mediators are released, it leads to airway remodeling and increased airway hyperreactivity [57, 58, 59, 60]. Unlike the canonical Hippo pathway, YAP activation in airway epithelial cells can be influenced by alternative factors. The adhesion molecule catenin alpha‐like 1 promotes mucous secretion in airway epithelial cells via the YAP–Rho‐associated coiled‐coil forming protein kinase (ROCK)2 pathway [61]. Poly(I:C) triggers airway goblet cell hyperplasia, mucous secretion, and airway hyperreactivity in asthmatic mice through the YAP/Forkhead box protein M1 (FOXM1) pathway [62]. Furthermore, abnormal activation of the ERBB–YAP axis also plays a role in airway epithelial remodeling [63]. Additionally, YAP activation can boost the mammalian target of rapamycin/phosphorylated ribosomal protein S6 (p‐S6) signaling pathway, promoting the proliferation, migration, and appropriate differentiation of airway epithelial cells [64]. Tripterine inhibits YAP expression, consequently decreasing the levels of IL‐6, IL‐8, IL‐1β, and mucin 5AC in bronchial epithelial cells induced by LPS [65].

#### 2.3.2 Fibroblasts

In response to inflammatory mediators, fibroblasts can stimulate abnormal synthesis and deposition of extracellular matrix, leading to airway remodeling. Moreover, they release a range of cytokines to drive inflammation [59, 66, 67, 68]. FOXL1 can regulate TAZ/YAP to boost the proliferation of airway fibroblasts [69]. The inhibition of YAP by prostaglandin E(2) reduces fibroblast output, thereby alleviating the pathological progression of asthma [70].

#### 2.3.3 Smooth Muscle Cells

Smooth muscle cells exacerbate airway vasoconstriction and hyperresponsiveness in asthma in response to inflammatory mediators and cytokines [71, 72, 73, 74]. The elevated presence of YAP protein in the bronchial smooth muscle of asthmatic mice contributes to the progression of asthma [75]. In the Hippo pathway, research has shown that Fibulin‐5, in conjunction with β1 integrin, modulates the HIPPO–YAP/TAZ pathway, thus controlling the proliferation and migration of airway smooth muscle cells [76]. Direct stimulation of YAP activation has been increasingly reported. Sphingosine‐1‐phosphate (S1P) binding to S1PR (2/3) triggers the proliferation, migration, and contraction of airway smooth muscle cells in asthma by modulating the ROCK/YAP/FOXM1 axis [77]. Thrombin induces actin stress fiber polymerization via the protease‐activated receptor 1/RhoA/ROCK/myosin light chain 2 axis, activates YAP, and then interacts with mothers against decapentaplegic (SMAD)2 in the nucleus to trigger downstream target genes, ultimately fostering airway smooth muscle remodeling in asthma [78]. The thyroid receptor interactor protein 6 activates YAP, enhancing the proliferation and migration of airway smooth muscle cells during fetal development and contributing to the progression of asthma [79]. The miR‐15b‐5p/YAP1 axis potentially influences asthma development by modulating airway smooth muscle cell growth, migration, inflammatory response, and extracellular matrix deposition [80]. It is important to note that angiomotin‐like 2 and cysteine and glycine‐rich protein 2 (Csrp2) both impede asthma progression by suppressing YAP1 activation and restraining airway smooth muscle cell proliferation [81, 82]. Furthermore, lipoxin A4 obstructs Smad/YAP signaling, thereby inhibiting the proliferation and migration of airway smooth muscle cells in rats and averting the onset of asthma [83].

#### 2.3.4 T Helper 17 Cells

T helper cell (Th)17 cells play a pivotal role in fostering asthma inflammation by secreting IL‐17, which instigates airway inflammation and hyperreactivity. Additionally, an imbalance between Th17 cells and Treg cells further exacerbates the advancement of asthma [84, 85, 86]. Moreover, the activation of the YAP/hypoxia inducible factor‐1α (HIF‐1α)/miR‐182/early growth response protein 2 axis exacerbates lipid metabolism dysfunction and worsens asthma by stimulating the differentiation of Th17 cells [87].

### 2.4 CF and Pulmonary Fibrosis

CF is a common genetic disorder caused by a mutation in the cystic fibrosis transmembrane conductance regulator gene. This gene regulates the flow of ions across cell membranes, which is crucial for maintaining the appropriate consistency of bodily mucus. When dysfunctional, it can cause increased mucus viscosity, leading to lung infections [88, 89, 90, 91]. YAP1, an influential factor in the epithelial mesenchymal transition within CF, plays a significant role [92]. Apart from the Hippo pathway, the dehydration of airway surface fluids in CF stimulates the activation of the β1‐integrin/YAP1 signaling pathway, compromising the integrity of the airway epithelial barrier [93].

Pulmonary fibrosis is an interstitial lung disease characterized by fibroblast proliferation and large amount of extracellular matrix deposition [94, 95, 96, 97]. Previous research has demonstrated the existence of the IL‐6‐Rous sarcoma oncogene (Src) family kinases–YAP grade pathway outside the Hippo pathway, which enhances airway epithelial fluidification and consequently contributes to pulmonary fibrosis [24]. TGF‐β triggers a swift rise in adenosine 5'‐monophosphate‐activated protein kinase (AMPK)‐related protein kinase 1 (NUAK1) in fibroblasts, fostering profibrotic YAP and TGF‐β/SMAD signaling pathways, ultimately culminating in idiopathic pulmonary fibrosis [98]. The activation of DRD1 selectively inhibits YAP/TAZ function in mesenchymal cells and stiffens the extracellular matrix, thereby promoting idiopathic pulmonary fibrosis [99]. YAP/TAZ further enhances idiopathic pulmonary fibrosis by impeding prostaglandin receptor activation and encouraging the transition of fibroblasts into myofibroblasts [100].

### 2.5 Chronic Obstructive Pulmonary Disease

COPD presents as inflammation predominantly infiltrated by macrophages and neutrophils in the peripheral airway and lung parenchyma. This inflammatory reaction leads to structural changes in the airway, including airway epithelial metaplasia, mucous hyperplasia, peribronchiolar fibrosis, and destruction of the alveolar wall, ultimately culminating in emphysema [101, 102]. In mice with emphysema, the administration of human umbilical cord‐derived mesenchymal stem cells has been shown to alleviate the pathological condition by boosting the phosphorylation of YAP in type II alveolar epithelial cells [103]. Furthermore, exposure to cadmium triggers vimentin phosphorylation in lung fibroblasts and promotes the formation of YAP1 and Smad2/3 complexes, thus playing a role in the advancement of COPD [104] (Figure S1C).

### 2.6 Viral Infection in Airway

Respiratory viral infections, including influenza A, influenza B, and coronavirus disease 2019 (COVID‐19), can compromise the integrity of the respiratory epithelial barrier. This can lead to cellular damage, inflammation, heightened mucus and sputum production, and activation of the immune system, subsequently triggering immune responses [105, 106, 107, 108, 109, 110]. There is a suggestion that the Hippo–YAP signaling pathway may be involved in the progression of SARS‐CoV‐2 infection [111, 112]. However, this hypothesis lacks concrete experimental confirmation. It suggests that the physical interaction between the nonstructural protein 1 of the influenza A virus and the YAP/TAZ C‐terminal domain could activate YAP and TAZ in human alveolar basal epithelial cells. This interaction may also suppress Toll‐like receptor 3 (TLR3) expression, potentially hindering antiviral innate immune signaling [113] (Figure S1C).

### 2.7 Cardiovascular Inflammatory Diseases

Cardiovascular disease primarily stems from dysfunction in the heart and blood vessels, where abnormal angiogenesis plays a crucial role in its pathological processes. In angiogenesis models, the lack of endothelial FAT tumor suppressor 1 (FAT1) leads to heightened levels of YAP/TAZ proteins and the activation of characteristic YAP/TAZ target genes. This scenario promotes enhanced proliferation of endothelial cells [114]. YAP recruits the epigenetic inhibitory factor histone deacetylase‐4 through CAT elements to repress the expression of the phase‐retarding gene G protein‐coupled receptor 132 (Gpr132). This mechanism ultimately boosts the proliferation of vascular smooth muscle cells [115].

#### 2.7.1 Pulmonary Hypertension

**FIGURE 2:**
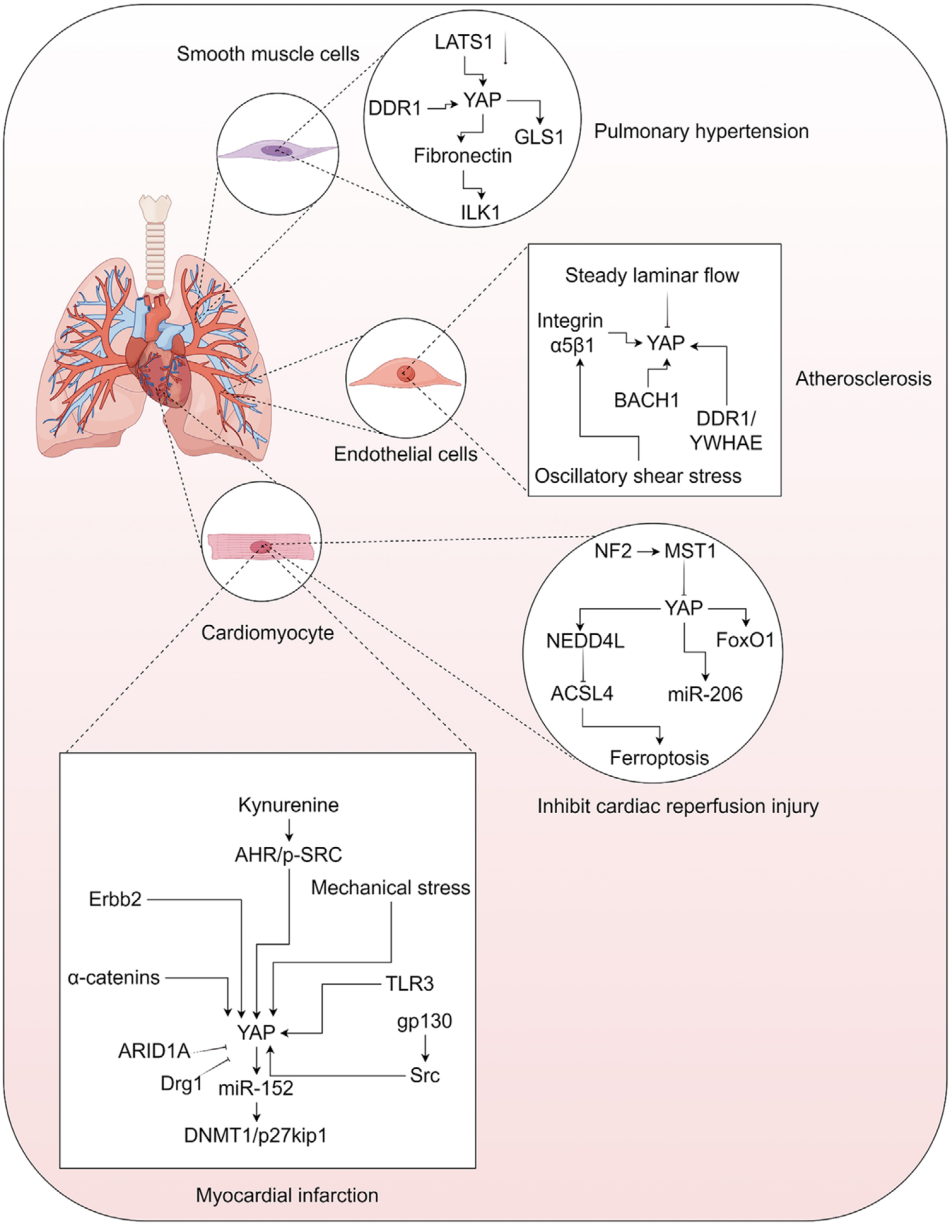
Molecular mechanism of YAP in cardiovascular disease. Mechanism of YAP in smooth muscle cells of pulmonary hypertension; mechanism of YAP in endothelial cells of atherosclerosis; YAP mechanism of cardiomyocyte in myocardial infarction and cardiac reperfusion injury. ACSL4, acyl‐CoA synthetase long chain family member 4; AHR, aryl hydrocarbon receptor; ARID1A, AT‐rich interaction domain 1A protein; BACH1, BTB domain and CNC homolog 1; DDR1, discoidin domain receptor family, member 1; DNMT1, DNA methyltransferase 1; Drg1, developmentally regulated GTP binding protein 1; ERbb2, human epidermal growth factor receptor 2; FoxO1, Forkhead box protein O1; GLS1, glutaminase kidney isoform, mitochondrial; gp130, glycoprotein 130; ILK1, integrin linked kinase 1; LATS1/2, large tumor suppressor homolog 1/2; MST1, mammalian sterile 20‐like protein kinase 1; NEDD4L, neural precursor cell expressed, developmentally downregulated 4‐like; NF2, Neurofibromatosis Type 2; p27kip1, cyclin‐dependent kinase inhibitor 1B; TLR3, Toll‐like receptor 3; YAP, Yes‐associated protein; SRC, proto‐oncogene tyrosine‐protein kinase Src; SRC, Src homology 2 domain containing transforming protein; YWHAE, tyrosine 3‐monooxygenase/tryptophan 5‐monooxygenase activation protein, epsilon polypeptide. The figure is created by Figdraw.

Pulmonary hypertension refers to changes in the structure and function of pulmonary blood vessels caused by various factors, resulting in increased pressure in the pulmonary artery [116, 117, 118]. The mechanical transduction of YAP and TAZ plays a crucial role in directing stem cell behavior and regeneration, influencing conditions like atherosclerosis, fibrosis, pulmonary hypertension, and the related inflammation [6, 31, 119, 120]. In pulmonary arterial smooth muscle cells, the inactivation of the Hippo/LATS1 pathway promotes proliferation and resistance to apoptosis through the YAP–fibronectin–integrin‐linked kinase 1 signaling axis. This process contributes to the advancement of pulmonary hypertension [121]. Discoidin domain receptor 1 (DDR1) can inhibit the phosphorylation of LATS1 and boost the activation and expression of YAP in a phase separation‐dependent manner. This mechanism helps in modulating the phenotype of vascular smooth muscle cells [122]. Vascular extracellular matrix sclerosis plays a role in promoting pulmonary hypertension by directly supporting vascular cell growth and migration through YAP/TAZ‐mediated glutamine metabolism and adhesion. Importantly, this process occurs independently of the Hippo pathway [123] (Figure 2).

#### 2.7.2 Atherosclerosis

Atherosclerosis is defined by diminished vascular elasticity due to lipid buildup and fibrous tissue growth [124, 125, 126]. The activity of YAP/TAZ in endothelial cells is indeed influenced by different blood flow patterns. Inhibiting YAP/TAZ has been shown to suppress inflammation and can potentially delay the onset of atherosclerosis [127, 128]. Under stable laminar flow conditions, LATS1/2‐mediated YAP phosphorylation by the Hippo kinase pathway inhibits YAP activation in endothelial cells. This process plays an anti‐inflammatory role in atherosclerosis [129, 130]. Studies indicate that cleaving DDR1 to form liquid biomolecular condensates, which then interact with the 14‐3‐3 protein epsilon (YWHAE), can trigger YAP nuclear translocation and potentially promote atherosclerosis. Importantly, this process may occur independently of the Hippo pathway [131]. Furthermore, oscillating shear stress can induce tyrosine phosphorylation and lead to robust, sustained nuclear translocation of YAP in integrin α5β1‐dependent endothelial cells. This process promotes atherosclerosis [132]. The BTB and CNC homology 1–YAP transcription network also plays a crucial role in driving vascular inflammation and atherosclerosis [133] (Figure 2).

#### 2.7.3 Myocardial Infarction

Acute myocardial infarction denotes heart function loss due to ischemic necrosis of the myocardium from acute coronary artery blockage [134, 135, 136]. Postinfarction, YAP activation in cardiomyocytes fosters cardiomyocyte proliferation and survival [137, 138, 139, 140, 141, 142]. Several studies have identified signaling pathways that directly regulate YAP in myocardial infarction. Receptor tyrosine‐protein kinase erbB‐2 (Erbb2)‐mediated YAP mechanotransduction signals drive a process similar to EMT in cardiomyocytes with heart failure [143]. Elevated kynurenine levels induced by acute inflammation in cardiac injury can trigger cardiomyocyte proliferation through the cytoplasmic aryl hydrocarbon receptor (SRC)–YAP/extracellular signal‐regulated kinase (ERK) pathway [144]. Neonatal heart regeneration and repair depend on TLR3 to promote glycolysis‐dependent YAP1 activation, leading to miR‐152 expression targeting DNA methyltransferase 1/p27kip1 [145]. α‐Catenins regulate YAP to boost cell proliferation for myocardial regeneration postinjury [146]. Glycoprotein 130 (gp130) activation spurs YAP via Src, enhancing cardiomyocyte proliferation postcardiac injury [147]. AT‐rich interactive domain‐containing protein 1A (ARID1A) directly binds and inhibits the transcriptional coactivators YAP and TAZ, which are known to drive proliferation. By competing with YAP/TAZ for binding with TEAD, ARID1A hinders adult cardiomyocyte regeneration [148]. Dystroglycan 1 binds directly to Yap, repressing mouse cardiomyocyte proliferation [141]. YAP and TAZ alleviate postinfarction inflammation by recruiting Tregs [149].

YAP also contributes to reperfusion injury postmyocardial infarction. Neurofibromatosis Type 2 (NF2) exacerbates cardiac ischemia–reperfusion injury by affecting key Hippo pathway components, activating MST1, and suppressing YAP [150]. The Hippo pathway exacerbates myocardial ischemia/reperfusion injury by impeding YAP–Forkhead box protein O1 (FoxO1) function [151]. Furthermore, YAP activation boosts miR‐206 expression to regulate myocardial hypertrophy and survival, thereby safeguarding the heart from ischemia/reperfusion injury [152]. YAP curtails myocardial ischemia/reperfusion injury‐induced ferroptosis by enhancing the transcription of neural precursor cell expressed developmentally downregulated 4‐2, which leads to the subsequent ubiquitination and degradation of Acyl coenzyme A (Acyl‐CoA) synthetase long‐chain family member 4 (ACSL4) [153] (Figure 2).

#### 2.7.4 Other Heart Inflammatory Diseases

Piezo1 activation stimulates calcium‐dependent YAP activation, modulates glutaminase (GLS1)‐mediated glutamine hydrolysis, and drives osteogenic differentiation through histone acetylation of Runt‐related transcription factor 2 promoters, ultimately leading to calcified aortic valve disease [154]. Ang II elevates galectin‐3 expression by enhancing YAP nuclear localization in vascular endothelium. This process contributes to endothelial dysfunction and hypertension [155]. Pressure overload triggers the YAP–TEAD1–Oncostatin M positive feedback loop, inducing cardiomyocyte dedifferentiation and heart failure [156, 157].

### 2.8 liver Inflammatory Diseases

YAP activation is crucial for liver cell proliferation and disease progression. By counteracting hepatocyte nuclear factor 4 alpha (HNF4α) and modulating immune infiltration and vascular structure in the liver, YAP stimulates hepatocyte proliferation and tissue remodeling. Additionally, it drives macrophage polarization, reduces T lymphocytes, and induces endothelial cell dedifferentiation into proliferative progenitor cells [158]. Arid1a enhances the interaction between YAP and genes that enrich liver progenitor cells, facilitating liver regeneration postinjury [159, 160].

#### 2.8.1 Liver Ischemia–Reperfusion Injury

Liver ischemia–reperfusion injury results from the reperfusion of liver tissue following ischemia, a primary cause of organ failure post liver transplantation [161, 162, 163]. Within the Hippo pathway, TNFAIP3 interacting protein 3 activates YAP by facilitating the ubiquitination and degradation of LATS2, thereby mitigating cell death and inflammation induced by liver ischemia/reperfusion injury [164]. Reticulon 4B (Nogo‐B) triggers macrophage‐associated innate inflammation and liver ischemia–reperfusion injury by activating the MST‐mediated Hippo/YAP pathway [165]. Additionally, YAP activation can reduce oxidative stress and the innate immune response of hepatic cells post liver ischemia–reperfusion injury, while also inhibiting hepatic stellate cell activation [166]. Myelogenic inhibitory cells recruited by the YAP/TEAD1–CXCL17 signal from endothelial cells mitigate liver ischemia–reperfusion injury [167]. Hepatocytes release CD47 via extracellular vesicles in a YAP‐dependent manner, inhibiting dendritic cell activation and contributing to the amelioration of liver ischemic reperfusion injury [168] (Figure S2A).

#### 2.8.2 Hepatitis

Hepatitis, instigated by viruses, bacteria, alcohol, and various factors, results in liver cell destruction and impaired function [169, 170, 171]. Persistent inflammation in hepatitis is closely linked to liver cancer development [172, 173]. The Hippo/YAP pathway undergoes significant dysregulation in hepatitis, leading to uncontrolled YAP activation in liver cells [174]. FK506 binding protein 5 interacts with MST1 to inhibit hepatic YAP phosphorylation, facilitating YAP nuclear translocation and TEAD1 activation. This process promotes increased CXCL1 expression and leads to neutrophil infiltration in alcohol‐related liver disease [175]. NOB induces autophagy to activate the Hippo/YAP pathway, inhibiting EMT of hepatocytes during hepatic fibrosis [176]. LATS2 downregulation in the liver triggers sterol regulatory element‐binding protein (SREBP) activation and excessive cholesterol accumulation, promoting spontaneous fatty liver development [177]. Deletion of breast cancer susceptibility gene 1 associated protein protein outside the Hippo pathway reduces YAP phosphorylation, increasing the expression of YAP target genes that drive hepatocyte proliferation, cell death, and inflammation [178]. Liver damage raises YAP/TAZ/cysteine‐rich angiogenic inducer 61 levels in liver cells, attracting macrophages and promoting inflammation and fibrosis [179].

In hepatitis, mesenchymal stem cells influence macrophages to activate the Hippo pathway, promoting direct interaction between YAP and β‐catenin, controlling NLRP3 activation, and inducing the M2 phenotype [180]. Macrophages can also regulate hepatic steatosis through the STING–YAP axis independently of the Hippo pathway, reprogramming lipid metabolism in the transmembrane protein 205/mitase2/protein dithiomerase‐dependent pathway [181]. Ceramide acts as a crucial regulator of YAP/TAZ signaling and hepatic stellate cell activation, thereby promoting liver fibrosis [182]. The purinergic receptor P2Y14 ligand triggers ERK and YAP signaling in hepatic stellate cells, leading to ERK‐dependent hepatic stellate cell activation and liver fibrosis [183]. YAP in Kupffer cells enhances proinflammatory cytokine production, promoting nonalcoholic steatohepatitis development [184] (Figure S2B).

### 2.9 Colitis

**FIGURE 3:**
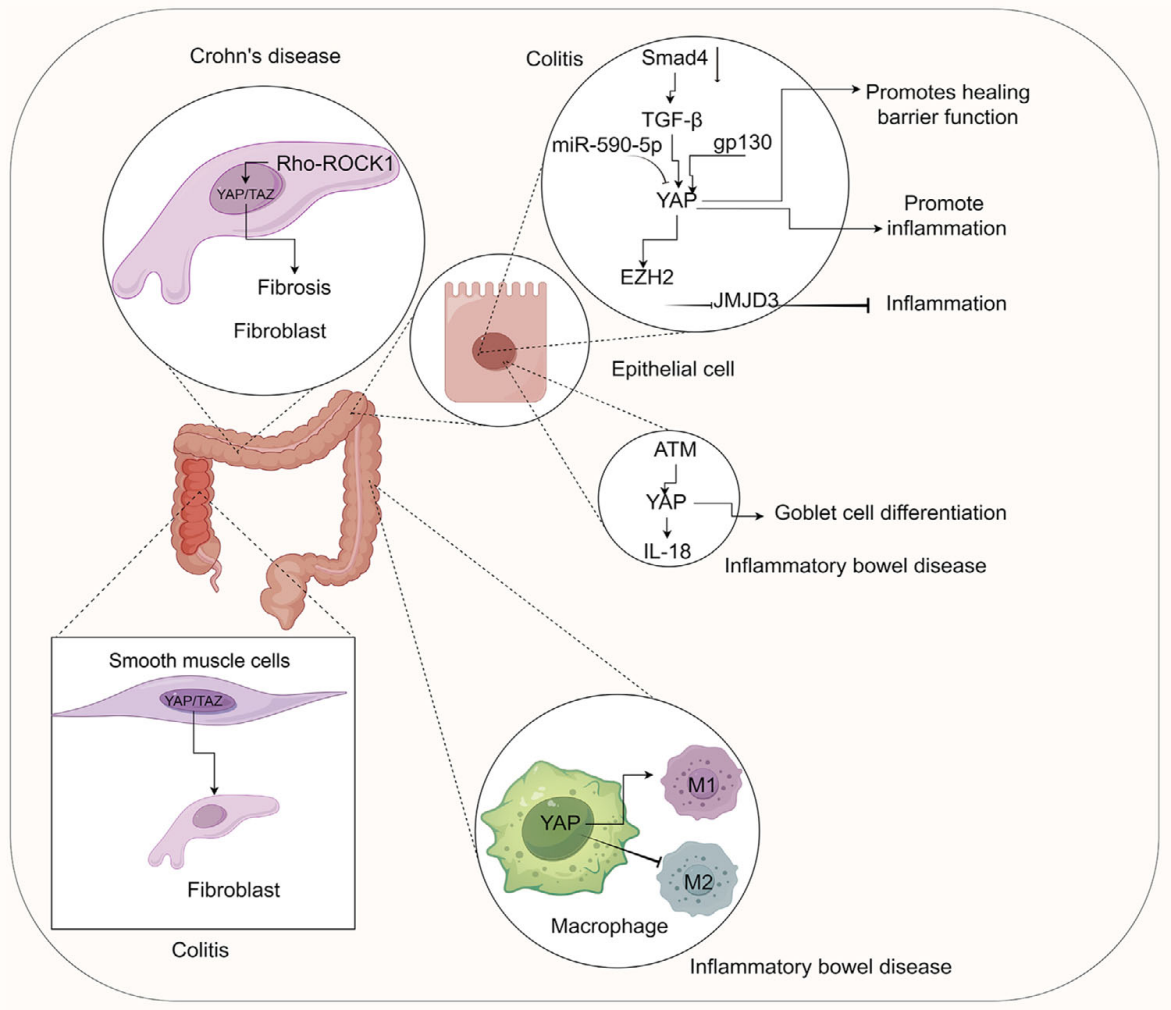
Molecular mechanism of YAP in colitis. (A) Mechanism of YAP in epithelial cells, epithelial cells, fibroblasts; smooth muscle cells and macrophage of colitis. ATM, ataxia telangiectasia mutated; EZH2, enhancer of zeste homolog 2; gp130, glycoprotein 130; JMJD3, Jumonji domain‐containing protein 3; Smad4, mothers against decapentaplegic homolog 4; TGF‐β, transforming growth factor‐β; YAP, Yes‐associated protein. The figure is created by Figdraw.

Colitis, an inflammatory bowel disease, arises from various causes such as ulcers, bleeding, and inflammatory edema lesions, encompassing acute colitis, Crohn's disease [185, 186], and ulcerative colitis [187, 188]. Colitis primarily regulates YAP through non‐Hippo pathways, influencing disease progression. TGF‐β1 directly upregulates YAP post‐Smad4 deletion, mediating TGF‐β1's effects on colon inflammation and related tumorigenesis via interaction with Smad2/3 [189]. During intestinal mucosal injury, gp130 binds to tyrosine kinases Src and Yamaguchi sarcoma viral (v‐yes) oncogene homolog (Yes), activating YAP phosphorylation at receptor conjugation, inducing its stabilization and nuclear translocation, thereby promoting healing and maintaining barrier function [190]. YAP inhibits colitis inflammation by recruiting enhancer of zeste homolog 2 to suppress Jumonji domain containing 3 (JMJD3) epigenetic silencing [191]. Activation of YAP/TAZ in acute colitis is essential for transforming intestinal smooth muscle cells into fibroblasts [192]. miR‐590‐5p suppresses colon intestinal inflammation and colorectal cancer cell tumorigenesis by targeting YAP [193]. YAP/TAZ plays a pivotal role in the self‐renewal and regeneration of the midgut epithelium in inflammatory bowel disease [194]. Telomere dysfunction‐induced activation of the ATM/YAP1/pro‐IL‐18 pathway may contribute to inflammatory bowel disease progression [195]. Fibrosis and tissue sclerosis in inflammatory bowel disease upregulate YAP expression, extending olfactomedin‐4(+) cells to the chorioid region, promoting preferential differentiation of intestinal stem cells into goblet cells [196]. YAP in inflammatory bowel disease hinders M2 macrophage polarization while enhancing M1 macrophage activation, exacerbating intestinal inflammation [197]. The Rho–ROCK1 signaling pathway in intestinal fibroblasts promotes YAP/TAZ activation, fostering intestinal fibrosis in Crohn's disease [198] (Figure 3).

### 2.10 Nerve Damage and Cerebral Hemorrhage

**FIGURE 4:**
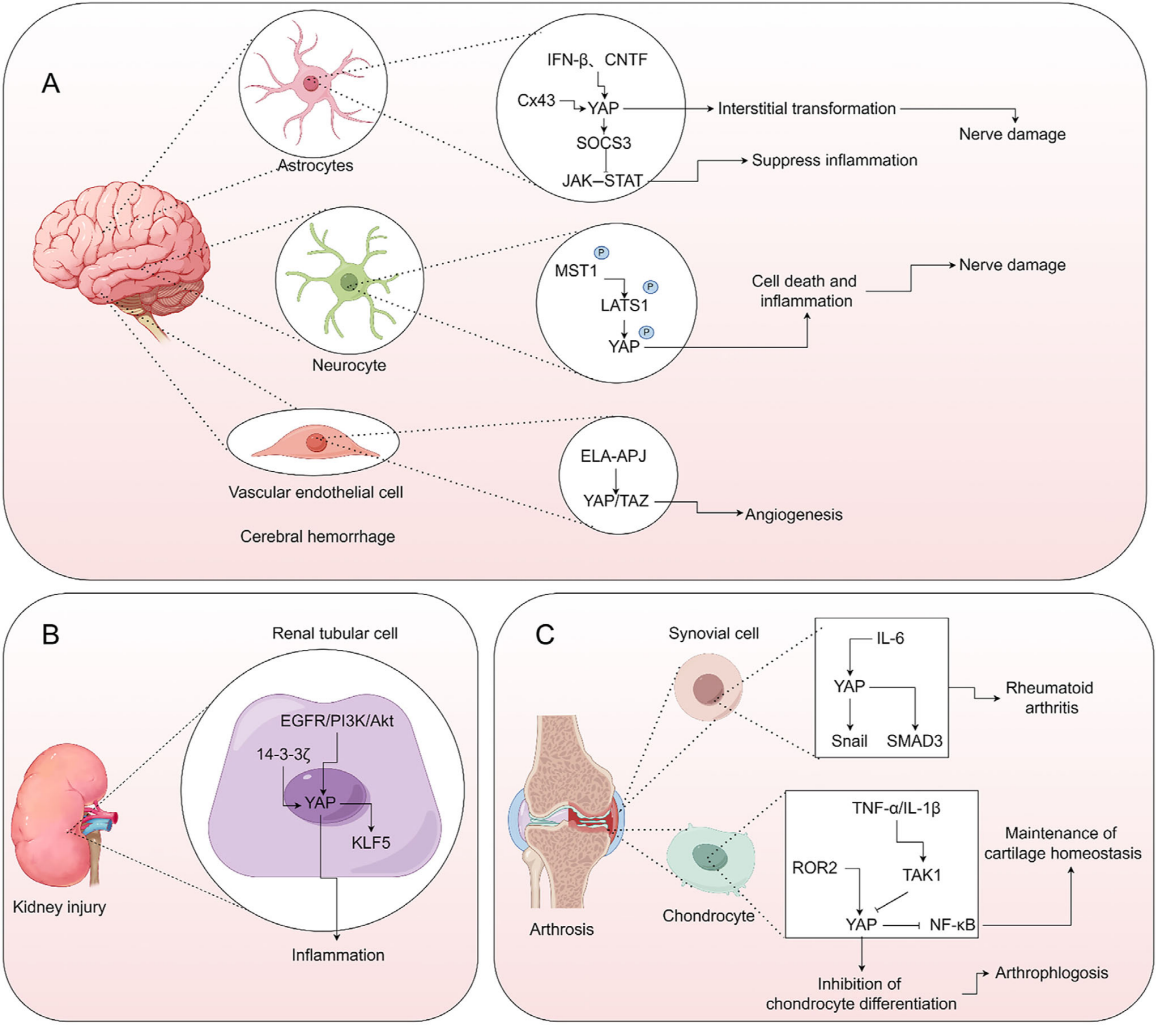
Molecular mechanism of YAP in nerve damage and cerebral hemorrhage. (A) Mechanism of YAP in neurocyte, astrocytes, and vascular endothelial cells of nerve damage and cerebral hemorrhage. (B) Mechanism of YAP in renal tubular cells of kidney injury. (C) mechanism of yap in synovial cells and chondrocyte of osteoarthritis. Akt, protein kinase B; CNTF, ciliary neurotrophic factor; Cx43, connexin 43; EGFR, epidermal growth factor receptor; ELA–APJ, apelin–APJ; IFN‐β, interferon‐β; JAK–STAT, Janus kinase–signal transducer and activator of transcription; KLF5, Kruppel‐like factor 5 protein; LATS1, large tumor suppressor homolog 1; MST1, mammalian sterile 20‐like protein kinase 1; NF‐κb, nuclear factor kappa B; PI3K, phosphoinositide 3‐kinase; ROR2, receptor tyrosine kinase‐like orphan receptor 2; SMAD3, mothers against decapentaplegic homolog 3 protein; SOCS3, suppressors of cytokine signaling 3; TAK1, transforming growth factor‐β‐activated kinase 1; TNF‐α, tumor necrosis factor‐α; YAP, Yes‐associated protein. The figure is created by Figdraw.

In the Hippo pathway during cerebral hemorrhage, inhibiting MST1 can decrease neuronal cell death and the inflammatory response by reducing p‐LATS1 and p‐YAP activation [199]. Various other mechanisms of YAP activation are also involved in disease progression. Lowering connexin 43 levels facilitates YAP nuclear translocation, promoting astroglia‐interstitial transformation in intracerebral hemorrhage [200]. Activation of YAP by IFN‐β or ciliary neurotrophic factor induces suppressor of cytokine signaling 3, which inhibits excessive activation of the Janus kinase (JAK)‐signal transducer and activator of transcription (STAT) pathway and reactive astrocyte proliferation, thereby exerting a negative regulatory effect on neuroinflammation [201]. The elastase–apelin receptor pathway activates YAP/TAZ signaling, enhancing poststroke angiogenesis [202] (Figure 4A).

### 2.11 Kidney Injury

Kidney injury, comprising acute kidney injury and chronic kidney disease, represents a decline in kidney function caused by a variety of factors [203, 204, 205, 206]. Multiple studies have elucidated the involvement of YAP in inflammation through non‐Hippo pathways. By regulating YAP, 14‐3‐3ζ can prevent the progression from acute renal insufficiency to chronic renal disease [207]. The Kruppel‐like factor 4 (KLF4)–YAP pathway activation promotes the transition from ischemia–reperfusion‐induced acute kidney injury to chronic kidney disease [208]. In renal ischemia–reperfusion injury, epithelial cell regeneration is promoted through the activation of the epidermal growth factor receptor (EGFR)–phosphoinositide 3‐kinase (PI3K)–protein kinase B (Akt) pathway and subsequent YAP activation [209]. Podocyte injury in the kidney enhances YAP's transcriptional coactivator function, leading to increased expression of YAP target genes and fibrotic extracellular matrix‐associated proteins [210]. Tubular YAP activation following renal injury promotes KLF5 transcription, triggering tubular cell hyperproliferation, tubular injury, and renal inflammation [211, 212] (Figure 4B).

### 2.12 Osteoarthritis

Osteoarthritis primarily affects joint bone and cartilage, leading to cartilage degeneration and bone hyperplasia damage [213, 214]. YAP activation, independent of the Hippo pathway, plays a pivotal role in osteoarthritis. Within fibroblast‐like synoviocytes, protein tyrosine phosphatase nonreceptor type 14 (PTPN14) and YAP facilitate the nuclear translocation of SMAD3, contributing to the progression of rheumatoid arthritis [215]. IL‐6 activates YAP through JAK, promoting YAP–Snail family transcriptional repressor 1 (SNAIL) interactions that drive the pathogenic transformation of rheumatoid arthritis synovial fibroblasts, ultimately leading to joint destruction [29]. Inhibiting receptor tyrosine kinase‐like orphan receptor 2 (ROR2) enhances cartilage integrity and alleviates pain in osteoarthritis by downregulating YAP signaling [216]. In the pathogenesis of osteoarthritis, TNF‐α and IL‐1β induce TGF‐β‐activated kinase 1‐mediated phosphorylation, resulting in YAP/TAZ degradation in chondrocytes [217] (Figure 4C).

### 2.13 Sepsis

Sepsis occurs due to systemic infection and organ dysfunction caused by pathogenic microorganisms infiltrating the bloodstream, multiplying, and releasing abundant endotoxins [218, 219, 220]. In septicemia, lactate triggers high mobility group box 1 (HMGB1) acetylation, which boosts endothelial permeability by inhibiting the deacetylase Sirtuin 1 via Hippo/YAP signaling, recruiting it to the nucleus of GPR81 in macrophages [221]. Moreover, YAP regulates endothelial cell activation by blocking TNF receptor‐associated factor 6‐mediated NF‐κB activation, thereby mitigating vascular inflammation in sepsis [27] (Figure S3A).

### 2.14 Diabetes

Diabetes mellitus, a metabolic disorder characterized by hyperglycemia due to inadequate insulin secretion and utilization [222, 223], involves non‐Hippo pathway‐dependent YAP activation, which drives the progression of diabetes‐related atherosclerosis. SNO‐guanine nucleotide binding protein subunit alpha I2 accelerates diabetes‐related atherosclerosis by interacting with C–X–C motif chemokine receptor 5 (CXCR5) and activating YAP‐mediated endothelial inflammation [224]. In obesity and diabetes, the AMPK–YAP–c‐Jun N‐terminal kinase (JNK) axis upregulates YAP target genes and inflammatory markers in endothelial cells, contributing to vascular dysfunction [225] (Figure S3B).

Similarly, non‐Hippo pathway‐dependent YAP activation also contributes to diabetic nephropathy. The EGFR–PI3K–Akt–cAMP–response element binding protein (CREB) signaling pathway triggers YAP gene expression, nuclear translocation, and TEAD interaction, exacerbating diabetic nephropathy [226]. YAP inactivation in renal podocytes may reduce Wilms tumor 1 expression, furthering diabetic nephropathy progression [227]. The binding of YAP/TAZ to and stabilization of neuroblastoma V‐Myc myelocytomatosis viral oncogene homolog protein amplify its transcriptional activity, culminating in mesangial cell injury and the progression of diabetic nephropathy [228] (Figure S3B).

### 2.15 Other Inflammatory Diseases

SET domain containing 7 regulates the altered differentiation and proliferation dynamics of the intestinal epithelium during worm infection by modulating the Hippo/YAP developmental signaling pathway [229]. Hypoxia suppresses LATS1‐induced YAP1 expression, promoting endometriosis [230]. In adipocytes, TNF‐α and IL‐1β induce YAP/TAZ nuclear translocation by activating RhoA‐mediated actomyosin contractility and enhance YAP/TAZ‐mediated transcriptional regulation by activating JNK and activator protein 1 (AP‐1) [231]. YAP activation and JNK/AP‐1 upregulation can exacerbate the inflammatory response in periodontitis [232].

## 3 YAP in Cancers

YAP is crucial for tumor cell survival, governing cell proliferation and influencing tumor immune and inflammatory states [233, 234, 235, 236, 237, 238]. In the Hippo signaling pathway, O‐GlcNAc transferase‐induced O‐GlcNAcylation of YAP disrupts its interaction with the upstream kinase LATS1, preventing phosphorylation and activating its transcriptional activity [239]. Furthermore, in non‐Hippo pathway‐dependent YAP activation, RASSF1A expression regulates TGF‐β‐induced YAP1/SMAD2 interaction, exerting a cancer‐inhibiting effect [240]. Mechanical sensing signals activate the YAP–TAZ complex, modulate disabled homolog 2, mitogen‐responsive phosphoprotein in macrophages, and regulate integrin circulation in the three‐dimensional tumor tissue matrix, impacting extracellular matrix remodeling and metastasis [241]. Leukemia inhibitory factor receptor alpha inhibits YAP expression to hinder tumor metastasis [242]. YAP activation in Tregs enhances TGF‐β/SMAD expression, contributing to antitumor immune function [243].

### 3.1 YAP as an Oncogene

YAP, functioning as an oncogene, has the potential to drive cell proliferation by modulating cell cycle progression, cellular senescence, and autophagy. This, in turn, can trigger angiogenesis and the onset of EMT, ultimately culminating in the development of the characteristic pathological morphology associated with tumors. Once YAP binds to the TEAD transcription factor, it is capable of facilitating the expression of cyclin. This, in turn, fuels the proliferation of tumor cells and enables them to perpetuate division [244, 245]. YAP can also sustain the continuous proliferation of tumor cells by suppressing cell senescence‐associated signaling pathways [246, 247]. Additionally, YAP can promote autophagy through its interaction with the promoter regions of autophagy‐related genes to modulate crucial proteins within the autophagy signaling pathway [248, 249, 250, 251]. YAP can also activate the expression of the vascular endothelial growth factor (VEGF) gene, spurring the proliferation and migration of endothelial cells and the formation of vascular lumens. This vascular facilitation paves the way for tumor cells to infiltrate the bloodstream and embark on further distant metastasis [252, 253]. YAP can also induce EMT in tumor cells and drive the EMT process by activating certain EMT‐related transcription factors [254]. Overall, YAP assumes a significant role in the nascent stage of tumor cell metastasis and augments the migratory and invasive capabilities of these cells.

### 3.2 YAP in the Tumor Microenvironment

Within the tumor microenvironment (TME), YAP exerts multiple immunosuppressive effects. It can suppress the expression of crucial immune‐activating molecules. This manipulation relegates T cells to a state of immune exhaustion, consequently undermining their antitumor immune response [255, 256]. Additionally, YAP‐activated Tregs secrete inhibitory cytokines like IL‐10 and TGF‐β, which impede the antitumor activities of other immune cells and foster an immunosuppressive milieu conducive to tumor cell survival [257, 258, 259]. Moreover, YAP can upregulate the expression of certain glycolytic‐related genes. By doing so, it facilitates the preferential uptake and utilization of glucose by tumor cells, thereby sustaining their growth and proliferation [260, 261, 262]. Such an interaction further underlines the intimate nexus between YAP and TME metabolism, highlighting the complex web of molecular crossovers that underpin tumor progression and the need for comprehensive strategies to target YAP and its associated pathways in cancer treatment.

### 3.3 YAP as a Link Between Inflammation and Cancer

Inflammation emerges as a potent driver for YAP activation, potentially promoting tumorigenesis. Once activated in an inflammatory environment, YAP interacts with cyclin, initiating uncontrolled cell proliferation [263]. This unchecked proliferation sets the stage for the accumulation of genetic mutations in cells, increasing the likelihood of tumorigenesis. YAP also boosts the production of antiapoptotic proteins, impeding apoptosis and potentially paving the way for cellular transition into tumor cells [264, 265]. Within inflammatory sites, YAP induces the expression of VEGF, promoting the formation of new blood vessels [253, 266]. These vascular networks provide essential nutrients and oxygen to tumor cells, aiding their dissemination through the bloodstream and subsequent metastasis.

Furthermore, YAP activation in tumor cells prompts the secretion of various inflammatory mediators. Elevated YAP levels are associated with increased secretion of these mediators [6, 267, 268], attracting immune cells to form inflammatory hotspots within the TME. YAP also stimulates fibroblasts to release collagen and other extracellular matrix components, altering the migration and function of immune cells and other cell types [269, 270]. This perpetuates the inflammatory response, creating a feedback loop between inflammation and tumorigenesis. Additionally, tumor cells utilize YAP to control exosome, releasing inflammatory mediators and signaling molecules to neighboring cells [271, 272]. This process either triggers or sustains an inflammatory reaction that fosters tumor growth and advancement. The intricate interplay involving YAP, inflammation, and tumorigenesis deepens our comprehension of cancer pathophysiology and unveils promising therapeutic pathways for future investigation.

### 3.4 Head and Neck Cancer

Head and neck tumors, predominantly thyroid, nasopharyngeal, oral, laryngeal, and sinus cancers. Variations in anatomical location, pathological types, and mechanisms among these tumors result in diverse treatment approaches [273, 274, 275]. Nasopharyngeal cancer, for instance, responds well to radiotherapy [276], while laryngeal, oral, and sinus cancers typically require a combination of surgical intervention, postoperative radiotherapy, and chemotherapy for effective management [277, 278, 279, 280]. These distinct therapeutic strategies highlight the importance of tailored treatment regimens based on the specific molecular pathologic mechanisms of each head and neck malignancy. YAP1 is a key promoter of head and neck squamous cell carcinoma [281]. The interaction between actin like 6A and p63 activates Hippo–YAP via WW domain containing 1, contributing to the promotion of head and neck squamous cell carcinoma [282].

#### 3.4.1 Nasopharyngeal Carcinoma

Nasopharyngeal carcinoma is a malignant epithelial tumor that originates from the nasopharyngeal mucosa [283, 284, 285]. Recent studies have identified Epstein–Barr virus infection and smoking as contributing factors to its pathogenesis. Typically, nasopharyngeal cancer presents with early lymph node enlargement in the neck and epistaxis. While radiotherapy remains the primary treatment for nasopharyngeal carcinoma, surgery has increasingly become a viable option for recurrent cases in recent years [286, 287]. YAP plays a crucial role in the development of nasopharyngeal carcinoma. circRILPL1 inhibits the LATS1–YAP kinase cascade in the Hippo pathway by binding to and activating Rho‐associated coiled‐coil containing protein kinase 1 (ROCK1), leading to reduced YAP phosphorylation [288]. Cytoplasmic leukemia inhibitory factor promotes vascular dissemination and local invasion of nasopharyngeal carcinoma by modulating the YAP1–focal adhesion kinase (FAK)/paxillin signaling pathway [289] (Figure S4A).

#### 3.4.2 Thyroid Cancer

Thyroid cancer primarily stems from malignant tumors of the thyroid follicular or parafollicular epithelium. The most common type is papillary carcinoma, with undifferentiated carcinoma carrying the poorest prognosis [290, 291]. Initially asymptomatic, thyroid cancer can progress to cause breathing difficulties, hoarseness, and cervical lymph node metastasis in advanced stages [292, 293]. YAP plays a pivotal role in the development of thyroid cancer. Ubiquitin carboxyl terminal hydrolase L3 stabilizes YAP through deubiquitinating activity, enhancing anaplastic thyroid cancer progression, stemness, metastasis, and increasing cell sensitivity to chemotherapy [294]. Neurofibromatosis type 2 (NF2)/merlin inactivation boosts RAS protein activation by enhancing YAP/TEAD activity [295]. YAP–TEAD1 induces platelet‐derived growth factor‐BB (PDGF‐BB) transcription, while PDGF‐BB, via its receptor PDGFR, stabilizes YAP and facilitates YAP nuclear translocation [296] (Figure S4A).

### 3.5 Malignant Peripheral Nerve Sheath Tumors

Malignant peripheral nerve sheath tumors originate from peripheral nerve Schwann cells [297, 298]. Their location varies, and symptoms typically manifest as localized masses, with potential for metastases to distant lymph nodes and the bloodstream [299]. Excessive TAZ/YAP activity in Schwann cells due to LATS1/2 deletion in the Hippo pathway could lead to the development of malignant peripheral nerve sheath tumors [300, 301, 302] (Figure S4B).

### 3.6 Melanoma

Melanoma, a malignant tumor derived from melanocytes, can arise in various locations including the skin (limbs, toes), mucous membranes (nasal and oral cavities, digestive tract), and the uvea of the eye [303, 304]. Mutations within melanoma cells trigger extensive proliferation, fostering the spread of cancer cells and their infiltration into organs. Historically, surgical resection has been the cornerstone of melanoma treatment, often complemented by therapies like immunotherapy [305, 306]. Recent studies have elucidated the significant involvement of YAP in both the development and progression of melanoma, shedding light on potential therapeutic targets for this aggressive cancer. The YAP–TEAD interaction is crucial in uveal melanoma [307]. Gαq facilitates YAP‐dependent growth in uveal melanoma cells [308]. Guanine nucleotide‐binding protein, Q polypeptide (GNAQ) activates YAP via FAK, driving uveal melanoma progression [309, 310, 311]. The β‐catenin–YAP signaling axis activates stromal fibroblasts, fostering melanoma progression [312] (Figure S4C).

### 3.7 Breast Cancer

Breast cancer predominantly arises from mammary epithelial cells and is intricately linked to female hormones [313, 314]. This prevalent malignancy in women is stratified into noninvasive and invasive subtypes, with noninvasive cases generally exhibiting a more favorable prognosis compared with the metastatic potential of invasive forms. Early manifestations such as breast masses, nipple discharge, and enlarged axillary lymph nodes mark the initial stages of the disease, which can progress to systemic metastasis over time [315, 316]. Treatment modalities for breast cancer typically encompass a multidisciplinary approach involving surgery, chemotherapy, and endocrine therapy, tailored to individual patient needs [317, 318, 319]. Recent research has unveiled the pivotal role of YAP in the pathogenesis of breast cancer, shedding light on potential therapeutic targets and avenues for further exploration in the quest to enhance treatment efficacy and patient outcomes. In the realm of cancer research, lncRNA SNHG9 acts by inhibiting the Hippo pathway through LATS1 [320], while ROR1–human epidermal growth factor receptor 3 (HER3)–lncRNA LLGL2–MAYA–NOP2/Sun RNA methyltransferase 6 is involved in regulating the Hippo–YAP pathway to control bone metastasis in breast cancer [321]. Moreover, seven in absentia homolog 2 inhibits LATS2 to stimulate YAP [322], and LATS1/2 maintains estrogen receptor alpha (Erα) expression by inhibiting YAP/TAZ, thereby promoting the growth of ERα+ breast cancer cells. YAP1 and TEAD4 can also serve as ERα cofactors to further boost the growth of ERα(+) breast cancer [323, 324]. Additionally, PA directly interacts with Hippo components LATS and NF2, disrupting the formation of the LSAT–MOB1 complex and NF2‐mediated translocation and activation of LATS membrane, ultimately leading to the activation of YAP [325]. Notably, there are instances where direct stimulation of YAP occurs independently of the Hippo pathway in breast cancer. TNF‐α in breast cancer is known to promote IκB kinase‐mediated phosphorylation and activation of YAP, with YAP/TEAD and p65 proteins working synergistically to regulate hexokinase 2 expression and promote macrophage migration [326]. Loss of cerebral cavernous malformation 3 leads to YAP/TAZ activation [327], and FAT1 deletion significantly increases cyclin‐dependent kinase 6 through YAP and TAZ transcription factors [245]. The SREBP/mevalonate pathway is instrumental in promoting YAP/TAZ activation [328]. Furthermore, YAP is involved in regulating protein levels in anillin (ANLN), diaphanous‐related formin 3, and myosin light chain 9 to maintain the phenotype of breast cancer‐associated fibroblasts [20]. YAP's ability to promote amphiregulin contributes to cell proliferation and migration [329], and lncRNA MALAT1 binds to TEAD, preventing it from binding to its coactivator YAP and the target gene promoter [330]. Last, MYC enhances AMPK and inhibits YAP/TAZ activity in breast tumors [331] (Figure S4D).

### 3.8 Gastric Cancer

**FIGURE 5:**
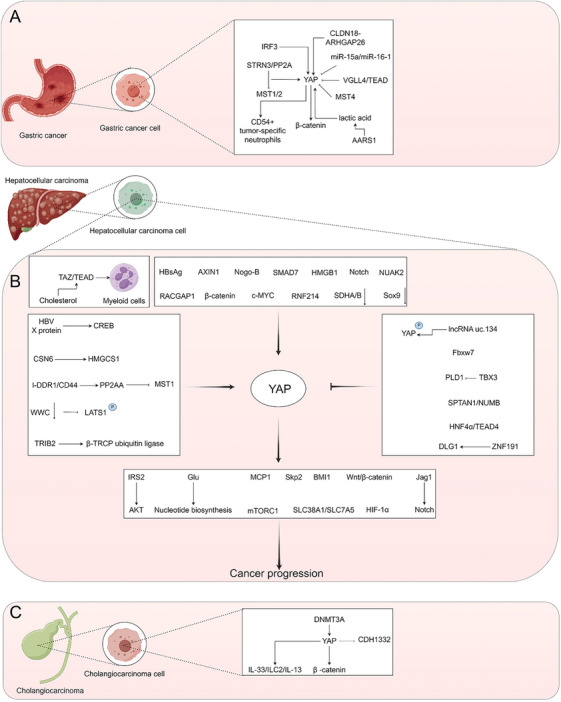
Molecular mechanism of YAP in gastric cancer, liver cancer and cholangiocarcinoma. (A) Mechanism of YAP in gastric cancer. (B) Mechanism of YAP in liver cancer. (C) Mechanism of YAP in melanoma. (D) Mechanism of YAP in cholangiocarcinoma. AKT, protein kinase B; AXIN1, axonemal assembly protein 1; BMI1, B lymphoma Mo‐MLV insertion region 1; c‐Myc, cellular myelocytomatosis oncogene; CD44, cluster of differentiation 44; CREB, cAMP response element binding protein; CSN6, COP9 signalosome 6; DLG1, discs large homolog 1; DNMT3A, DNA methyltransferase 3 alpha; Fbxw7, F‐box and WD repeat domain‐containing 7; Glu, glutamic acid; HBsAg, hepatitis B surface antigen; HBV, hepatitis B virus; HIF‐1α, hypoxia‐inducible factor‐1α; HMGCS1, 3‐hydroxy‐3‐methylglutaryl‐CoA synthase 1; HNF4α, hepatocyte nuclear factor 4 alpha; I‐DDR1, discoidin domain receptor 1; IL, interleukin; ILC2, type 2 innate lymphoid cells; IRF3, interferon regulatory factor 3; IRS2, insulin receptor substrate; Jag1, Jagged1; LATS1, large tumor suppressor homolog 1; MCP1, monocyte chemoattractant protein 1; MST1, mammalian sterile 20‐like protein kinase 1; MST4, mammalian sterile 20‐like protein kinase 4; mTORC1, mechanistic target of rapamycin complex 1; Nogo‐B, reticulon 4B; NUAK2, NUAK family kinase 2; PP2A, protein phosphatase 2A; PP2AA, protein phosphatase 2A; PLD1, phospholipase D1; RACGAP1, Rac GTPase‐activating protein 1; RNF214, ring finger protein 214; SDHA/B, succinate dehydrogenase complex subunit A/B; Skp2, S‐phase kinase‐associated protein2; SLC38A1, solute carrier family 38 member 1; SLC7A5, solute carrier family 7 member 5; SMAD7, mothers against decapentaplegic homolog 7; Sox9, SRY‐related HMG‐box 9 protein; SPTAN1, spectrin alpha nonerythrocytic 1; STRN3, striatin 3; TAZ, transcriptional coactivator with PDZ‐binding motif; TBX3, T‐box transcription factor 3; TEADs, TEA domain transcription factors; TRIB2, tripartite motif protein VGLL4, vestigial like family member 4; Wnt, wingless‐related integration site; WWC, WW domain containing cytoskeleton regulatory protein 1; YAP, Yes‐associated protein; ZNF191, zinc finger protein 191. The figure is created by Figdraw.

Gastric cancer, a malignancy originating in the stomach lining cells, typically progresses slowly with symptoms manifesting prominently only in advanced stages [332, 333]. The development of this cancer involves a complex interplay of genetic and environmental factors, including Helicobacter pylori infection, dietary patterns, smoking, and genetic predisposition. Mechanistically, gastric cancer onset is intricately linked to gene mutations, inflammatory responses, DNA damage, and metabolic irregularities that impede cell growth and apoptosis [334, 335, 336, 337, 338]. Treatment strategies for gastric cancer vary based on tumor stage and patient health. Surgery stands as the primary intervention for early‐stage cases, aiming at complete tumor removal. In advanced stages, a multidisciplinary approach combining surgery with chemotherapy and radiotherapy is commonly employed to alleviate symptoms, manage disease progression, and enhance patient survival rates. The evolving landscape of targeted therapies presents promising avenues for improved outcomes in gastric cancer management, offering novel treatment prospects for patients. The overexpression of YAP1 intensifies the aggressive behavior of gastric cancer cells [339]. In the context of the Hippo pathway in gastric cancer, Striatin 3 facilitates MST1/2 dephosphorylation through protein phosphatase 2A (PP2A), activating YAP and driving gastric cancer progression [340]. Moreover, MST4‐mediated YAP phospho–Thr83 signaling modulates YAP phospho–Ser127 signaling to suppress YAP activation in gastric cancer [341]. Independent activation of YAP, separate from the Hippo pathway, also plays a pivotal role in advancing gastric cancer. YAP and β‐catenin work synergistically to propel gastric cancer development through their physical interactions [342]. The claudin 18–Rho GTPase activating protein 26 fusion, an acquired DGC oncogene, accelerates gastric cancer growth by activating YAP signaling [343]. IRF3 enhances YAP expression, facilitating YAP–TEAD4 binding to promote gastric cancer development [344]. The rigidity of the extracellular matrix in gastric cancer dynamically influences DNA methylation in the YAP promoter region [345]. Alanyl‐tRNA synthetase 1 senses intracellular lactic acid, translocating to the nucleus to activate the YAP–TEAD complex, stimulating gastric cancer cell proliferation [346]. YAP/TAZ drives the differentiation of CD54+ tumor‐specific neutrophils in gastric cancer, fueling tumor progression [347]. Additionally, miR‐15a and miR‐16‐1 suppress gastric adenocarcinoma by reducing YAP levels [348]. Vestigial‐like protein 4 (VGLL4) inhibits YAP activity by competitively binding TEADs, hindering the advancement of gastric cancer [349] (Figure 5A).

### 3.9 Liver Cancer

Liver cancer, a prevalent malignant tumor globally, arises from liver cells, with hepatocellular carcinoma (HCC) and cholangiocarcinoma as typical forms [172, 350]. Its occurrence is frequently linked to cirrhosis, hepatitis B virus infection, alcoholism, liver steatosis, and genetic factors [351, 352]. Treatment avenues encompass surgical resection, liver transplantation, chemotherapy, radiotherapy, and targeted therapy. Surgical resection is the primary choice for early‐stage liver cancer, while liver transplantation emerges as a viable option for advanced cases. Chemotherapy and radiotherapy play crucial roles in symptom alleviation, disease management, and survival extension [353, 354]. The pathogenesis of liver cancer remains intricate and not yet fully elucidated. YAP and TAZ function as universally activated transcriptional regulators in liver tumors [4, 267, 355‐365]. lncRNA uc.134 impedes HCC progression by inhibiting cullin 4A‐mediated LATS1 ubiquitination and enhancing YAP(S127) phosphorylation [366]. The spectrin alpha, nonerythrocytic 1/NUMB axis restrains liver cancer cell growth through Hippo signaling [367]. Deletion of WW and C2 domain proteins hinders LATS1 phosphorylation, fostering YAP activation in the Hippo pathway, resulting in liver tissue overgrowth, inflammation, fibrosis, and liver cancer formation [368]. Chronic inflammation dampens epithelial splicing regulatory protein 2 expression in hepatocytes, impeding Hippo pathway activation, elevating downstream YAP/TAZ activity, and fostering hepatobiliary carcinoma in the presence of chronic liver injury [369]. Rac GTPase activating protein 1 diminishes Hippo activity, activating the YAP pathway to spur cell division and promote liver cancer cell proliferation [370]. Collagen I–DDR1 interacts with CD44, facilitating PP2A, catalytic subunit, alpha isoform (PP2AA) recruitment to MST1, counteracting Hippo signaling, activating YAP, and advancing HCC progression [371]. The Hippo signal curbs macrophage infiltration during TME formation by suppressing YAP‐dependent monocyte chemoattractant protein 1 expression, restraining liver cancer cell growth [372]. Hippo–yap signaling deters cell polyploidy and liver tumorigenesis via S‐phase kinase‐associated protein 2 [373]. Mst1/2 curtails Yap1 activity to impede liver tumor development [374]. Notably, activation of YAP independent of hippo pathway has also been commonly reported in liver cancer. Hepatitis B virus X protein spurs liver cancer cell growth in a CREB‐dependent manner through YAP [375]. TAZ expression in human liver tumors attracts numerous myeloid cells to infiltrate the liver, secreting proinflammatory cytokines via a TEAD‐dependent mechanism [376]. T‐box 3 inactivation of phospholipase D1 curbs YAP/TAZ activation, thwarting HCC progression [377]. F‐box and WD repeat domain‐containing 7 modulates HCC progression regulated by YAP through YAP ubiquitination and proteasome degradation [378]. The CD36–Nogo‐B–YAP pathway reshapes oxidized low‐density lipoprotein metabolism, inducing HCC linked to nonalcoholic fatty liver disease [361]. SMAD7 participates in liver cancer by activating the YAP/NOTCH signaling cascade, triggering bile duct cell signals and epithelial mesenchymal transformation [379]. HMGB1 induces YAP–HIF‐1α complex‐dependent aerobic glycolysis, fueling liver tumor progression [380]. SRY‐box 9 (Sox9) signaling proves essential for YAP to drive hepatic progenitor cell differentiation into biliary epithelial cells, with Sox9 deletion elevating YAP activity, fostering more aggressive HCC [18, 381]. COP9 signalosome subunit 6 stabilizes 3‐hydroxy‐3‐methylglutaryl‐CoA synthase 1 (HMGCS1) by counteracting speckle‐type POZ protein ubiquitin ligase, activating YAP1 to spur liver tumor growth [382]. Cholesterol boosts TAZ and TEAD2 interactions, promoting ANLN and kinesin family member 23‐mediated hepatocellular tumorigenesis [383]. β‐Catenin and Yap1 collaborate to propel tumor progression in hepatoblastoma [384]. Ring finger protein 214 triggers nonproteolytic ubiquitination of conserved lysine residues of TEADs, strengthening TEADs and YAP interaction, advancing liver cancer progression [385]. Axin 1 binds to YAP/TAZ in human HCC cells, regulating YAP/TAZ stability [386]. Succinate dehydrogenase complex flavoprotein subunit A/B reduction averts proteasome degradation of YAP/TAZ by modulating cullin1 neddylation, boosting HCC proliferation [387]. NUAK family kinase 2 (NUAK2) activates YAP by enhancing actin polymerization and myosin activity, fostering liver cancer cell proliferation [388]. Notch signaling spurs YAP/TAZ activation, while Wnt/β‐catenin signaling activation drives HCC formation [389]. HNF4α directly binds to TEAD4, competing with YAP1 for TEAD4, stalling liver cancer cell proliferation [390]. CREB bolsters YAP transcription by binding to the −608/−439 region of the YAP promoter, with YAP enhancing CREB protein stabilization by interacting with mitogen‐activated protein kinase (MAPK)14/p38 and β‐transduction repeats of BTRC, fueling liver cancer progression [391]. TRIB2 boosts stable YAP expression by interacting with beta‐transducin repeat‐containing protein (βTrCP) ubiquitin ligase [392]. The hepatitis B surface antigen (HBsAg)–YAP–B lymphoma Mo–MLV insertion region 1 homolog (BMI1) axis propels hepatitis B virus‐associated proliferative HCC progression [393]. Zinc finger protein 191 curtails YAP activation by upregulating discs large homolog 1, thwarting HCC metastasis [394]. Additionally, YAP1 directly heightens glutamine synthetase expression and activity, enhancing glutamine homeostasis and nitrogen isotope enrichment during new purine and pyrimidine biosynthesis, fostering liver tumorigenesis [395]. YAP1/TAZ regulates amino acid metabolism by upregulating olute carrier family 38 member 1 and SLC7A5, activating mechanistic target of rapamycin complex 1 to spur liver cancer cell proliferation [396]. YAP‐1 acts as the core mediator of fibrotic integrin β‐1 signaling, playing a crucial role in liver fibrosis [397]. YAP/TAZ activation amplifies AKT signaling by upregulating insulin receptor substrate 2 expression, promoting cancer progression [398]. YAP boosts Jagged 1 expression to activate Notch signaling, driving HCC proliferation [399] (Figure 5B).

### 3.10 Cholangiocarcinoma

Cholangiocarcinoma is a malignancy that originates from cells within the bile duct. It is typically classified into two primary types: intrahepatic cholangiocarcinoma and extrahepatic cholangiocarcinoma [400, 401]. The development of this cancer is intricate, involving genetic factors, inflammation, gallstones, cholangitis, biliary diseases, and parasitic infections. Common treatment options encompass surgical resection, chemotherapy, radiotherapy, targeted therapy, and immunotherapy. Surgical resection is considered the primary treatment for early‐stage cholangiocarcinoma, particularly for tumors that are localized within the ductal system [402, 403, 404]. Additionally, YAP plays a pivotal role in the pathogenesis of cholangiocarcinoma. In contrast to the conventional Hippo pathway, DNMT3A interacts with YAP/TAZ, leading to the transcriptional silencing of CDH1, thereby promoting gallbladder cancer metastasis [405]. The persistent activation of AKT and YAP triggers IL‐33/ILC2/IL‐13 pathways, fostering hepatic metastasis of cholangiocarcinoma [406]. YAP stimulates cholangiocarcinoma progression through TEAD‐dependent transcriptional activation and beta‐catenin interaction [407] (Figure 5C).

### 3.11 Pancreatic Cancer

**FIGURE 6:**
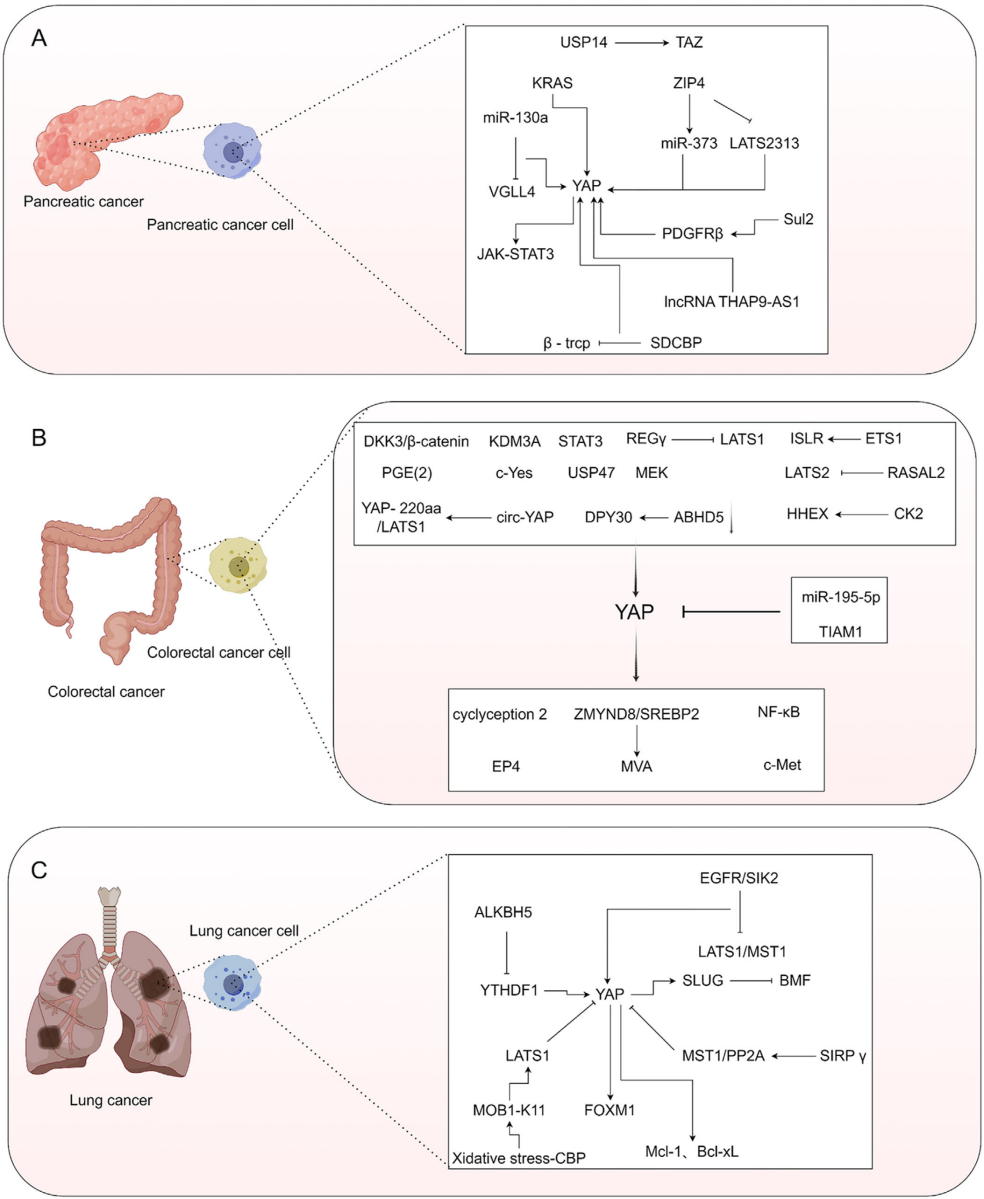
Molecular mechanism of YAP in pancreatic cancer, colorectal cancer, and lung cancer. (A) Mechanism of YAP in pancreatic cancer. (B) Mechanism of YAP in colorectal cancer. (C) Mechanism of YAP in lung cancer. ABHD5, abhydrolase domain containing 5; ALKBH5, AlkB homolog 5; β‐TrCP, β‐transducin repeat containing E3 ubiquitin protein ligase; Bcl‐xl, B‐cell lymphoma‐2 like protein 1, long isoform; BMF, Bcl‐2 modifying factor; c‐Met, mesenchymal–epithelial transition factor; c‐Yes, cellular Yes; CK2, casein kinase 2; DDK3, Dickkopf‐related protein 3; EGFR, epidermal growth factor receptor; ETS1, E26 transformation‐specific 1; EP4, prostaglandin E receptor 4; FOXM1, Forkhead box protein M1; HHEX, hematopoietically expressed homeobox; ISLR, immune‐signaling leucine‐rich repeat containing protein; JAK–STAT3, Janus kinase‐signal transducer and activator of transcription 3; KDM3A, lysine demethylase 3A; KRAS, kirsten rat sarcoma viral oncogene homolog; LATS1, large tumor suppressor homolog 1; Mcl1, myeloid cell leukemia sequence 1 apoptosis regulator; MEK, mitogen‐activated protein kinase kinase; MOB1, Mps one binder 1; MVA, mevalonic acid; NF‐κb, nuclear factor kappa B; PDGFRβ, platelet‐derived growth factor receptor β; PGE(2), prostaglandin E2; PP2A, protein phosphatase 2A; RASAL2, RAS protein activator like 2; REGγ, regulator of G protein signaling gamma; SDCBP, syndecan binding protein; SIK2, salt‐inducible kinase 2; SIRPγ, signal regulatory protein gamma; SLUG, Snail family zinc finger 2; SREBP2, sterol regulatory element binding protein‐2; TAZ, transcriptional coactivator with PDZ‐binding motif; TIAM1, T‐cell lymphoma invasion and metastasis inducing protein 1; USP47, ubiquitin‐specific peptidase 47; USP14, ubiquitin‐specific protease 14; VGLL4, vestigial like 4; YAP, Yes‐associated protein; YTHDF1, YTH domain family protein 1; ZIP4, Zrt‐ and Irt‐like protein 4; ZMYND8, zinc finger MYND‐type containing 8. The figure is created by Figdraw.

Pancreatic cancer, arising from the pancreatic ductal epithelium and acinar cells, is characterized by a poor prognosis and typically manifests in either the pancreatic head or tail [408, 409]. The development of pancreatic cancer involves a complex interplay of genetic factors, inflammation, pancreatic disorders, obesity, smoking, and diabetes [410]. Surgical resection stands out as the primary approach for managing early‐stage pancreatic cancer, particularly in cases of locally resectable tumors [411]. Chemotherapy and radiotherapy frequently complement surgical interventions, serving as adjuvant therapies administered pre‐ or postoperatively to diminish tumor burden, regulate disease progression, and enhance surgical outcomes [412, 413, 414]. In pancreatic cancer, direct YAP activation can drive tumor progression. Zinc transporter 4 boosts YAP1 expression by activating miR‐373 and suppressing LATS2 [415]. TEAD/YAP collaboration fosters pancreatic cancer advancement [416, 417, 418]. Carcinogenic Kirsten rat sarcoma viral oncogene homolog (KRAS) elevates JAK–STAT3 signaling via YAP1 and TAZ activation, promoting acinar to duct metaplasia and initiating pancreatic ductal adenocarcinoma progression [419]. Yap‐induced miR‐130a enhances YAP levels by targeting VGLL4 [420]. SDCBP impedes βTrCP (beta‐TRCP)‐mediated YAP1 degradation [421]. Ubiquitin‐specific peptidase 14 (USP14) facilitates K48‐linked TAZ deubiquitination, stabilizing TAZ [422]. Sul2 heightens PDGF receptor beta–YAP signaling, stimulating tumor growth and chemotherapy resistance [423]. lncRNA THAP9–AS1 amplifies YAP signaling [424]. Tamoxifen curbs myofibrogenic differentiation of pancreatic stellate cells in pancreatic cancer by deactivating YAP [425]. p53 boosts Ptpn14 expression, restraining YAP levels [426, 427] (Figure 6A).

### 3.12 Colorectal Cancer

Colorectal cancer, a malignancy arising in the epithelial cells of the colon or rectum, presents a multifaceted etiology involving genetic predisposition, dietary habits, lifestyle choices, and age [428, 429]. Genetic susceptibility, along with family history, are factors that can significantly heighten the risk of developing this condition. The typical manifestations of colorectal cancer include changes in bowel habits, abdominal discomfort, and rectal bleeding [430, 431]. Although surgical resection serves as the principal treatment approach for early‐stage colorectal cancer, the complex pathological characteristics of the disease present difficulties. Even with the incorporation of adjuvant therapies like postoperative radiotherapy and chemotherapy, attaining the best possible treatment outcomes still proves to be a formidable task [431, 432, 433, 434]. YAP1 activation correlates with colorectal cancer progression and poor prognosis [192, 435‐438]. In the Hippo pathway, regulator of G protein signaling gamma directly interacts with Lats1, facilitating its degradation and mutual activation of YAP and NF‐κB pathways [439]. The oncogenic transcription factor ETS proto‐oncogene 1 within stromal cells triggers the expression of immunoglobulin superfamily leucine‐rich repeat containing. This, in turn, suppresses Hippo signaling and activates YAP in epithelial cells. As a result, it promotes the regeneration of intestinal epithelial cells and contributes to tumorigenesis [440]. Ras GTPase activating protein like 2 (RASAL2) promotes colorectal cancer through the LATS2/YAP1 axis of the Hippo signaling pathway [441]. Circ‐YAP encodes YAP‐220AA, competitively binding to LATS1, leading to YAP dephosphorylation and nuclear translocation, promoting liver metastasis progression in colorectal cancer [442]. Beyond the Hippo pathway, the prostaglandin E (PGE) (2) signaling pathway enhances YAP1 expression and activity, upregulating cyclocyception 2 and prostaglandin E receptor 4 (EP4), stimulating colon cancer cell proliferation and colon tissue regeneration in colitis mice [443]. The interaction between tyrosine kinases c‐Yes and YAP fosters colon cancer recurrence [444]. Dickkopf‐related protein 3 orchestrates the coactivation of β‐catenin and YAP/TAZ [445]. Abhydrolase domain‐containing 5 deletion initiates the translocation of Dumpy homolog 30 to the nucleus, leading to the activation of SET domain‐containing 1A, which in turn facilitates the nuclear translocation of YAP. This process enhances the transcription of c‐Met, thereby promoting the stemness of colon cancer cells [446]. Casein kinase 2 facilitates hematopoietically expressed homeobox interaction with YAP/TEAD [447]. STAT3‐mediated signaling in colorectal cancer and melanoma endothelial cells boosts YAP/TAZ activity, driving angiogenesis [268]. Via the interaction between zinc finger MYND domain containing 8 and SREBP2, YAP elevates the expression of genes in the mevalonic acid pathway. This molecular process contributes to the progression of cholesterol‐related development in colorectal cancer [448]. VGLL4 targets the Tead4‐transcription factor 4 (TCF4) complex, disrupting TEAD4–TCF4 functional interaction [449]. Lysine demethylase 3A (KDM3A) [450], MAPK kinase [451] and USP47 [452] bolster YAP stability in colorectal cancer, while miR‐195‐5p inhibits YAP expression [453]. T lymphoma invasion and metastasis‐inducing protein 1 (TIAM1) hinders colorectal cancer progression by impeding YAP/TAZ interaction with TEADs [454] (Figure 6B).

### 3.13 Lung Cancer

Lung cancer is a malignant tumor originating from the mucous membranes or glands of the trachea and bronchus [455, 456, 457, 458]. The main causes of lung cancer include smoking, air pollution, and genetic predispositions, with smoking being the primary risk factor. Lung cancer is classified into non‐small cell lung cancer and small cell lung cancer [459]. Symptoms such as coughing, blood in sputum, or hemoptysis highlight the crucial need for early screening and diagnosis to improve treatment effectiveness. Common treatments for lung cancer include surgical resection, chemotherapy, radiation, and targeted therapy. Surgical excision is commonly used for early‐stage non‐small cell lung cancer, while chemotherapy and radiation are standard options for advanced cases or as adjunctive measures before and after surgery [460, 461, 462]. YAP expression in small cell lung cancer impacts disease cell phenotype, intertumor, and intratumor heterogeneity [265, 463‐466]. In lung adenocarcinoma cells, the interplay between EGFR and salt‐inducible kinase 2 within the Hippo pathway impedes the interaction between LATS1 and MST1. This process promotes the nuclear translocation of YAP and contributes to the development of resistance to tyrosine kinase inhibitors [467]. The regulatory axis of oxidative stress and CREB‐binding protein (CBP) controls MOB1–K11 acetylation, leading to the activation of LATS1 and initiation of the Hippo pathway. This activation inhibits the nuclear translocation of YAP/TAZ, thereby impeding the progression of non‐small cell lung cancer tumors [468]. SIRP gamma acts as a bridge between MST1 and PP2A, facilitating MST1 dephosphorylation and activating the Hippo/YAP pathway. This activation leads to the release of cytokines from tumor stem cells, triggers CD47 expression in lung adenocarcinoma cells, and ultimately inhibits the phagocytosis of tumor cells [469]. The N6‐methyladenosine (m(6)A) demethylase AlkB homolog 5 suppresses the growth and metastasis of non‐small cell lung cancer by decreasing YTH domain family protein 1‐mediated YAP expression and blocking miR‐107/LATS2‐mediated YAP activity [470]. Beyond the Hippo pathway, lung cancer cells survive EGFR inhibitor therapy through STAT3 and Src–YAP1 signaling activation [471]. Additionally, YAP/TEAD complex interacts with the EMT factor Snail family transcriptional repressor 2 (SLUG), which suppresses the proapoptotic factor Bcl‐2 modifying factor. This interaction restricts drug‐induced apoptosis in non‐small cell lung cancer cells [472]. The YAP/FOXM1 axis contributes to EGFR inhibitor resistance associated with EMT in lung cancer [19]. YAP suppresses squamous transdifferentiation of liver kinase B1‐deficient lung adenocarcinoma through zinc finger E‐box binding homeobox 2‐dependent DeltaNp63 [473]. YAP1 induces the expression of antiapoptotic factors myeloid cell leukemia sequence 1 (Mcl‐1) and B‐cell lymphoma‐extra large, enhancing lung cancer cell survival [474]. The YAP/TEAD pathway drives epigenome reprogramming and EMT to counteract lung cancer cell apoptosis [235] (Figure 6C).

### 3.14 Renal Cancer

**FIGURE 7:**
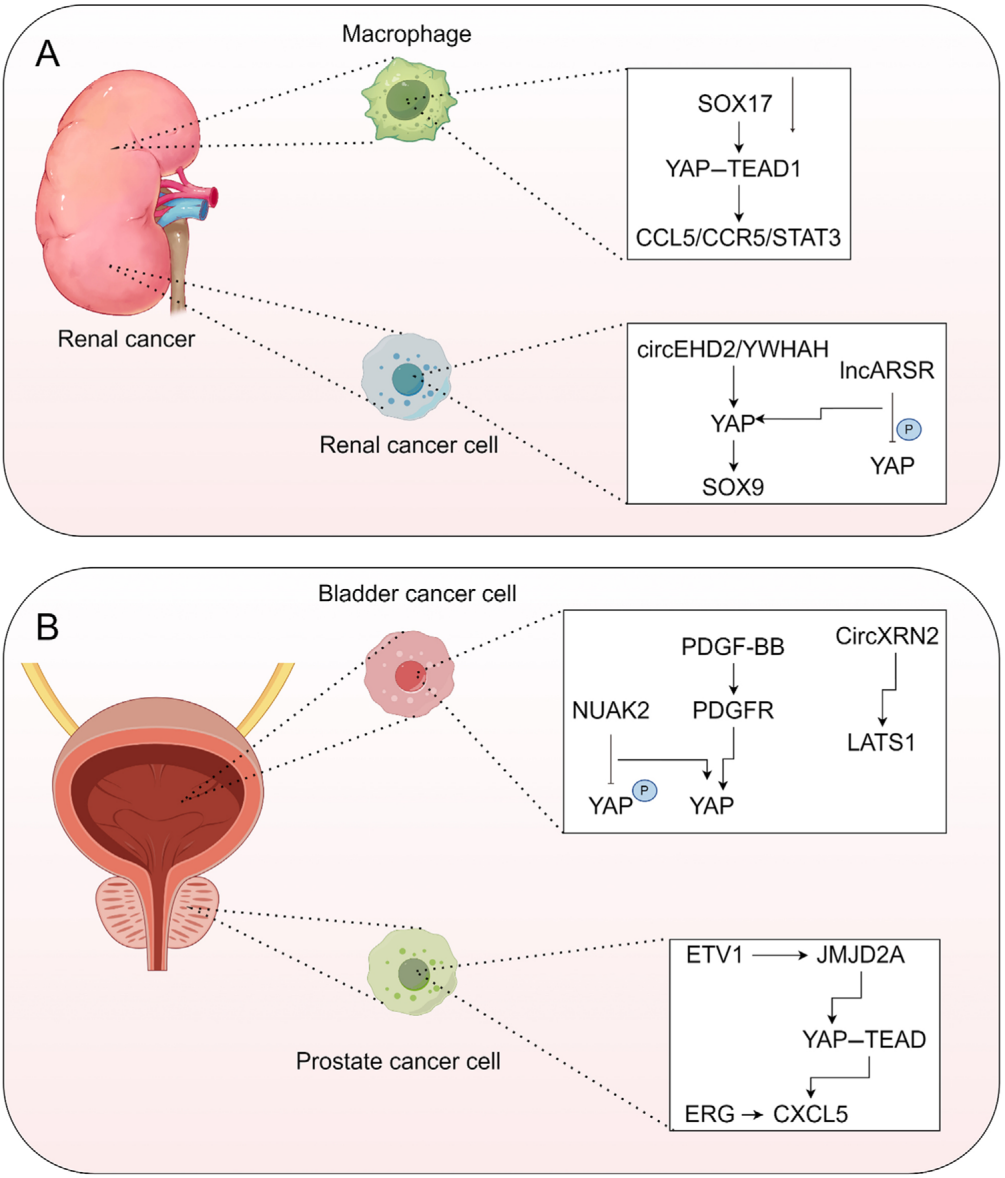
Molecular mechanism of YAP in renal cancer, bladder cancer, and prostate cancer. (A) Mechanism of YAP in renal cancer. (B) Mechanism of YAP in bladder cancer. (C) Mechanism of YAP in prostate cancer. CCL5, chemokine (C–C motif) ligand 5; CCR5, chemokine (C–C motif) receptor 5; CXCL5, chemokine (C–X–C motif) ligand 5; ERG, ETS‐related gene protein; ETV1, ETS variant transcription factor 1; JMJD2A, Jumonji domain‐containing protein 2A; LATS1, large tumor suppressor homolog 1; NUAK2, NUAK family, SNF1‐like kinase 2; PDGF‐BB, platelet‐derived growth factor subunit B dimer; PDGFR, platelet‐derived growth factor receptor; SOX9: sex‐determining region Y‐box 9; SOX17, Sex‐determining region Y‐Box 17; STAT3, signal transducer and activator of transcription 3; TEADs, TEA domain transcription factors; YAP, Yes‐associated protein; YWHAH, 14‐3‐3 protein eta. The figure is created by Figdraw.

Renal cancer, originating from the tubular epithelium of the kidney, includes different subtypes like clear cell carcinoma and papillary cell carcinoma. Among these, clear cell carcinoma is the most common subtype [475, 476]. The exact cause of kidney cancer remains unclear, but common risk factors include smoking, obesity, high blood pressure, and genetic predisposition. Symptoms of kidney cancer may include lower back pain, hematuria, and the presence of a kidney mass. Surgical interventions like partial or total nephrectomy are the main treatment options. Targeted therapies are crucial in managing kidney cancer by focusing on specific tumor growth factors or proteins, inhibiting tumor growth and spread [477, 478, 479]. YAP has emerged as a crucial gene involved in the development of kidney cancer. The interaction between lncARSR and YAP hinders YAP phosphorylation by Lats1 within the Hippo pathway. This process promotes YAP nuclear translocation, enhancing the tumorigenic activity of renal cell carcinoma cells in the kidney [480]. Studies indicate that partial activation of YAP in renal cancer can occur regardless of the Hippo pathway. In clear cell renal cell carcinoma, macrophages associated with the SOX17 (low)/YAP–TEAD1/CCL5/CCR5/STAT3 axis play a role in promoting metastasis and resistance to targeted drugs [481]. The circEHD2/tyrosine 3‐monooxygenase/tryptophan 5‐monooxygenase activation protein eta/YAP/SOX9 signaling pathway accelerates the growth of clear cell renal cell carcinoma [482, 483] (Figure 7A).

### 3.15 Bladder Cancer

Bladder cancer, a malignancy originating in the mucous membrane of the bladder, is commonly categorized into nonmuscular invasive and muscular invasive types [484, 485, 486]. Smoking stands out as a primary risk factor associated with this condition. Typical symptoms of bladder cancer encompass hematuria, frequent urination, urgency to urinate, lower back pain, and the presence of blood clots in the urine. Recognized as a multifaceted disease, early detection and intervention are pivotal in enhancing patient survival rates and overall well‐being [487, 488]. A comprehensive approach to managing bladder cancer involves a spectrum of treatments such as surgery, chemotherapy, radiotherapy, and emerging therapies [489, 490, 491]. The significance of YAP in bladder cancer has been highlighted, illuminating its role in the disease's development and possible therapeutic approaches. CircXRN2 inhibits bladder tumor advancement triggered by histone H3 lysine 18 lactation through the activation of the Hippo signaling pathway [492]. NUAK2 directly hinders LATS‐mediated YAP/TAZ phosphorylation, leading to YAP activation [493]. Upon YAP activation, YAP/TEAD1 enhances PDGF‐BB transcription, while PDGF‐BB, via its receptor PDGFR, stabilizes YAP and facilitates YAP nuclear translocation [296] (Figure 7B).

### 3.16 Prostate Cancer

Prostate cancer, an epithelial malignant tumor originating in the prostate, represents a prevalent malignancy within the male urinary system [494]. Characterized by a gradual progression, the exact cause of this condition remains incompletely understood, with factors such as age, family history, ethnicity, diet, and genetic components believed to play a significant role in its development [495, 496]. Symptoms of prostate cancer include frequent urination, urgency to urinate, and difficulties with urination. Common treatment options for prostate cancer include surgical resection, radiation therapy, chemotherapy, hormone therapy, and targeted therapy [497, 498]. The significant role of YAP in the development of prostate cancer emphasizes its crucial involvement in disease advancement and possible therapeutic strategies. In prostate cancer, the erythroblast transformation specific‐related gene binds to the chromatin region that is also occupied by TEAD/YAP [499]. ETS variant transcription factor 1 plays a role in recruiting YAP1 to the promoter region through JMJD2A, leading to changes in histone lysine methylation in human prostate cancer cell lines. This interaction highlights the complex regulatory network involved in prostate cancer progression [500]. The YAP–TEAD complex additionally promotes the upregulation of CXCL5 in cancer cells and facilitates the recruitment of polymorphic nuclear myeloid suppressor cells, thereby advancing the progression of prostate cancer [501] (Figure 7B).

## 4 Treatment

In the realms of inflammatory conditions and cancer treatment, a promising targeted therapy approach revolves around suppressing the elevated expression of YAP. Given its crucial role in the Hippo pathway, strategies that directly target YAP or disrupt the interaction between YAP/TAZ and TEADs are effective ways to regulate YAP expression. Additionally, future therapeutic strategies may involve modulating YAP activation and degradation by influencing the phosphorylation of MST1/2 and LATS1/2, presenting a hopeful pathway for intervention.

### 4.1 Targeting YAP/TAZ

Blocking YAP activation presents a promising avenue for treating inflammatory conditions and represents a novel strategy for future therapeutics. Triptolide has been demonstrated to inhibit the release of inflammatory mediators and mucus secretion by targeting YAP [65]. Prostaglandin E(2) has been identified as reducing fibroblast formation in asthma by inhibiting YAP [70]. The decrease in angiokinin‐like 2 and YAP1 inhibits airway smooth muscle cell proliferation in asthma [81]. Csrp2 inhibits the transition of airway smooth muscle cells to a synthetic/proliferative phenotype by suppressing YAP expression [82]. Lipid A4 disrupts Smad/YAP signaling, resulting in reduced proliferation and migration of airway smooth muscle cells in asthma [83]. Additionally, pirfenidone has been demonstrated to suppress pulmonary fibrosis formation post‐SARS‐CoV‐2 infection by targeting the YAP/TAZ pathway [112]. The vitamin D receptor promotes adaptive remodeling of bile ducts by upregulating YAP in bile duct cells, thereby mitigating cholestatic liver injury [502].

### 4.2 Targeting YAP–TEADs

VP, a small molecule compound, has shown effectiveness in disrupting the YAP–TEAD interaction and suppressing the Hippo pathway in different types of tumors and inflammatory disorders [154, 300, 503, 504]. It effectively suppresses YAP activity, prevents abnormal differentiation of nasal epithelial cells, and reduces the release of inflammatory factors [28, 42, 46]. ARID1A directly interacts with and suppresses the transcriptional coactivators YAP and TAZ, which drive cell proliferation. By competing with YAP/TAZ for binding to TEAD, ARID1A hinders adult cardiomyocyte regeneration [148]. MYC enhances AMPK and suppresses YAP/TAZ activity in breast tumors [331]. VGLL4 inhibits YAP activity by competitively binding to TEADs, thereby halting the progression of gastric cancer [349]. TIAM1 inhibits colorectal cancer progression by impeding the interaction of YAP/TAZ with TEADs [454].

### 4.3 Targeting MST1/2

Phosphorylation of MST1/2 can hinder the activation of YAP [1, 375]. In inflammatory conditions, phosphorylation of MST1/2 notably hinders YAP phosphorylation, leading to YAP activation and impeding its nuclear translocation, which in turn modulates the expression of inflammatory target genes [505, 506]. Similarly, phosphorylated MST1/2 suppresses YAP activation in tumor cells, leading to extracellular retention and degradation of YAP [321, 374, 507]. Targeting MST1/2 phosphorylation proves to be an effective strategy for YAP inhibition [508, 509]. NF2 exacerbates cardiac ischemia–reperfusion injury by activating MST1 and inhibiting YAP [150]. Adapalene can suppress YAP activation by triggering MST1/2, consequently relieving Staphylococcus aureus‐induced arthritis [510].

### 4.4 Targeting LATS1/2

Phosphorylation of LATS1/2 is pivotal for Hippo pathway activation. Mechanistically, LATS1/2 phosphorylation directly boosts YAP phosphorylation, leading to YAP degradation and the inhibition of YAP1 transcriptional regulation [1, 5]. In inflammatory diseases and tumors, LATS1/2 phosphorylation inhibits YAP activation, thereby modulating inflammation and tumor progression [320, 511, 512]. Glutamine is a key factor in maintaining cAMP/PKA activity and promoting LATS1/2 phosphorylation in tumor cells, ultimately repressing YAP activity [513]. Thus, promoting LATS1/2 phosphorylation emerges as a potential approach for YAP regulation.

## 5 Conclusion and Prospects

As a pivotal gene in growth and development, YAP plays a crucial role in promoting cell proliferation and differentiation, impacting the body's immune status. YAP activation, whether through the Hippo‐dependent or independent pathway, triggers inflammation and the release of inflammatory factors in respiratory, cardiovascular, and digestive inflammatory diseases. Additionally, it drives tumor cell proliferation and differentiation in various cancer types, while modulating the tumor immune microenvironment to enhance tumor metastasis and progression. Despite these insights, the potential for YAP intervention remains underutilized, necessitating extensive future research. Currently, investigations into YAP in inflammatory and oncological diseases are notably limited, with little focus on YAP‐specific treatments and a lack of reported effective clinical trials. Many studies are confined to cellular or animal models. Given the potential toxicities and challenges in controlling various inhibitors, further safety assessments and clinical trials are crucial to advance YAP or Hippo pathway treatments.

The overall inadequacy of translational research in inflammation and cancer highlights the pressing need for increased clinical drug studies to explore and validate potential therapies.

## Author Contributions

B. Z., S. S., J. D. and F. L. contributed to drafting and editing of the manuscript. All authors have read and approved the final manuscript.

## Ethics Statement

The authors have nothing to report.

## Conflicts of Interest

The authors declare no conflicts of interest.

## Supporting information



Supporting Information

## Data Availability

The authors have nothing to report.

## References

[1] J. H. Driskill and D. Pan , “Control of Stem Cell Renewal and Fate by YAP and TAZ,” Nature Reviews Molecular Cell Biology 24, no. 12 (2023): 895–911. 37626124 10.1038/s41580-023-00644-5

[2] H. L. Sladitschek‐Martens , A. Guarnieri , G. Brumana , et al., “YAP/TAZ Activity in Stromal Cells Prevents Ageing by Controlling cGAS‐STING,” Nature 607, no. 7920 (2022): 790–798. 35768505 10.1038/s41586-022-04924-6 PMC7613988

[3] J. Wu , A. M. Minikes , M. Gao , et al., “Intercellular Interaction Dictates Cancer Cell Ferroptosis via NF2‐YAP Signalling,” Nature 572, no. 7769 (2019): 402–406. 31341276 10.1038/s41586-019-1426-6 PMC6697195

[4] I. M. Moya , S. A. Castaldo , L. Van den Mooter , et al., “Peritumoral Activation of the Hippo Pathway Effectors YAP and TAZ Suppresses Liver Cancer in Mice,” Science 366, no. 6468 (2019): 1029–1034. 31754005 10.1126/science.aaw9886

[5] A. Dey , X. Varelas , and K. L. Guan , “Targeting the Hippo Pathway in Cancer, Fibrosis, Wound Healing and Regenerative Medicine,” Nature Reviews. Drug Discovery 19, no. 7 (2020): 480–494. 32555376 10.1038/s41573-020-0070-z PMC7880238

[6] T. Panciera , L. Azzolin , M. Cordenonsi , and S. Piccolo , “Mechanobiology of YAP and TAZ in Physiology and Disease,” Nature Reviews Molecular Cell Biology 18, no. 12 (2017): 758–770. 28951564 10.1038/nrm.2017.87 PMC6192510

[7] A. Totaro , T. Panciera , and S. Piccolo , “YAP/TAZ Upstream Signals and Downstream Responses,” Nature Cell Biology 20, no. 8 (2018): 888–899. 30050119 10.1038/s41556-018-0142-z PMC6186418

[8] V. Rausch and C. G. Hansen , “The Hippo Pathway, YAP/TAZ, and the Plasma Membrane,” Trends in Cell Biology 30, no. 1 (2020): 32–48. 31806419 10.1016/j.tcb.2019.10.005

[9] F. Zanconato , M. Cordenonsi , and S. Piccolo , “YAP and TAZ: A Signalling Hub of the Tumour Microenvironment,” Nature Reviews Cancer 19, no. 8 (2019): 454–464. 31270418 10.1038/s41568-019-0168-y

[10] L. S. Huang , Z. Hong , W. Wu , et al., “mtDNA Activates cGAS Signaling and Suppresses the YAP‐Mediated Endothelial Cell Proliferation Program to Promote Inflammatory Injury,” Immunity 52, no. 3 (2020): 475–486. e475. 32164878 10.1016/j.immuni.2020.02.002 PMC7266657

[11] L. Shen , P. Hu , Y. Zhang , et al., “Serine Metabolism Antagonizes Antiviral Innate Immunity by Preventing ATP6V0d2‐mediated YAP Lysosomal Degradation,” Cell Metabolism 33, no. 5 (2021): 971–987. e976. 33798471 10.1016/j.cmet.2021.03.006

[12] V. S. Meli , P. K. Veerasubramanian , T. L. Downing , W. Wang , and W. F. Liu , “Mechanosensation to Inflammation: Roles for YAP/TAZ in Innate Immune Cells,” Science Signaling 16, no. 783 (2023): eadc9656. 37130167 10.1126/scisignal.adc9656 PMC10625748

[13] V. S. Meli , H. Atcha , P. K. Veerasubramanian , et al., “YAP‐mediated Mechanotransduction Tunes the Macrophage Inflammatory Response,” Science Advances 6, no. 49 (2020): eabb8471. 33277245 10.1126/sciadv.abb8471 PMC7717914

[14] D. Chakravarti , B. Hu , X. Mao , et al., “Telomere Dysfunction Activates YAP1 to Drive Tissue Inflammation,” Nature Communications 11, no. 1 (2020): 4766. 10.1038/s41467-020-18420-w PMC7505960 32958778

[15] R. Caire , E. Audoux , M. Thomas , et al., “YAP Promotes Cell‐autonomous Immune Responses to Tackle Intracellular Staphylococcus aureus in Vitro,” Nature Communications 13, no. 1 (2022): 6995. 10.1038/s41467-022-34432-0 PMC9669043 36384856

[16] D. Wang , Y. Zhang , X. Xu , et al., “YAP Promotes the Activation of NLRP3 Inflammasome via Blocking K27‐linked Polyubiquitination of NLRP3,” Nature Communications 12, no. 1 (2021): 2674. 10.1038/s41467-021-22987-3 PMC8113592 33976226

[17] N. Van Sciver , M. Ohashi , N. P. Pauly , et al., “Hippo Signaling Effectors YAP and TAZ Induce Epstein‐Barr Virus (EBV) Lytic Reactivation Through TEADs in Epithelial Cells,” PLoS Pathogens 17, no. 8 (2021): e1009783. 34339458 10.1371/journal.ppat.1009783 PMC8360610

[18] Y. Liu , S. Zhuo , Y. Zhou , et al., “Yap‐Sox9 Signaling Determines Hepatocyte Plasticity and Lineage‐specific Hepatocarcinogenesis,” Journal of Hepatology 76, no. 3 (2022): 652–664. 34793870 10.1016/j.jhep.2021.11.010 PMC8858854

[19] M. B. Nilsson , H. Sun , J. Robichaux , et al., “A YAP/FOXM1 Axis Mediates EMT‐associated EGFR Inhibitor Resistance and Increased Expression of Spindle Assembly Checkpoint Components,” Science Translational Medicine 12, no. 559 (2020): eaaz4589. 32878980 10.1126/scitranslmed.aaz4589 PMC8269000

[20] F. Calvo , N. Ege , A. Grande‐Garcia , et al., “Mechanotransduction and YAP‐dependent Matrix Remodelling Is Required for the Generation and Maintenance of Cancer‐associated Fibroblasts,” Nature Cell Biology 15, no. 6 (2013): 637–646. 23708000 10.1038/ncb2756 PMC3836234

[21] A. Barettino , C. Gonzalez‐Gomez , P. Gonzalo , et al., “Endothelial YAP/TAZ Activation Promotes Atherosclerosis in a Mouse Model of Hutchinson‐Gilford progeria Syndrome,” Journal of Clinical Investigation 134, no. 22 (2024): e173448. 39352768 10.1172/JCI173448 PMC11563688

[22] C. G. Hansen , T. Moroishi , and K. L. Guan , “YAP and TAZ: A Nexus for Hippo Signaling and Beyond,” Trends in Cell Biology 25, no. 9 (2015): 499–513. 26045258 10.1016/j.tcb.2015.05.002 PMC4554827

[23] H. Qiu , J. Liu , Q. Wu , et al., “An in Vitro Study of the Impact of IL‐17A and IL‐22 on Ciliogenesis in Nasal Polyps Epithelium via the Hippo‐YAP Pathway,” Journal of Allergy and Clinical Immunology 154, no. 5 (2024): 1180–1194. 39033934 10.1016/j.jaci.2024.07.006

[24] I. T. Stancil , J. E. Michalski , C. E. Hennessy , et al., “Interleukin‐6‐dependent Epithelial Fluidization Initiates Fibrotic Lung Remodeling,” Science Translational Medicine 14, no. 654 (2022): eabo5254. 35857823 10.1126/scitranslmed.abo5254 PMC9981332

[25] K. Yang , J. Xu , M. Fan , et al., “Lactate Suppresses Macrophage Pro‐Inflammatory Response to LPS Stimulation by Inhibition of YAP and NF‐kappaB Activation via GPR81‐Mediated Signaling,” Frontiers in Immunology 11 (2020): 587913. 33123172 10.3389/fimmu.2020.587913 PMC7573489

[26] H. J. Choi , N. E. Kim , B. M. Kim , M. Seo , and J. H. Heo , “TNF‐alpha‐Induced YAP/TAZ Activity Mediates Leukocyte‐Endothelial Adhesion by Regulating VCAM1 Expression in Endothelial Cells,” International Journal of Molecular Sciences 19, no. 11 (2018): 3428. 30388809 10.3390/ijms19113428 PMC6274800

[27] Y. Lv , K. Kim , Y. Sheng , et al., “YAP Controls Endothelial Activation and Vascular Inflammation through TRAF6,” Circulation Research 123, no. 1 (2018): 43–56. 29794022 10.1161/CIRCRESAHA.118.313143 PMC6014930

[28] T. Yuan , R. Zheng , J. Liu , et al., “Role of Yes‐associated Protein in Interleukin‐13 Induced Nasal Remodeling of Chronic Rhinosinusitis With Nasal Polyps,” Allergy 76, no. 2 (2021): 600–604. 33301614 10.1111/all.14699

[29] R. A. Symons , F. Colella , F. L. Collins , et al., “Targeting the IL‐6‐Yap‐Snail Signalling Axis in Synovial Fibroblasts Ameliorates Inflammatory Arthritis,” Annals of the Rheumatic Diseases 81, no. 2 (2022): 214–224. 34844926 10.1136/annrheumdis-2021-220875 PMC8762018

[30] B. Zhong , J. Liu , H. H. Ong , et al., “Hypoxia‐reduced YAP Phosphorylation Enhances Expression of Mucin5AC in Nasal Epithelial Cells of Chronic Rhinosinusitis With Nasal Polyps,” Allergy (2024). 10.1111/all.16394 39535516

[31] N. S. Rachedi , Y. Tang , Y. Y. Tai , et al., “Dietary Intake and Glutamine‐serine Metabolism Control Pathologic Vascular Stiffness,” Cell Metabolism 36, no. 6 (2024): 1335–1350. e1338. 38701775 10.1016/j.cmet.2024.04.010 PMC11152997

[32] C. L. Guo , “Self‐Sustained Regulation or Self‐Perpetuating Dysregulation: ROS‐dependent HIF‐YAP‐Notch Signaling as a Double‐Edged Sword on Stem Cell Physiology and Tumorigenesis,” Frontiers in Cell and Developmental Biology 10 (2022): 862791. 35774228 10.3389/fcell.2022.862791 PMC9237464

[33] L. Wang , C. Wang , Z. Tao , et al., “Curcumin Derivative WZ35 Inhibits Tumor Cell Growth via ROS‐YAP‐JNK Signaling Pathway in Breast Cancer,” Journal of Experimental & Clinical Cancer Research 38, no. 1 (2019): 460. 31703744 10.1186/s13046-019-1424-4 PMC6842168

[34] C. Bachert , B. Marple , R. J. Schlosser , et al., “Adult Chronic Rhinosinusitis,” Nature Reviews Disease Primers 6, no. 1 (2020): 86. 10.1038/s41572-020-00218-1 33122665

[35] B. Zhong , J. Du , F. Liu , et al., “Hypoxia‐induced Factor‐1alpha Induces NLRP3 Expression by M1 Macrophages in Noneosinophilic Chronic Rhinosinusitis With Nasal Polyps,” Allergy 76, no. 2 (2021): 582–586. 32854144 10.1111/all.14571

[36] B. Zhong , J. J. Seah , F. Liu , L. Ba , J. Du , and Y. Wang , “The Role of Hypoxia in the Pathophysiology of Chronic Rhinosinusitis,” Allergy 77, no. 11 (2022): 3217–3232. 35603933 10.1111/all.15384

[37] B. Zhong , S. Sun , K. S. Tan , et al., “HIF‐1alpha Activates NLRP3 Inflammasome to Regulate Epithelial Differentiation in Chronic Rhinosinusitis,” Journal of Allergy and Clinical Immunology 152, no. 6 (2023): 1444–1459. 37777019 10.1016/j.jaci.2023.09.020

[38] S. He , J. Wu , D. Han , et al., “Differential Expression Profile of Plasma Exosomal microRNAs in Chronic Rhinosinusitis With Nasal Polyps,” Experimental Biology and Medicine (Maywood, N.J.) 247, no. 12 (2022): 1039–1046. 10.1177/15353702221090184 PMC9265519 35502556

[39] X. Liu , X. Tong , L. Zou , et al., “A Genome‐wide Association Study Reveals the Relationship Between human Genetic Variation and the Nasal Microbiome,” Communications Biology 7, no. 1 (2024): 139. 38291185 10.1038/s42003-024-05822-5 PMC10828421

[40] H. Qiu , J. Liu , Q. Wu , et al., “An in Vitro Study of the Impact of IL‐17A and IL‐22 on Ciliogenesis in Nasal Polyps Epithelium via the Hippo‐YAP Pathway,” Journal of Allergy and Clinical Immunology 154, no. 5 (2024): 1180–1194. 39033934 10.1016/j.jaci.2024.07.006

[41] H. Deng , Y. Sun , W. Wang , et al., “The Hippo Pathway Effector Yes‐associated Protein Promotes Epithelial Proliferation and Remodeling in Chronic Rhinosinusitis With Nasal Polyps,” Allergy 74, no. 4 (2019): 731–742. 30362580 10.1111/all.13647

[42] T. Yuan , R. Zheng , X. M. Zhou , et al., “Abnormal Expression of YAP Is Associated with Proliferation, Differentiation, Neutrophil Infiltration, and Adverse Outcome in Patients with Nasal Inverted Papilloma,” Frontiers in Cell and Developmental Biology 9 (2021): 625251. 33937228 10.3389/fcell.2021.625251 PMC8083899

[43] X. Jiang , L. Shu , Y. Liu , et al., “YES‐associated Protein‐regulated Smad7 Worsen Epithelial Barrier Injury of Chronic Sinusitis With Nasal Polyps,” Immunity, Inflammation and Disease 11, no. 6 (2023): e907. 37382248 10.1002/iid3.907 PMC10266168

[44] Y. Zhou , Y. Jiang , W. Peng , M. Li , H. Chen , and S. Chen , “The Diverse Roles of YAP in the Regulation of human Nasal Epithelial Remodeling,” Tissue & Cell 72 (2021): 101592. 34303282 10.1016/j.tice.2021.101592

[45] J. Zhan , H. Zhan , J. Zheng , X. Wei , and Y. Fu , “YAP1 expression in Nasal Polyps and Its Relationship With Epithelial Mesenchymal Transition,” American Journal of Translational Research 13, no. 6 (2021): 6568–6575. 34306398 PMC8290783

[46] H. Deng , M. Li , R. Zheng , et al., “YAP Promotes Cell Proliferation and Epithelium‐Derived Cytokine Expression via NF‐kappaB Pathway in Nasal Polyps,” Journal of Asthma and Allergy 14 (2021): 839–850. 34276219 10.2147/JAA.S315707 PMC8277454

[47] S. T. Holgate , S. Wenzel , D. S. Postma , S. T. Weiss , H. Renz , and P. D. Sly , “Asthma,” Nature Reviews Disease Primers 1, no. 1 (2015): 15025. 10.1038/nrdp.2015.25 PMC7096989 27189668

[48] B. N. Lambrecht , H. Hammad , and J. V. Fahy , “The Cytokines of Asthma,” Immunity 50, no. 4 (2019): 975–991. 30995510 10.1016/j.immuni.2019.03.018

[49] F. A. Vieira Braga , G. Kar , M. Berg , et al., “A Cellular Census of human Lungs Identifies Novel Cell States in Health and in Asthma,” Nature Medicine 25, no. 7 (2019): 1153–1163. 10.1038/s41591-019-0468-5 31209336

[50] R. L. Miller , M. H. Grayson , and K. Strothman , “Advances in Asthma: New Understandings of Asthma's Natural History, Risk Factors, Underlying Mechanisms, and Clinical Management,” Journal of Allergy and Clinical Immunology 148, no. 6 (2021): 1430–1441. 34655640 10.1016/j.jaci.2021.10.001

[51] R. Kaur and G. Chupp , “Phenotypes and Endotypes of Adult Asthma: Moving Toward Precision Medicine,” Journal of Allergy and Clinical Immunology 144, no. 1 (2019): 1–12. 31277742 10.1016/j.jaci.2019.05.031

[52] H. Xie , L. Wu , Z. Deng , Y. Huo , and Y. Cheng , “Emerging Roles of YAP/TAZ in Lung Physiology and Diseases,” Life Sciences 214 (2018): 176–183. 30385178 10.1016/j.lfs.2018.10.062

[53] W. Tang , M. Li , X. Yangzhong , et al., “Hippo Signaling Pathway and respiratory Diseases,” Cell Death Discovery 8, no. 1 (2022): 213. 35443749 10.1038/s41420-022-01020-6 PMC9021242

[54] L. E. Fodor , A. Gezsi , L. Ungvari , et al., “Investigation of the Possible Role of the Hippo/YAP1 Pathway in Asthma and Allergy,” Allergy, Asthma & Immunology Research 9, no. 3 (2017): 247–256. 10.4168/aair.2017.9.3.247 PMC5352576 28293931

[55] F. S. Yuliani , J. Y. Chen , W. H. Cheng , H. C. Wen , B. C. Chen , and C. H. Lin , “Thrombin Induces IL‐8/CXCL8 Expression by DCLK1‐dependent RhoA and YAP Activation in human Lung Epithelial Cells,” Journal of Biomedical Science 29, no. 1 (2022): 95. 36369000 10.1186/s12929-022-00877-0 PMC9650896

[56] H. Xiao , Q. N. Zhang , Q. X. Sun , L. D. Li , S. Y. Xu , and C. Q. Li , “Transcriptomic Analysis Reveals a Link between Hippo Signaling Pathway and Macrophages in Lungs of Mice With OVA‐Induced Allergic Asthma,” Journal of Inflammation Research 15 (2022): 423–437. 35082511 10.2147/JIR.S346505 PMC8784274

[57] E. K. Persson , K. Verstraete , I. Heyndrickx , et al., “Protein Crystallization Promotes Type 2 Immunity and Is Reversible by Antibody Treatment,” Science 364, no. 6442 (2019): eaaw4295. 31123109 10.1126/science.aaw4295

[58] X. Li , S. Guerra , J. G. Ledford , et al., “Low CC16 mRNA Expression Levels in Bronchial Epithelial Cells Are Associated With Asthma Severity,” American Journal of Respiratory and Critical Care Medicine 207, no. 4 (2023): 438–451. 36066606 10.1164/rccm.202206-1230OC PMC9940145

[59] R. Guidi , D. Xu , D. F. Choy , et al., “Steroid‐induced Fibroblast Growth Factors Drive an Epithelial‐mesenchymal Inflammatory Axis in Severe Asthma,” Science Translational Medicine 14, no. 641 (2022): eabl8146. 35442706 10.1126/scitranslmed.abl8146 PMC10301263

[60] L. Yuan , H. Liu , X. Du , et al., “Airway Epithelial ITGB4 Deficiency Induces Airway Remodeling in a Mouse Model,” Journal of Allergy and Clinical Immunology 151, no. 2 (2023): 431–446. e416. 36243221 10.1016/j.jaci.2022.09.032

[61] D. Wu , W. Jiang , C. Liu , et al., “CTNNAL1 participates in the Regulation of Mucus Overproduction in HDM‐induced Asthma Mouse Model Through the YAP‐ROCK2 Pathway,” Journal of Cellular and Molecular Medicine 26, no. 5 (2022): 1656–1671. 35092120 10.1111/jcmm.17206 PMC8899158

[62] M. L. Bu , M. H. Li , M. Feng , J. R. Wang , and L. Sun , “Poly(I:C) Exacerbates Airway Goblet Cell Hyperplasia and Lung Inflammation in HDM‐Exposed Balb/C Mice by YAP/FOXM1 Pathway,” International Archives of Allergy and Immunology 184, no. 7 (2023): 707–719. 36822170 10.1159/000529109

[63] I. T. Stancil , J. E. Michalski , D. Davis‐Hall , et al., “Pulmonary Fibrosis Distal Airway Epithelia Are Dynamically and Structurally Dysfunctional,” Nature Communications 12, no. 1 (2021): 4566. 10.1038/s41467-021-24853-8 PMC8316442 34315881

[64] J. J. Gokey , A. Sridharan , Y. Xu , et al., “Active Epithelial Hippo Signaling in Idiopathic Pulmonary Fibrosis,” JCI Insight 3, no. 6 (2018): e98738. 29563341 10.1172/jci.insight.98738 PMC5926907

[65] J. Gao and W. Wang , “Tripterine Alleviates Lipopolysaccharide‐induced Airway Epithelial Barrier Dysfunction Through Suppressing the Hippo Pathway,” RSC Advances 8, no. 69 (2018): 39696–39702. 35558034 10.1039/c8ra08614a PMC9091325

[66] L. B. Mostaco‐Guidolin , E. T. Osei , J. Ullah , et al., “Defective Fibrillar Collagen Organization by Fibroblasts Contributes to Airway Remodeling in Asthma,” American Journal of Respiratory and Critical Care Medicine 200, no. 4 (2019): 431–443. 30950644 10.1164/rccm.201810-1855OC

[67] X. O. Zhao , M. Lampinen , O. Rollman , C. P. Sommerhoff , A. Paivandy , and G. Pejler , “Mast Cell Chymase Affects the Functional Properties of Primary human Airway Fibroblasts: Implications for Asthma,” Journal of Allergy and Clinical Immunology 149, no. 2 (2022): 718–727. 34331992 10.1016/j.jaci.2021.07.020

[68] Q. Sun , L. Fang , M. Roth , et al., “Bronchial Thermoplasty Decreases Airway Remodelling by Blocking Epithelium‐derived Heat Shock Protein‐60 Secretion and Protein Arginine Methyltransferase‐1 in Fibroblasts,” European Respiratory Journal 54, no. 6 (2019): 1900300. 31467116 10.1183/13993003.00300-2019

[69] N. Miyashita , M. Horie , H. I. Suzuki , et al., “FOXL1 Regulates Lung Fibroblast Function via Multiple Mechanisms,” American Journal of Respiratory Cell and Molecular Biology 63, no. 6 (2020): 831–842. 32946266 10.1165/rcmb.2019-0396OC PMC8017595

[70] L. R. Teegala , R. Gudneppanavar , E. E. Sabu Kattuman , et al., “Prostaglandin E(2) Attenuates Lung Fibroblast Differentiation via Inactivation of Yes‐associated Protein Signaling,” Faseb Journal 37, no. 10 (2023): e23199. 37732601 10.1096/fj.202300745RR PMC11996057

[71] F. Wu , X. Li , M. Looso , et al., “Spurious Transcription Causing Innate Immune Responses Is Prevented by 5‐hydroxymethylcytosine,” Nature Genetics 55, no. 1 (2023): 100–111. 36539616 10.1038/s41588-022-01252-3 PMC9839451

[72] R. Saunders , H. Kaul , R. Berair , et al., “DP(2) Antagonism Reduces Airway Smooth Muscle Mass in Asthma by Decreasing Eosinophilia and Myofibroblast Recruitment,” Science Translational Medicine 11, no. 479 (2019): eaao6451. 30760581 10.1126/scitranslmed.aao6451

[73] C. Yon , D. A. Thompson , J. A. Jude , R. A. Panettieri Jr. , and D. Rastogi , “Crosstalk Between CD4(+) T Cells and Airway Smooth Muscle in Pediatric Obesity‐related Asthma,” American Journal of Respiratory and Critical Care Medicine 207, no. 4 (2023): 461–474. 36194662 10.1164/rccm.202205-0985OC PMC12042998

[74] S. V. Akkenepally , D. J. K. Yombo , S. Yerubandi , et al., “Interleukin 31 Receptor Alpha Promotes Smooth Muscle Cell Contraction and Airway Hyperresponsiveness in Asthma,” Nature Communications 14, no. 1 (2023): 8207. 10.1038/s41467-023-44040-1 PMC10713652 38081868

[75] J. Zhou , F. Xu , J. J. Yu , and W. Zhang , “YAP Is Up‐regulated in the Bronchial Airway Smooth Muscle of the Chronic Asthma Mouse Model,” International Journal of Clinical and Experimental Pathology 8, no. 9 (2015): 11132–11139. 26617833 PMC4637648

[76] J. Fu , M. Zheng , X. Zhang , et al., “Fibulin‐5 Promotes Airway Smooth Muscle Cell Proliferation and Migration via Modulating Hippo‐YAP/TAZ Pathway,” Biochemical and Biophysical Research Communications 493, no. 2 (2017): 985–991. 28942149 10.1016/j.bbrc.2017.09.105

[77] L. Liu , C. Zhai , Y. Pan , et al., “Sphingosine‐1‐phosphate Induces Airway Smooth Muscle Cell Proliferation, Migration, and Contraction by Modulating Hippo Signaling Effector YAP,” American Journal of Physiology. Lung Cellular and Molecular Physiology 315, no. 4 (2018): L609–L621. 29999407 10.1152/ajplung.00554.2017

[78] Z. Deng , H. Xie , W. Cheng , et al., “Dabigatran Ameliorates Airway Smooth Muscle Remodeling in Asthma by Modulating Yes‐associated Protein,” Journal of Cellular and Molecular Medicine 24, no. 14 (2020): 8179–8193. 32542982 10.1111/jcmm.15485 PMC7348141

[79] F. Wei and Y. Hao , “TRIP6 accelerates the Proliferation and Migration of Fetal Airway Smooth Muscle Cells by Enhancing YAP Activation,” International Immunopharmacology 82 (2020): 106366. 32151960 10.1016/j.intimp.2020.106366

[80] S. Zeng , J. Cui , Y. Zhang , Z. Zheng , J. Meng , and J. Du , “MicroRNA‐15b‐5p Inhibits Tumor Necrosis Factor Alpha‐induced Proliferation, Migration, and Extracellular Matrix Production of Airway Smooth Muscle Cells via Targeting Yes‐associated Protein 1,” Bioengineered 13, no. 3 (2022): 5396–5406. 35172671 10.1080/21655979.2022.2036890 PMC8974076

[81] P. Fang , W. J. Deng , N. Fan , et al., “AMOTL2 restrains Transforming Growth Factor‐beta1‐induced Proliferation and Extracellular Matrix Deposition of Airway Smooth Muscle Cells via the Down‐regulation of YAP1 Activation,” Environmental Toxicology 36, no. 11 (2021): 2225–2235. 34323359 10.1002/tox.23336

[82] H. Wang , Y. Zhang , B. Zhong , et al., “Cysteine and Glycine‐rich Protein 2 Retards Platelet‐derived Growth Factor‐BB‐evoked Phenotypic Transition of Airway Smooth Muscle Cells by Decreasing YAP/TAZ Activity,” Cell Biochemistry and Function 42, no. 1 (2024): e3896. 38081793 10.1002/cbf.3896

[83] Y. Zhao , X. Zhang , G. Wang , et al., “LXA4 inhibits TGF‐beta1‐induced Airway Smooth Muscle Cells Proliferation and Migration by Suppressing the Smad/YAP Pathway,” International Immunopharmacology 118 (2023): 110144. 37030120 10.1016/j.intimp.2023.110144

[84] A. H. Massoud , L. M. Charbonnier , D. Lopez , M. Pellegrini , W. Phipatanakul , and T. A. Chatila , “An Asthma‐associated IL4R Variant Exacerbates Airway Inflammation by Promoting Conversion of Regulatory T Cells to TH17‐Like Cells,” Nature Medicine 22, no. 9 (2016): 1013–1022. 10.1038/nm.4147 PMC5014738 27479084

[85] R. G. Luo , Y. F. Wu , H. W. Lu , et al., “Th2‐skewed Peripheral T Helper Cells Drives B Cells in Allergic Bronchopulmonary Aspergillosis,” European Respiratory Journal 63, no. 5 (2024): 2400386. 38514095 10.1183/13993003.00386-2024 PMC11096668

[86] Y. Xie , P. W. Abel , T. B. Casale , and Y. Tu , “T(H)17 Cells and Corticosteroid Insensitivity in Severe Asthma,” Journal of Allergy and Clinical Immunology 149, no. 2 (2022): 467–479. 34953791 10.1016/j.jaci.2021.12.769 PMC8821175

[87] J. Zhou , N. Zhang , W. Zhang , C. Lu , and F. Xu , “The YAP/HIF‐1alpha/miR‐182/EGR2 Axis Is Implicated in Asthma Severity Through the Control of Th17 Cell Differentiation,” Cell & Bioscience 11, no. 1 (2021): 84. 33980319 10.1186/s13578-021-00560-1 PMC8117288

[88] H. Grasemann and F. Ratjen , “Cystic Fibrosis,” New England Journal of Medicine 389, no. 18 (2023): 1693–1707. 37913507 10.1056/NEJMra2216474

[89] S. Y. Graeber and M. A. Mall , “The Future of Cystic Fibrosis Treatment: From Disease Mechanisms to Novel Therapeutic Approaches,” Lancet 402, no. 10408 (2023): 1185–1198. 37699417 10.1016/S0140-6736(23)01608-2

[90] J. Levring , D. S. Terry , Z. Kilic , G. Fitzgerald , S. C. Blanchard , and J. Chen , “CFTR Function, Pathology and Pharmacology at Single‐molecule Resolution,” Nature 616, no. 7957 (2023): 606–614. 36949202 10.1038/s41586-023-05854-7 PMC10115640

[91] M. Egan , T. Flotte , S. Afione , et al., “Defective Regulation of Outwardly Rectifying Cl‐ channels by Protein Kinase A Corrected by Insertion of CFTR,” Nature 358, no. 6387 (1992): 581–584. 1380129 10.1038/358581a0

[92] M. C. Quaresma , H. M. Botelho , I. Pankonien , et al., “Exploring YAP1‐centered Networks Linking Dysfunctional CFTR to Epithelial‐mesenchymal Transition,” Life Science Alliance 5, no. 9 (2022): e202101326. 35500936 10.26508/lsa.202101326 PMC9060002

[93] J. L. Simonin , C. Tomba , V. Mercier , et al., “Apical Dehydration Impairs the Cystic Fibrosis Airway Epithelium Barrier via a beta1‐integrin/YAP1 Pathway,” Life Science Alliance 7, no. 4 (2024): e202302449. 38336456 10.26508/lsa.202302449 PMC10858171

[94] S. K. Rajan , V. Cottin , R. Dhar , et al., “Progressive Pulmonary Fibrosis: An Expert Group Consensus Statement,” European Respiratory Journal 61, no. 3 (2023): 2103187. 36517177 10.1183/13993003.03187-2021 PMC10060665

[95] T. Koudstaal , M. Funke‐Chambour , M. Kreuter , P. L. Molyneaux , and M. S. Wijsenbeek , “Pulmonary Fibrosis: From Pathogenesis to Clinical Decision‐making,” Trends in Molecular Medicine 29, no. 12 (2023): 1076–1087. 37716906 10.1016/j.molmed.2023.08.010

[96] P. W. Noble , C. E. Barkauskas , and D. Jiang , “Pulmonary Fibrosis: Patterns and Perpetrators,” Journal of Clinical Investigation 122, no. 8 (2012): 2756–2762. 22850886 10.1172/JCI60323 PMC3408732

[97] L. Richeldi , H. R. Collard , and M. G. Jones , “Idiopathic Pulmonary Fibrosis,” Lancet 389, no. 10082 (2017): 1941–1952. 28365056 10.1016/S0140-6736(17)30866-8

[98] T. Zhang , X. He , L. Caldwell , et al., “NUAK1 promotes Organ Fibrosis via YAP and TGF‐beta/SMAD Signaling,” Science Translational Medicine 14, no. 637 (2022): eaaz4028. 35320001 10.1126/scitranslmed.aaz4028

[99] A. J. Haak , E. Kostallari , D. Sicard , et al., “Selective YAP/TAZ Inhibition in Fibroblasts via Dopamine Receptor D1 Agonism Reverses Fibrosis,” Science Translational Medicine 11, no. 516 (2019): eaau6296. 31666402 10.1126/scitranslmed.aau6296 PMC7066514

[100] K. Zmajkovicova , K. Menyhart , Y. Bauer , et al., “The Antifibrotic Activity of Prostacyclin Receptor Agonism Is Mediated Through Inhibition of YAP/TAZ,” American Journal of Respiratory Cell and Molecular Biology 60, no. 5 (2019): 578–591. 30537446 10.1165/rcmb.2018-0142OC

[101] P. J. Barnes , “New Anti‐inflammatory Targets for Chronic Obstructive Pulmonary Disease,” Nature Reviews Drug Discovery 12, no. 7 (2013): 543–559. 23977698 10.1038/nrd4025

[102] S. P. Bhatt , A. Agusti , M. Bafadhel , et al., “Phenotypes, Etiotypes, and Endotypes of Exacerbations of Chronic Obstructive Pulmonary Disease,” American Journal of Respiratory and Critical Care Medicine 208, no. 10 (2023): 1026–1041. 37560988 10.1164/rccm.202209-1748SO PMC10867924

[103] V. Laiman , Y. L. Lee , Y. W. Hou , et al., “Reduction of Emphysema Severity by Human Umbilical Cord‐Derived Mesenchymal Stem Cells in Mice,” International Journal of Molecular Sciences 23, no. 16 (2022): 8906. 36012176 10.3390/ijms23168906 PMC9408173

[104] F. J. Li , R. Surolia , H. Li , et al., “Low‐dose Cadmium Exposure Induces Peribronchiolar Fibrosis Through Site‐specific Phosphorylation of Vimentin,” American Journal of Physiology. Lung Cellular and Molecular Physiology 313, no. 1 (2017): L80–L91. 28450285 10.1152/ajplung.00087.2017 PMC5538875

[105] V. N. Petrova and C. A. Russell , “The Evolution of Seasonal Influenza Viruses,” Nature Reviews Microbiology 16, no. 1 (2018): 60. 10.1038/nrmicro.2017.146 29109554

[106] J. S. Long , B. Mistry , S. M. Haslam , and W. S. Barclay , “Host and Viral Determinants of Influenza A Virus Species Specificity,” Nature Reviews Microbiology 17, no. 2 (2019): 67–81. 30487536 10.1038/s41579-018-0115-z

[107] T. Flerlage , D. F. Boyd , V. Meliopoulos , P. G. Thomas , and S. Schultz‐Cherry , “Influenza Virus and SARS‐CoV‐2: Pathogenesis and Host Responses in the respiratory Tract,” Nature Reviews Microbiology 19, no. 7 (2021): 425–441. 33824495 10.1038/s41579-021-00542-7 PMC8023351

[108] T. M. Uyeki , D. S. Hui , M. Zambon , D. E. Wentworth , and A. S. Monto , “Influenza,” Lancet 400, no. 10353 (2022): 693–706. 36030813 10.1016/S0140-6736(22)00982-5 PMC9411419

[109] B. Hu , H. Guo , P. Zhou , and Z. L. Shi , “Characteristics of SARS‐CoV‐2 and COVID‐19,” Nature Reviews Microbiology 19, no. 3 (2021): 141–154. 33024307 10.1038/s41579-020-00459-7 PMC7537588

[110] A. Sette and S. Crotty , “Adaptive Immunity to SARS‐CoV‐2 and COVID‐19,” Cell 184, no. 4 (2021): 861–880. 33497610 10.1016/j.cell.2021.01.007 PMC7803150

[111] H. M. Al‐Kuraishy , A. I. Al‐Gareeb , H. M. Saad , and G. E. Batiha , “Hippo‐YAP Signaling and SARS‐CoV‐2 Infection: A New Mechanistic Pathway,” Cell Stress & Chaperones 28, no. 2 (2023): 121–123. 36752973 10.1007/s12192-023-01327-y PMC9907175

[112] H. M. Al‐Kuraishy , G. E. Batiha , H. Faidah , A. I. Al‐Gareeb , H. M. Saad , and J. Simal‐Gandara , “Pirfenidone and post‐Covid‐19 Pulmonary Fibrosis: Invoked Again for Realistic Goals,” Inflammopharmacology 30, no. 6 (2022): 2017–2026. 36044102 10.1007/s10787-022-01027-6 PMC9430017

[113] Q. Zhang , X. Zhang , X. Lei , et al., “Influenza A Virus NS1 Protein Hijacks YAP/TAZ to Suppress TLR3‐mediated Innate Immune Response,” PLoS Pathogens 18, no. 5 (2022): e1010505. 35503798 10.1371/journal.ppat.1010505 PMC9122210

[114] R. Li , J. Shao , Y. J. Jin , et al., “Endothelial FAT1 Inhibits Angiogenesis by Controlling YAP/TAZ Protein Degradation via E3 Ligase MIB2,” Nature Communications 14, no. 1 (2023): 1980. 10.1038/s41467-023-37671-x PMC10082778 37031213

[115] Y. Wang , G. Hu , F. Liu , et al., “Deletion of Yes‐associated Protein (YAP) Specifically in Cardiac and Vascular Smooth Muscle Cells Reveals a Crucial Role for YAP in Mouse Cardiovascular Development,” Circulation Research 114, no. 6 (2014): 957–965. 24478334 10.1161/CIRCRESAHA.114.303411 PMC4049286

[116] N. F. Ruopp and B. A. Cockrill , “Diagnosis and Treatment of Pulmonary Arterial Hypertension: A Review,” Jama 327, no. 14 (2022): 1379–1391. 35412560 10.1001/jama.2022.4402

[117] A. Mocumbi , M. Humbert , A. Saxena , et al., “Pulmonary Hypertension,” Nature Reviews Disease Primers 10, no. 1 (2024): 1. 10.1038/s41572-023-00486-7 38177157

[118] B. A. Maron and N. Galie , “Diagnosis, Treatment, and Clinical Management of Pulmonary Arterial Hypertension in the Contemporary Era: A Review,” JAMA Cardiology 1, no. 9 (2016): 1056–1065. 27851839 10.1001/jamacardio.2016.4471 PMC5177491

[119] K. Jandl , N. Radic , K. Zeder , G. Kovacs , and G. Kwapiszewska , “Pulmonary Vascular Fibrosis in Pulmonary Hypertension—The Role of the Extracellular Matrix as a Therapeutic Target,” Pharmacology & Therapeutics 247 (2023): 108438. 37210005 10.1016/j.pharmthera.2023.108438

[120] P. B. Dieffenbach , C. Mallarino Haeger , R. Rehman , et al., “A Novel Protective Role for Matrix Metalloproteinase‐8 in the Pulmonary Vasculature,” American Journal of Respiratory and Critical Care Medicine 204, no. 12 (2021): 1433–1451. 34550870 10.1164/rccm.202108-1863OC PMC8865706

[121] T. V. Kudryashova , D. A. Goncharov , A. Pena , et al., “HIPPO‐Integrin‐linked Kinase Cross‐Talk Controls Self‐Sustaining Proliferation and Survival in Pulmonary Hypertension,” American Journal of Respiratory and Critical Care Medicine 194, no. 7 (2016): 866–877. 27119551 10.1164/rccm.201510-2003OC PMC5074651

[122] J. Liu , J. Wang , Y. Liu , et al., “Liquid‐Liquid Phase Separation of DDR1 Counteracts the Hippo Pathway to Orchestrate Arterial Stiffening,” Circulation Research 132, no. 1 (2023): 87–105. 36475898 10.1161/CIRCRESAHA.122.322113

[123] T. Bertero , W. M. Oldham , K. A. Cottrill , et al., “Vascular Stiffness Mechanoactivates YAP/TAZ‐dependent Glutaminolysis to Drive Pulmonary Hypertension,” Journal of Clinical Investigation 126, no. 9 (2016): 3313–3335. 27548520 10.1172/JCI86387 PMC5004943

[124] P. Libby , “The Changing Landscape of Atherosclerosis,” Nature 592, no. 7855 (2021): 524–533. 33883728 10.1038/s41586-021-03392-8

[125] J. L. M. Bjorkegren and A. J. Lusis , “Atherosclerosis: Recent Developments,” Cell 185, no. 10 (2022): 1630–1645. 35504280 10.1016/j.cell.2022.04.004 PMC9119695

[126] P. Libby , J. E. Buring , L. Badimon , et al., “Atherosclerosis,” Nature Reviews Disease Primers 5, no. 1 (2019): 56. 10.1038/s41572-019-0106-z 31420554

[127] L. Wang , J. Y. Luo , B. Li , et al., “Integrin‐YAP/TAZ‐JNK Cascade Mediates Atheroprotective Effect of Unidirectional Shear Flow,” Nature 540, no. 7634 (2016): 579–582. 27926730 10.1038/nature20602

[128] K. C. Wang , Y. T. Yeh , P. Nguyen , et al., “Flow‐dependent YAP/TAZ Activities Regulate Endothelial Phenotypes and Atherosclerosis,” Proceeding of the National Academy of Sciences in the United States of America 113, no. 41 (2016): 11525–11530. 10.1073/pnas.1613121113 PMC5068257 27671657

[129] S. Xu , M. Koroleva , M. Yin , and Z. G. Jin , “Atheroprotective Laminar Flow Inhibits Hippo Pathway Effector YAP in Endothelial Cells,” Translational Research 176 (2016): 18–28. e12. 27295628 10.1016/j.trsl.2016.05.003 PMC5116386

[130] M. C. Jiang , H. Y. Ding , Y. H. Huang , et al., “Thioridazine Protects Against Disturbed Flow‐induced Atherosclerosis by Inhibiting RhoA/YAP‐mediated Endothelial Inflammation,” Acta Pharmacologica Sinica 44, no. 10 (2023): 1977–1988. 37217602 10.1038/s41401-023-01102-w PMC10545737

[131] J. Liu , C. Zhao , X. Xiao , et al., “Endothelial Discoidin Domain Receptor 1 Senses Flow to Modulate YAP Activation,” Nature Communications 14, no. 1 (2023): 6457. 10.1038/s41467-023-42341-z PMC10576099 37833282

[132] B. Li , J. He , H. Lv , et al., “c‐Abl Regulates YAPY357 Phosphorylation to Activate Endothelial Atherogenic Responses to Disturbed Flow,” Journal of Clinical Investigation 129, no. 3 (2019): 1167–1179. 30629551 10.1172/JCI122440 PMC6391101

[133] M. Jia , Q. Li , J. Guo , et al., “Deletion of BACH1 Attenuates Atherosclerosis by Reducing Endothelial Inflammation,” Circulation Research 130, no. 7 (2022): 1038–1055. 35196865 10.1161/CIRCRESAHA.121.319540

[134] M. D. Samsky , D. A. Morrow , A. G. Proudfoot , J. S. Hochman , H. Thiele , and S. V. Rao , “Cardiogenic Shock after Acute Myocardial Infarction: A Review,” Jama 326, no. 18 (2021): 1840–1850. 34751704 10.1001/jama.2021.18323 PMC9661446

[135] G. W. Reed , J. E. Rossi , and C. P. Cannon , “Acute Myocardial Infarction,” Lancet 389, no. 10065 (2017): 197–210. 27502078 10.1016/S0140-6736(16)30677-8

[136] J. L. Anderson and D. A. Morrow , “Acute Myocardial Infarction,” New England Journal of Medicine 376, no. 21 (2017): 2053–2064. 28538121 10.1056/NEJMra1606915

[137] J. Sadoshima , “YAP Promotes Infarct Resolution by Stimulating Intercellular Signaling,” Circulation Research 129, no. 8 (2021): 798–800. 34591659 10.1161/CIRCRESAHA.121.319981 PMC8514140

[138] D. P. Del Re , “Beyond the Cardiomyocyte: Consideration of HIPPO Pathway Cell‐Type Specificity,” Circulation Research 123, no. 1 (2018): 30–32. 29929975 10.1161/CIRCRESAHA.118.313383 PMC6053276

[139] G. Garoffolo , M. Casaburo , F. Amadeo , et al., “Reduction of Cardiac Fibrosis by Interference with YAP‐Dependent Transactivation,” Circulation Research 131, no. 3 (2022): 239–257. 35770662 10.1161/CIRCRESAHA.121.319373

[140] Z. Lin , A. von Gise , P. Zhou , et al., “Cardiac‐specific YAP Activation Improves Cardiac Function and Survival in an Experimental Murine MI Model,” Circulation Research 115, no. 3 (2014): 354–363. 24833660 10.1161/CIRCRESAHA.115.303632 PMC4104149

[141] Y. Morikawa , T. Heallen , J. Leach , Y. Xiao , and J. F. Martin , “Dystrophin‐glycoprotein Complex Sequesters Yap to Inhibit Cardiomyocyte Proliferation,” Nature 547, no. 7662 (2017): 227–231. 28581498 10.1038/nature22979 PMC5528853

[142] M. M. Mia , D. M. Cibi , S. A. B. Abdul Ghani , et al., “YAP/TAZ Deficiency Reprograms Macrophage Phenotype and Improves Infarct Healing and Cardiac Function After Myocardial Infarction,” PLoS Biology 18, no. 12 (2020): e3000941. 33264286 10.1371/journal.pbio.3000941 PMC7735680

[143] A. Aharonov , A. Shakked , K. B. Umansky , et al., “ERBB2 drives YAP Activation and EMT‐Like Processes During Cardiac Regeneration,” Nature Cell Biology 22, no. 11 (2020): 1346–1356. 33046882 10.1038/s41556-020-00588-4

[144] D. Zhang , J. Ning , T. Ramprasath , et al., “Kynurenine Promotes Neonatal Heart Regeneration by Stimulating Cardiomyocyte Proliferation and Cardiac Angiogenesis,” Nature Communications 13, no. 1 (2022): 6371. 10.1038/s41467-022-33734-7 PMC9606021 36289221

[145] X. Wang , T. Ha , L. Liu , et al., “TLR3 Mediates Repair and Regeneration of Damaged Neonatal Heart Through Glycolysis Dependent YAP1 Regulated miR‐152 Expression,” Cell Death and Differentiation 25, no. 5 (2018): 966–982. 29358670 10.1038/s41418-017-0036-9 PMC5943401

[146] J. Li , E. Gao , A. Vite , et al., “Alpha‐catenins Control Cardiomyocyte Proliferation by Regulating Yap Activity,” Circulation Research 116, no. 1 (2015): 70–79. 25305307 10.1161/CIRCRESAHA.116.304472 PMC4282606

[147] Y. Li , J. Feng , S. Song , et al., “gp130 Controls Cardiomyocyte Proliferation and Heart Regeneration,” Circulation 142, no. 10 (2020): 967–982. 32600062 10.1161/CIRCULATIONAHA.119.044484

[148] C. J. Boogerd , I. Perini , E. Kyriakopoulou , et al., “Cardiomyocyte Proliferation Is Suppressed by ARID1A‐mediated YAP Inhibition During Cardiac Maturation,” Nature Communications 14, no. 1 (2023): 4716. 10.1038/s41467-023-40203-2 PMC10404286 37543677

[149] V. Ramjee , D. Li , L. J. Manderfield , et al., “Epicardial YAP/TAZ Orchestrate an Immunosuppressive Response Following Myocardial Infarction,” Journal of Clinical Investigation 127, no. 3 (2017): 899–911. 28165342 10.1172/JCI88759 PMC5330722

[150] T. Matsuda , P. Zhai , S. Sciarretta , et al., “NF2 Activates Hippo Signaling and Promotes Ischemia/Reperfusion Injury in the Heart,” Circulation Research 119, no. 5 (2016): 596–606. 27402866 10.1161/CIRCRESAHA.116.308586 PMC4992450

[151] D. Shao , P. Zhai , D. P. Del Re , et al., “A Functional Interaction Between Hippo‐YAP Signalling and FoxO1 Mediates the Oxidative Stress Response,” Nature Communications 5 (2014): 3315. 10.1038/ncomms4315 PMC3962829 24525530

[152] Y. Yang , D. P. Del Re , N. Nakano , et al., “miR‐206 Mediates YAP‐Induced Cardiac Hypertrophy and Survival,” Circulation Research 117, no. 10 (2015): 891–904. 26333362 10.1161/CIRCRESAHA.115.306624 PMC4747867

[153] M. Qiu , W. Yan , and M. Liu , “YAP Facilitates NEDD4L‐Mediated Ubiquitination and Degradation of ACSL4 to Alleviate Ferroptosis in Myocardial Ischemia‐Reperfusion Injury,” Canadian Journal of Cardiology 39, no. 11 (2023): 1712–1727. 37541340 10.1016/j.cjca.2023.07.030

[154] G. Zhong , S. Su , J. Li , et al., “Activation of Piezo1 Promotes Osteogenic Differentiation of Aortic Valve Interstitial Cell Through YAP‐dependent Glutaminolysis,” Science Advances 9, no. 22 (2023): eadg0478. 37267365 10.1126/sciadv.adg0478 PMC10413650

[155] Z. D. Pang , X. Sun , R. Y. Bai , et al., “YAP‐galectin‐3 Signaling Mediates Endothelial Dysfunction in Angiotensin II‐induced Hypertension in Mice,” Cellular and Molecular Life Sciences 80, no. 2 (2023): 38. 36629913 10.1007/s00018-022-04623-5 PMC11072047

[156] S. Ikeda , W. Mizushima , S. Sciarretta , et al., “Hippo Deficiency Leads to Cardiac Dysfunction Accompanied by Cardiomyocyte Dedifferentiation during Pressure Overload,” Circulation Research 124, no. 2 (2019): 292–305. 30582455 10.1161/CIRCRESAHA.118.314048 PMC6645688

[157] J. Wang , S. Liu , T. Heallen , and J. F. Martin , “The Hippo Pathway in the Heart: Pivotal Roles in Development, Disease, and Regeneration,” Nature Reviews Cardiology 15, no. 11 (2018): 672–684. 30111784 10.1038/s41569-018-0063-3

[158] F. Biagioni , O. Croci , S. Sberna , et al., “Decoding YAP Dependent Transcription in the Liver,” Nucleic Acids Research 50, no. 14 (2022): 7959–7971. 35871292 10.1093/nar/gkac624 PMC9371928

[159] W. Li , L. Yang , Q. He , et al., “A Homeostatic Arid1a‐Dependent Permissive Chromatin State Licenses Hepatocyte Responsiveness to Liver‐Injury‐Associated YAP Signaling,” Cell Stem Cell 25, no. 1 (2019): 54–68. e55. 31271748 10.1016/j.stem.2019.06.008

[160] K. Liu , L. Wehling , S. Wan , et al., “Dynamic YAP Expression in the Non‐parenchymal Liver Cell Compartment Controls Heterologous Cell Communication,” Cellular and Molecular Life Sciences 81, no. 1 (2024): 115. 38436764 10.1007/s00018-024-05126-1 PMC10912141

[161] H. Hirao , K. Nakamura , and J. W. Kupiec‐Weglinski , “Liver Ischaemia‐reperfusion Injury: A New Understanding of the Role of Innate Immunity,” Nature Reviews Gastroenterology & Hepatology 19, no. 4 (2022): 239–256. 34837066 10.1038/s41575-021-00549-8

[162] C. Peralta , M. B. Jimenez‐Castro , and J. Gracia‐Sancho , “Hepatic Ischemia and Reperfusion Injury: Effects on the Liver Sinusoidal Milieu,” Journal of Hepatology 59, no. 5 (2013): 1094–1106. 23811302 10.1016/j.jhep.2013.06.017

[163] D. A. Parks and D. N. Granger , “Ischemia‐reperfusion Injury: A Radical View,” Hepatology 8, no. 3 (1988): 680–682. 3286463 10.1002/hep.1840080341

[164] J. Zhou , M. Hu , M. He , et al., “TNFAIP3 Interacting Protein 3 Is an Activator of Hippo‐YAP Signaling Protecting against Hepatic Ischemia/Reperfusion Injury,” Hepatology 74, no. 4 (2021): 2133–2153. 34133792 10.1002/hep.32015

[165] J. Rao , F. Cheng , H. Zhou , et al., “Nogo‐B Is a Key Mediator of Hepatic Ischemia and Reperfusion Injury,” Redox Biology 37 (2020): 101745. 33099216 10.1016/j.redox.2020.101745 PMC7582106

[166] Y. Liu , T. Lu , C. Zhang , et al., “Activation of YAP Attenuates Hepatic Damage and Fibrosis in Liver Ischemia‐reperfusion Injury,” Journal of Hepatology 71, no. 4 (2019): 719–730. 31201834 10.1016/j.jhep.2019.05.029 PMC6773499

[167] S. Zhang , Z. Sun , Z. Chen , et al., “Endothelial YAP/TEAD1‐CXCL17 Signaling Recruits Myeloid‐derived Suppressor Cells Against Liver Ischemia‐reperfusion Injury,” Hepatology 81, no. 3 (2024): 888–902. 10.1097/HEP.0000000000000773 PMC11825485 38407233

[168] Z. Yuan , L. Ye , X. Feng , et al., “YAP‐Dependent Induction of CD47‐Enriched Extracellular Vesicles Inhibits Dendritic Cell Activation and Ameliorates Hepatic Ischemia‐Reperfusion Injury,” Oxidative Medicine and Cellular Longevity 2021 (2021): 6617345. 34239692 10.1155/2021/6617345 PMC8241504

[169] W. J. Jeng , G. V. Papatheodoridis , A. S. F. Lok , and B. Hepatitis , Lancet 401, no. 10381 (2023): 1039–1052. 36774930 10.1016/S0140-6736(22)01468-4

[170] R. S. Khan , P. F. Lalor , M. Thursz , and P. N. Newsome , “The Role of Neutrophils in Alcohol‐related hepatitis,” Journal of Hepatology 79, no. 4 (2023): 1037–1048. 37290590 10.1016/j.jhep.2023.05.017

[171] G. Mieli‐Vergani and D. Vergani , “Autoimmune hepatitis,” Nature Reviews Gastroenterology & Hepatology 8, no. 6 (2011): 320–329. 21537351 10.1038/nrgastro.2011.69

[172] A. Vogel , T. Meyer , G. Sapisochin , R. Salem , and A. Saborowski , “Hepatocellular Carcinoma,” Lancet 400, no. 10360 (2022): 1345–1362. 36084663 10.1016/S0140-6736(22)01200-4

[173] J. D. Yang , P. Hainaut , G. J. Gores , A. Amadou , A. Plymoth , and L. R. Roberts , “A Global View of Hepatocellular Carcinoma: Trends, Risk, Prevention and Management,” Nature Reviews Gastroenterology & Hepatology 16, no. 10 (2019): 589–604. 31439937 10.1038/s41575-019-0186-y PMC6813818

[174] M. Bou Saleh , A. Louvet , L. C. Ntandja‐Wandji , et al., “Loss of Hepatocyte Identity Following Aberrant YAP Activation: A Key Mechanism in Alcoholic hepatitis,” Journal of Hepatology 75, no. 4 (2021): 912–923. 34129887 10.1016/j.jhep.2021.05.041 PMC11868489

[175] P. Kusumanchi , T. Liang , T. Zhang , et al., “Stress‐Responsive Gene FK506‐Binding Protein 51 Mediates Alcohol‐Induced Liver Injury through the Hippo Pathway and Chemokine (C‐X‐C Motif) Ligand 1 Signaling,” Hepatology 74, no. 3 (2021): 1234–1250. 33710653 10.1002/hep.31800 PMC8435051

[176] Y. Hao , D. Feng , H. Ye , and W. Liao , “Nobiletin Alleviated Epithelial‐Mesenchymal Transition of Hepatocytes in Liver Fibrosis Based on Autophagy‐Hippo/YAP Pathway,” Molecular Nutrition & Food Research 68, no. 3 (2024): e2300529. 38044268 10.1002/mnfr.202300529

[177] Y. Aylon , A. Gershoni , R. Rotkopf , et al., “The LATS2 Tumor Suppressor Inhibits SREBP and Suppresses Hepatic Cholesterol Accumulation,” Genes & Development 30, no. 7 (2016): 786–797. 27013235 10.1101/gad.274167.115 PMC4826395

[178] C. Priest , R. T. Nagari , L. Bideyan , et al., “Brap Regulates Liver Morphology and Hepatocyte Turnover via Modulation of the Hippo Pathway,” Proceeding of the National Academy of Sciences in the United States of America 119, no. 18 (2022): e2201859119. 10.1073/pnas.2201859119 PMC9171358 35476518

[179] M. Mooring , B. H. Fowl , S. Z. C. Lum , et al., “Hepatocyte Stress Increases Expression of Yes‐Associated Protein and Transcriptional Coactivator with PDZ‐Binding Motif in Hepatocytes to Promote Parenchymal Inflammation and Fibrosis,” Hepatology 71, no. 5 (2020): 1813–1830. 31505040 10.1002/hep.30928 PMC7062580

[180] C. Li , Y. Jin , S. Wei , et al., “Hippo Signaling Controls NLR Family Pyrin Domain Containing 3 Activation and Governs Immunoregulation of Mesenchymal Stem Cells in Mouse Liver Injury,” Hepatology 70, no. 5 (2019): 1714–1731. 31063235 10.1002/hep.30700 PMC6819196

[181] T. Yang , X. Qu , X. Wang , et al., “The Macrophage STING‐YAP Axis Controls Hepatic Steatosis by Promoting the Autophagic Degradation of Lipid Droplets,” Hepatology 80, no. 5 (2023): 1169–1183. 37870294 10.1097/HEP.0000000000000638 PMC11035483

[182] S. Alsamman , S. A. Christenson , A. Yu , et al., “Targeting Acid Ceramidase Inhibits YAP/TAZ Signaling to Reduce Fibrosis in Mice,” Science Translational Medicine 12, no. 557 (2020): eaay8798. 32817366 10.1126/scitranslmed.aay8798 PMC7976849

[183] I. Mederacke , A. Filliol , S. Affo , et al., “The Purinergic P2Y14 Receptor Links Hepatocyte Death to Hepatic Stellate Cell Activation and Fibrogenesis in the Liver,” Science Translational Medicine 14, no. 639 (2022): eabe5795. 35385339 10.1126/scitranslmed.abe5795 PMC9436006

[184] K. Song , H. Kwon , C. Han , et al., “Yes‐Associated Protein in Kupffer Cells Enhances the Production of Proinflammatory Cytokines and Promotes the Development of Nonalcoholic Steatohepatitis,” Hepatology 72, no. 1 (2020): 72–87. 31610032 10.1002/hep.30990 PMC7153981

[185] J. Torres , S. Mehandru , and J. F. Colombel , “Peyrin‐Biroulet L,” Lancet 389, no. 10080 (2017): 1741–1755. 27914655 10.1016/S0140-6736(16)31711-1

[186] G. Roda , S. Chien Ng , P. G. Kotze , et al., “Crohn's Disease,” Nature Reviews Disease Primers 6, no. 1 (2020): 22. 10.1038/s41572-020-0156-2 32242028

[187] C. Le Berre , S. Honap , and L. Peyrin‐Biroulet , “Ulcerative Colitis,” Lancet 402, no. 10401 (2023): 571–584. 37573077 10.1016/S0140-6736(23)00966-2

[188] S. Thorsteinsdottir , T. Gudjonsson , O. H. Nielsen , B. Vainer , and J. B. Seidelin , “Pathogenesis and Biomarkers of Carcinogenesis in Ulcerative Colitis,” Nature Reviews Gastroenterology & Hepatology 8, no. 7 (2011): 395–404. 21647200 10.1038/nrgastro.2011.96

[189] L. Liu , Y. Wang , S. Yu , et al., “Transforming Growth Factor Beta Promotes Inflammation and Tumorigenesis in Smad4‐Deficient Intestinal Epithelium in a YAP‐Dependent Manner,” Advanced Science (Weinh) 10, no. 23 (2023): e2300708. 10.1002/advs.202300708 PMC10427365 37261975

[190] K. Taniguchi , L. W. Wu , S. I. Grivennikov , et al., “A gp130‐Src‐YAP Module Links Inflammation to Epithelial Regeneration,” Nature 519, no. 7541 (2015): 57–62. 25731159 10.1038/nature14228 PMC4447318

[191] H. Zhu , J. Lu , M. Fu , et al., “YAP Represses Intestinal Inflammation Through Epigenetic Silencing of JMJD3,” Clinical Epigenetics 16, no. 1 (2024): 14. 38245781 10.1186/s13148-024-01626-w PMC10800074

[192] Y. Liu , Y. Ji , R. Jiang , et al., “Reduced Smooth Muscle‐fibroblasts Transformation Potentially Decreases Intestinal Wound Healing and Colitis‐associated Cancer in Ageing Mice,” Signal Transduction and Targeted Therapy 8, no. 1 (2023): 294. 37553378 10.1038/s41392-023-01554-w PMC10409725

[193] M. Yu , Y. Luo , Z. Cong , Y. Mu , Y. Qiu , and M. Zhong , “MicroRNA‐590‐5p Inhibits Intestinal Inflammation by Targeting YAP,” Journal of Crohn's and Colitis 12, no. 8 (2018): 993–1004. 10.1093/ecco-jcc/jjy046 29912317

[194] F. Deng , Z. Wu , F. Zou , S. Wang , and X. Wang , “The Hippo‐YAP/TAZ Signaling Pathway in Intestinal Self‐Renewal and Regeneration after Injury,” Frontiers in Cell and Developmental Biology 10 (2022): 894737. 35927987 10.3389/fcell.2022.894737 PMC9343807

[195] D. Chakravarti , R. Lee , A. S. Multani , et al., “Telomere Dysfunction Instigates Inflammation in Inflammatory Bowel Disease,” Proceeding of the National Academy of Sciences in the United States of America 118, no. 29 (2021): e2024853118. 10.1073/pnas.2024853118 PMC8307535 34253611

[196] S. He , P. Lei , W. Kang , et al., “Stiffness Restricts the Stemness of the Intestinal Stem Cells and Skews Their Differentiation toward Goblet Cells,” Gastroenterology 164, no. 7 (2023): 1137–1151. e1115. 36871599 10.1053/j.gastro.2023.02.030 PMC10200762

[197] X. Zhou , W. Li , S. Wang , et al., “YAP Aggravates Inflammatory Bowel Disease by Regulating M1/M2 Macrophage Polarization and Gut Microbial Homeostasis,” Cell reports 27, no. 4 (2019): 1176–1189. e1175. 31018132 10.1016/j.celrep.2019.03.028

[198] W. Ou , W. Xu , F. Liu , et al., “Increased Expression of Yes‐associated Protein/YAP and Transcriptional Coactivator With PDZ‐binding Motif/TAZ Activates Intestinal Fibroblasts to Promote Intestinal Obstruction in Crohn's disease,” EBioMedicine 69 (2021): 103452. 34186485 10.1016/j.ebiom.2021.103452 PMC8243379

[199] P. Zhang , T. Wang , D. Zhang , et al., “Exploration of MST1‐Mediated Secondary Brain Injury Induced by Intracerebral Hemorrhage in Rats via Hippo Signaling Pathway,” Translational Stroke Research 10, no. 6 (2019): 729–743. 30941717 10.1007/s12975-019-00702-1

[200] Y. Yang , J. Ren , Y. Sun , et al., “A connexin43/YAP Axis Regulates Astroglial‐mesenchymal Transition in Hemoglobin Induced Astrocyte Activation,” Cell Death and Differentiation 25, no. 10 (2018): 1870–1884. 29880858 10.1038/s41418-018-0137-0 PMC6180064

[201] Z. Huang , Y. Wang , G. Hu , J. Zhou , L. Mei , and W. C. Xiong , “YAP Is a Critical Inducer of SOCS3, Preventing Reactive Astrogliosis,” Cerebral Cortex 26, no. 5 (2016): 2299–2310. 26679195 10.1093/cercor/bhv292 PMC4830299

[202] W. Li , P. Xu , L. Kong , et al., “Elabela‐APJ Axis Mediates Angiogenesis via YAP/TAZ Pathway in Cerebral Ischemia/Reperfusion Injury,” Translational Research 257 (2023): 78–92. 36813109 10.1016/j.trsl.2023.02.002

[203] A. Vivante , “Genetics of Chronic Kidney Disease,” New England Journal of Medicine 391, no. 7 (2024): 627–639. 39141855 10.1056/NEJMra2308577

[204] P. Drawz and M. Rahman , “Chronic Kidney Disease,” Annals of Internal Medicine 162, no. 11 (2015): ITC1–16.10.7326/AITC20150602026030647

[205] J. A. Kellum , P. Romagnani , G. Ashuntantang , C. Ronco , A. Zarbock , and H. J. Anders , “Acute Kidney Injury,” Nature Reviews Disease Primers 7, no. 1 (2021): 52. 10.1038/s41572-021-00284-z 34267223

[206] C. Ronco , R. Bellomo , and J. A. Kellum , “Acute Kidney Injury,” Lancet 394, no. 10212 (2019): 1949–1964. 31777389 10.1016/S0140-6736(19)32563-2

[207] T. T. Wang , L. L. Wu , J. Wu , et al., “14‐3‐3zeta inhibits Maladaptive Repair in Renal Tubules by Regulating YAP and Reduces Renal Interstitial Fibrosis,” Acta Pharmacologica Sinica 44, no. 2 (2023): 381–392. 35840657 10.1038/s41401-022-00946-y PMC9889378

[208] D. Xu , P. P. Chen , P. Q. Zheng , et al., “KLF4 initiates Sustained YAP Activation to Promote Renal Fibrosis in Mice After Ischemia‐reperfusion Kidney Injury,” Acta Pharmacologica Sinica 42, no. 3 (2021): 436–450. 32647339 10.1038/s41401-020-0463-x PMC8027004

[209] J. Chen , H. You , Y. Li , Y. Xu , Q. He , and R. C. Harris , “EGF Receptor‐Dependent YAP Activation Is Important for Renal Recovery From AKI,” Journal of the American Society of Nephrology 29, no. 9 (2018): 2372–2385. 30072422 10.1681/ASN.2017121272 PMC6115662

[210] M. M. Rinschen , F. Grahammer , A. K. Hoppe , et al., “YAP‐mediated Mechanotransduction Determines the Podocyte's Response to Damage,” Science Signaling 10, no. 474 (2017): eaaf8165. 28400537 10.1126/scisignal.aaf8165

[211] Y. Liu , Y. Wang , C. Xu , et al., “Activation of the YAP/KLF5 Transcriptional Cascade in Renal Tubular Cells Aggravates Kidney Injury,” Molecular Therapy 32, no. 5 (2024): 1526–1539. 38414248 10.1016/j.ymthe.2024.02.031 PMC11081877

[212] J. Xu , P. X. Li , J. Wu , et al., “Involvement of the Hippo Pathway in Regeneration and Fibrogenesis After Ischaemic Acute Kidney Injury: YAP Is the Key Effector,” Clinical Science (London, England: 1979) 130, no. 5 (2016): 349–363. 26574480 10.1042/CS20150385 PMC4727597

[213] D. J. Hunter and S. Bierma‐Zeinstra , “Osteoarthritis,” Lancet 393, no. 10182 (2019): 1745–1759. 31034380 10.1016/S0140-6736(19)30417-9

[214] J. Martel‐Pelletier , A. J. Barr , F. M. Cicuttini , et al., “Osteoarthritis,” Nature Reviews Disease Primers 2 (2016): 16072. 10.1038/nrdp.2016.72 27734845

[215] A. Bottini , D. J. Wu , R. Ai , et al., “PTPN14 phosphatase and YAP Promote TGFbeta Signalling in Rheumatoid Synoviocytes,” Annals of the Rheumatic Diseases 78, no. 5 (2019): 600–609. 30808624 10.1136/annrheumdis-2018-213799 PMC7039277

[216] A. S. Thorup , D. Strachan , S. Caxaria , et al., “ROR2 blockade as a Therapy for Osteoarthritis,” Science Translational Medicine 12, no. 561 (2020): eaax3063. 32938794 10.1126/scitranslmed.aax3063

[217] Y. Deng , J. Lu , W. Li , et al., “Reciprocal Inhibition of YAP/TAZ and NF‐kappaB Regulates Osteoarthritic Cartilage Degradation,” Nature Communications 9, no. 1 (2018): 4564. 10.1038/s41467-018-07022-2 PMC6212432 30385786

[218] T. van der Poll , M. Shankar‐Hari , and W. J. Wiersinga , “The Immunology of Sepsis,” Immunity 54, no. 11 (2021): 2450–2464. 34758337 10.1016/j.immuni.2021.10.012

[219] M. Cecconi , L. Evans , M. Levy , and A. Rhodes , “Sepsis and Septic Shock,” Lancet 392, no. 10141 (2018): 75–87. 29937192 10.1016/S0140-6736(18)30696-2

[220] R. S. Hotchkiss , L. L. Moldawer , S. M. Opal , K. Reinhart , I. R. Turnbull , and J. L. Vincent , “Sepsis and Septic Shock,” Nature Reviews Disease Primers 2 (2016): 16045. 10.1038/nrdp.2016.45 PMC5538252 28117397

[221] K. Yang , M. Fan , X. Wang , et al., “Lactate Promotes Macrophage HMGB1 Lactylation, Acetylation, and Exosomal Release in Polymicrobial Sepsis,” Cell Death and Differentiation 29, no. 1 (2022): 133–146. 34363018 10.1038/s41418-021-00841-9 PMC8738735

[222] S. Chatterjee , K. Khunti , and M. J. Davies , “Type 2 Diabetes,” Lancet 389, no. 10085 (2017): 2239–2251. 28190580 10.1016/S0140-6736(17)30058-2

[223] F. H. El‐Khatib , C. Balliro , M. A. Hillard , et al., “Home Use of a Bihormonal Bionic Pancreas versus Insulin Pump Therapy in Adults With Type 1 Diabetes: A Multicentre Randomised Crossover Trial,” Lancet 389, no. 10067 (2017): 369–380. 28007348 10.1016/S0140-6736(16)32567-3 PMC5358809

[224] M. L. Chao , S. Luo , C. Zhang , et al., “S‐nitrosylation‐mediated Coupling of G‐protein Alpha‐2 With CXCR5 Induces Hippo/YAP‐dependent Diabetes‐accelerated Atherosclerosis,” Nature Communications 12, no. 1 (2021): 4452. 10.1038/s41467-021-24736-y PMC8298471 34294713

[225] L. Kang , J. Yi , C. W. Lau , et al., “AMPK‐Dependent YAP Inhibition Mediates the Protective Effect of Metformin Against Obesity‐Associated Endothelial Dysfunction and Inflammation,” Antioxidants (Basel) 12, no. 9 (2023): 1681. 37759984 10.3390/antiox12091681 PMC10525300

[226] J. Chen and R. C. Harris , “Interaction of the EGF Receptor and the Hippo Pathway in the Diabetic Kidney,” Journal of the American Society of Nephrology 27, no. 6 (2016): 1689–1700. 26453611 10.1681/ASN.2015040415 PMC4884112

[227] J. Chen , X. Wang , Q. He , H. C. Yang , A. B. Fogo , and R. C. Harris , “Inhibition of Transcriptional Coactivator YAP Impairs the Expression and Function of Transcription Factor WT1 in Diabetic Podocyte Injury,” Kidney International 105, no. 6 (2024): 1200–1211. 38423183 10.1016/j.kint.2024.01.038

[228] S. Choi , S. P. Hong , J. H. Bae , et al., “Hyperactivation of YAP/TAZ Drives Alterations in Mesangial Cells Through Stabilization of N‐Myc in Diabetic Nephropathy,” Journal of the American Society of Nephrology 34, no. 5 (2023): 809–828. 36724799 10.1681/ASN.0000000000000075 PMC10125647

[229] M. J. Oudhoff , F. Antignano , A. L. Chenery , et al., “Intestinal Epithelial Cell‐Intrinsic Deletion of Setd7 Identifies Role for Developmental Pathways in Immunity to Helminth Infection,” PLoS Pathogens 12, no. 9 (2016): e1005876. 27598373 10.1371/journal.ppat.1005876 PMC5012677

[230] S. C. Lin , H. C. Lee , P. C. Hou , J. L. Fu , M. H. Wu , and S. J. Tsai , “Targeting Hypoxia‐mediated YAP1 Nuclear Translocation Ameliorates Pathogenesis of Endometriosis Without Compromising Maternal Fertility,” Journal of Pathology 242, no. 4 (2017): 476–487. 28608501 10.1002/path.4922

[231] L. Wang , S. Wang , Y. Shi , et al., “YAP and TAZ Protect Against White Adipocyte Cell Death During Obesity,” Nature Communications 11, no. 1 (2020): 5455. 10.1038/s41467-020-19229-3 PMC7595161 33116140

[232] W. Pan , L. Yang , J. Li , et al., “Traumatic Occlusion Aggravates Bone Loss During Periodontitis and Activates Hippo‐YAP Pathway,” Journal of Clinical Periodontology 46, no. 4 (2019): 438–447. 30629753 10.1111/jcpe.13065

[233] D. D. Shao , W. Xue , E. B. Krall , et al., “KRAS and YAP1 Converge to Regulate EMT and Tumor Survival,” Cell 158, no. 1 (2014): 171–184. 24954536 10.1016/j.cell.2014.06.004 PMC4110062

[234] J. D. Pearson , K. Huang , M. Pacal , et al., “Binary Pan‐cancer Classes With Distinct Vulnerabilities Defined by Pro‐ or Anti‐cancer YAP/TEAD Activity,” Cancer Cell 39, no. 8 (2021): 1115–1134. e1112. 34270926 10.1016/j.ccell.2021.06.016 PMC8981970

[235] I. Bado and X. H. Zhang , “Senesce to Survive: YAP‐Mediated Dormancy Escapes EGFR/MEK Inhibition,” Cancer Cell 37, no. 1 (2020): 1–2. 31951560 10.1016/j.ccell.2019.12.008

[236] J. A. Ajani , Y. Xu , L. Huo , et al., “YAP1 mediates Gastric Adenocarcinoma Peritoneal Metastases That Are Attenuated by YAP1 Inhibition,” Gut 70, no. 1 (2021): 55–66. 32345613 10.1136/gutjnl-2019-319748 PMC9832914

[237] F. Zanconato , G. Battilana , M. Forcato , et al., “Transcriptional Addiction in Cancer Cells Is Mediated by YAP/TAZ Through BRD4,” Nature Medicine 24, no. 10 (2018): 1599–1610. 10.1038/s41591-018-0158-8 PMC6181206 30224758

[238] F. Zanconato , M. Forcato , G. Battilana , et al., “Genome‐wide Association Between YAP/TAZ/TEAD and AP‐1 at Enhancers Drives Oncogenic Growth,” Nature Cell Biology 17, no. 9 (2015): 1218–1227. 26258633 10.1038/ncb3216 PMC6186417

[239] C. Peng , Y. Zhu , W. Zhang , et al., “Regulation of the Hippo‐YAP Pathway by Glucose Sensor O‐GlcNAcylation,” Molecular Cell 68, no. 3 (2017): 591–604. e595. 29100056 10.1016/j.molcel.2017.10.010

[240] D. E. Pefani , D. Pankova , A. G. Abraham , et al., “TGF‐beta Targets the Hippo Pathway Scaffold RASSF1A to Facilitate YAP/SMAD2 Nuclear Translocation,” Molecular Cell 63, no. 1 (2016): 156–166. 27292796 10.1016/j.molcel.2016.05.012

[241] I. Marigo , R. Trovato , F. Hofer , et al., “Disabled Homolog 2 Controls Prometastatic Activity of Tumor‐Associated Macrophages,” Cancer Discovery 10, no. 11 (2020): 1758–1773. 32651166 10.1158/2159-8290.CD-20-0036

[242] D. Chen , Y. Sun , Y. Wei , et al., “LIFR Is a Breast Cancer Metastasis Suppressor Upstream of the Hippo‐YAP Pathway and a Prognostic Marker,” Nature Medicine 18, no. 10 (2012): 1511–1517. 10.1038/nm.2940 PMC3684419 23001183

[243] X. Ni , J. Tao , J. Barbi , et al., “YAP Is Essential for Treg‐Mediated Suppression of Antitumor Immunity,” Cancer Discovery 8, no. 8 (2018): 1026–1043. 29907586 10.1158/2159-8290.CD-17-1124 PMC6481611

[244] F. Li , Y. Xu , B. Liu , et al., “YAP1‐Mediated CDK6 Activation Confers Radiation Resistance in Esophageal Cancer—Rationale for the Combination of YAP1 and CDK4/6 Inhibitors in Esophageal Cancer,” Clinical Cancer Research 25, no. 7 (2019): 2264–2277. 30563933 10.1158/1078-0432.CCR-18-1029

[245] Z. Li , P. Razavi , Q. Li , et al., “Loss of the FAT1 Tumor Suppressor Promotes Resistance to CDK4/6 Inhibitors via the Hippo Pathway,” Cancer Cell 34, no. 6 (2018): 893–905. e898. 30537512 10.1016/j.ccell.2018.11.006 PMC6294301

[246] K. Du , R. Maeso‐Diaz , S. H. Oh , et al., “Targeting YAP‐mediated HSC Death Susceptibility and Senescence for Treatment of Liver Fibrosis,” Hepatology 77, no. 6 (2023): 1998–2015. 36815382 10.1097/HEP.0000000000000326 PMC10416614

[247] Y. Gu , T. Xu , Y. Fang , et al., “CBX4 counteracts Cellular Senescence to Desensitize Gastric Cancer Cells to Chemotherapy by Inducing YAP1 SUMOylation,” Drug Resistance Updates 77 (2024): 101136. 39154499 10.1016/j.drup.2024.101136

[248] A. Claude‐Taupin , P. Isnard , A. Bagattin , et al., “The AMPK‐Sirtuin 1‐YAP Axis Is Regulated by Fluid Flow Intensity and Controls Autophagy Flux in Kidney Epithelial Cells,” Nature Communications 14, no. 1 (2023): 8056. 10.1038/s41467-023-43775-1 PMC10698145 38052799

[249] S. Ikeda , J. Nah , A. Shirakabe , et al., “YAP Plays a Crucial Role in the Development of Cardiomyopathy in Lysosomal Storage Diseases,” Journal of Clinical Investigation 131, no. 5 (2021): e143173. 33373332 10.1172/JCI143173 PMC7919732

[250] T. P. Neufeld , “Hippo Signaling: Autophagy Waits in the Wings,” Developmental Cell 52, no. 5 (2020): 544–545. 32155435 10.1016/j.devcel.2020.02.014

[251] G. Seo , C. Yu , H. Han , et al., “The Hippo Pathway Noncanonically Drives Autophagy and Cell Survival in Response to Energy Stress,” Molecular Cell 83, no. 17 (2023): 3155–3170. e3158. 37595580 10.1016/j.molcel.2023.07.019 PMC10568779

[252] X. Wang , A. Freire Valls , G. Schermann , et al., “YAP/TAZ Orchestrate VEGF Signaling During Developmental Angiogenesis,” Developmental Cell 42, no. 5 (2017): 462–478. e467. 28867486 10.1016/j.devcel.2017.08.002

[253] A. L. Elaimy and A. M. Mercurio , “Convergence of VEGF and YAP/TAZ Signaling: Implications for Angiogenesis and Cancer Biology,” Science Signaling 11, no. 552 (2018): eaau1165. 30327408 10.1126/scisignal.aau1165 PMC6525620

[254] C. H. Hung , S. Y. Wu , C. D. Yao , et al., “Defective N‐glycosylation of IL6 Induces Metastasis and Tyrosine Kinase Inhibitor Resistance in Lung Cancer,” Nature Communications 15, no. 1 (2024): 7885. 10.1038/s41467-024-51831-7 PMC11385228 39251588

[255] E. Stampouloglou , N. Cheng , A. Federico , et al., “Yap Suppresses T‐cell Function and Infiltration in the Tumor Microenvironment,” PLos Biology 18, no. 1 (2020): e3000591. 31929526 10.1371/journal.pbio.3000591 PMC6980695

[256] A. Lebid , L. Chung , D. M. Pardoll , and F. Pan , “YAP Attenuates CD8 T Cell‐Mediated Anti‐tumor Response,” Frontiers in Immunology 11 (2020): 580. 32322254 10.3389/fimmu.2020.00580 PMC7158852

[257] D. Matthaios , M. Tolia , D. Mauri , K. Kamposioras , and M. Karamouzis , “YAP/Hippo Pathway and Cancer Immunity: It Takes Two to Tango,” Biomedicines 9, no. 12 (2021): 1949. 34944765 10.3390/biomedicines9121949 PMC8698579

[258] J. Bai , M. Yan , Y. Xu , et al., “YAP Enhances Mitochondrial OXPHOS in Tumor‐infiltrating Treg Through Upregulating Lars2 on Stiff Matrix,” Journal for ImmunoTherapy of Cancer 12, no. 11 (2024): e010463. 39551603 10.1136/jitc-2024-010463 PMC11574482

[259] Y. Fan , Y. Gao , J. Rao , K. Wang , F. Zhang , and C. Zhang , “YAP‐1 Promotes Tregs Differentiation in Hepatocellular Carcinoma by Enhancing TGFBR2 Transcription,” Cellular Physiology and Biochemistry 41, no. 3 (2017): 1189–1198. 28472799 10.1159/000464380

[260] J. H. Koo and K. L. Guan , “Interplay Between YAP/TAZ and Metabolism,” Cell Metabolism 28, no. 2 (2018): 196–206. 30089241 10.1016/j.cmet.2018.07.010

[261] K. H. Wrighton , “Metabolism: YAP and TAZ Under Metabolic Control,” Nature Reviews Molecular Cell Biology 15, no. 5 (2014): 296. 10.1038/nrm3796 24739739

[262] S. M. White , M. L. Avantaggiati , I. Nemazanyy , et al., “YAP/TAZ Inhibition Induces Metabolic and Signaling Rewiring Resulting in Targetable Vulnerabilities in NF2‐Deficient Tumor Cells,” Developmental Cell 49, no. 3 (2019): 425–443. e429. 31063758 10.1016/j.devcel.2019.04.014 PMC6524954

[263] M. Lv , Y. Gong , X. Liu , et al., “CDK7‐YAP‐LDHD Axis Promotes D‐lactate Elimination and Ferroptosis Defense to Support Cancer Stem Cell‐Like Properties,” Signal Transduction and Targeted Therapy 8, no. 1 (2023): 302. 37582812 10.1038/s41392-023-01555-9 PMC10427695

[264] X. Zhang , A. Abdelrahman , B. Vollmar , and D. Zechner , “The Ambivalent Function of YAP in Apoptosis and Cancer,” International Journal of Molecular Sciences 19, no. 12 (2018): 3770. 30486435 10.3390/ijms19123770 PMC6321280

[265] Q. Wu , J. Guo , Y. Liu , et al., “YAP Drives Fate Conversion and Chemoresistance of Small Cell Lung Cancer,” Science Advances 7, no. 40 (2021): eabg1850. 34597132 10.1126/sciadv.abg1850 PMC10938532

[266] A. L. Elaimy , J. J. Amante , L. J. Zhu , et al., “The VEGF Receptor Neuropilin 2 Promotes Homologous Recombination by Stimulating YAP/TAZ‐mediated Rad51 Expression,” Proceeding of the National Academy of Sciences in the United States of America 116, no. 28 (2019): 14174–14180. 10.1073/pnas.1821194116 PMC6628806 31235595

[267] F. Zanconato , M. Cordenonsi , and S. Piccolo , “YAP/TAZ at the Roots of Cancer,” Cancer Cell 29, no. 6 (2016): 783–803. 27300434 10.1016/j.ccell.2016.05.005 PMC6186419

[268] Y. Shen , X. Wang , Y. Liu , et al., “STAT3‐YAP/TAZ Signaling in Endothelial Cells Promotes Tumor Angiogenesis,” Science Signaling 14, no. 712 (2021): eabj8393. 34874746 10.1126/scisignal.abj8393

[269] H. Croizer , R. Mhaidly , Y. Kieffer , et al., “Deciphering the Spatial Landscape and Plasticity of Immunosuppressive Fibroblasts in Breast Cancer,” Nature Communications 15, no. 1 (2024): 2806. 10.1038/s41467-024-47068-z PMC10984943 38561380

[270] J. Barbazan , C. Perez‐Gonzalez , M. Gomez‐Gonzalez , et al., “Cancer‐associated Fibroblasts Actively Compress Cancer Cells and Modulate Mechanotransduction,” Nature Communications 14, no. 1 (2023): 6966. 10.1038/s41467-023-42382-4 PMC10618488 37907483

[271] W. Chen , W. Peng , R. Wang , et al., “Exosome‐derived tRNA Fragments tRF‐GluCTC‐0005 Promotes Pancreatic Cancer Liver Metastasis by Activating Hepatic Stellate Cells,” Cell Death & Disease 15, no. 1 (2024): 102. 38291031 10.1038/s41419-024-06482-3 PMC10827722

[272] W. Yu , C. Zhang , Y. Wang , et al., “YAP 5‐methylcytosine Modification Increases Its mRNA Stability and Promotes the Transcription of Exosome Secretion‐related Genes in Lung Adenocarcinoma,” Cancer Gene Therapy 30, no. 1 (2023): 149–162. 36123390 10.1038/s41417-022-00533-7 PMC9842506

[273] D. E. Johnson , B. Burtness , C. R. Leemans , V. W. Y. Lui , J. E. Bauman , and J. R. Grandis , “Head and Neck Squamous Cell Carcinoma,” Nature Reviews Disease Primers 6, no. 1 (2020): 92. 10.1038/s41572-020-00224-3 PMC7944998 33243986

[274] M. D. Mody , J. W. Rocco , S. S. Yom , R. I. Haddad , and N. F. Saba , “Head and Neck Cancer,” Lancet 398, no. 10318 (2021): 2289–2299. 34562395 10.1016/S0140-6736(21)01550-6

[275] L. Boucai , M. Zafereo , and M. E. Cabanillas , “Thyroid Cancer: A Review,” Jama 331, no. 5 (2024): 425–435. 38319329 10.1001/jama.2023.26348

[276] Y. P. Chen , A. T. C. Chan , Q. T. Le , P. Blanchard , Y. Sun , and J. Ma , “Nasopharyngeal Carcinoma,” Lancet 394, no. 10192 (2019): 64–80. 31178151 10.1016/S0140-6736(19)30956-0

[277] C. E. Steuer , M. El‐Deiry , J. R. Parks , K. A. Higgins , and N. F. Saba , “An Update on Larynx Cancer,” CA: A Cancer Journal for Clinicians 67, no. 1 (2017): 31–50. 27898173 10.3322/caac.21386

[278] A. C. Chi , T. A. Day , and B. W. Neville , “Oral Cavity and Oropharyngeal Squamous Cell Carcinoma–an Update,” CA: A Cancer Journal for Clinicians 65, no. 5 (2015): 401–421. 26215712 10.3322/caac.21293

[279] B. W. Neville and T. A. Day , “Oral Cancer and Precancerous Lesions,” CA: A Cancer Journal for Clinicians 52, no. 4 (2002): 195–215. 12139232 10.3322/canjclin.52.4.195

[280] J. L. Llorente , F. Lopez , C. Suarez , and M. A. Hermsen , “Sinonasal Carcinoma: Clinical, Pathological, Genetic and Therapeutic Advances,” Nature Reviews Clinical Oncology 11, no. 8 (2014): 460–472. 10.1038/nrclinonc.2014.97 24935016

[281] H. Omori , M. Nishio , M. Masuda , et al., “YAP1 is a Potent Driver of the Onset and Progression of Oral Squamous Cell Carcinoma,” Science Advances 6, no. 12 (2020): eaay3324. 32206709 10.1126/sciadv.aay3324 PMC7080500

[282] S. V. Saladi , K. Ross , M. Karaayvaz , et al., “ACTL6A Is Co‐Amplified With p63 in Squamous Cell Carcinoma to Drive YAP Activation, Regenerative Proliferation, and Poor Prognosis,” Cancer Cell 31, no. 1 (2017): 35–49. 28041841 10.1016/j.ccell.2016.12.001 PMC5225026

[283] K. C. W. Wong , E. P. Hui , K. W. Lo , et al., “Nasopharyngeal Carcinoma: An Evolving Paradigm,” Nature Reviews Clinical Oncology 18, no. 11 (2021): 679–695. 10.1038/s41571-021-00524-x 34194007

[284] A. W. Lee , B. B. Ma , W. T. Ng , and A. T. Chan , “Management of Nasopharyngeal Carcinoma: Current Practice and Future Perspective,” Journal of Clinical Oncology 33, no. 29 (2015): 3356–3364. 26351355 10.1200/JCO.2015.60.9347

[285] K. W. Lo , K. F. To , and D. P. Huang , “Focus on Nasopharyngeal Carcinoma,” Cancer Cell 5, no. 5 (2004): 423–428. 15144950 10.1016/s1535-6108(04)00119-9

[286] C. Y. Lee , T. H. Wang , and Y. S. Kao , “Chemotherapy, Radiation Therapy, and Nasopharyngeal Carcinoma,” JAMA Oncology 10, no. 9 (2024): 1292–1293. 10.1001/jamaoncol.2024.2522 38990530

[287] Y. P. Liu , Y. H. Wen , J. Tang , et al., “Endoscopic Surgery Compared With Intensity‐modulated Radiotherapy in Resectable Locally Recurrent Nasopharyngeal Carcinoma: A Multicentre, Open‐label, Randomised, Controlled, Phase 3 Trial,” The Lancet Oncology 22, no. 3 (2021): 381–390. 33600761 10.1016/S1470-2045(20)30673-2

[288] P. Wu , X. Hou , M. Peng , et al., “Circular RNA circRILPL1 Promotes Nasopharyngeal Carcinoma Malignant Progression by Activating the Hippo‐YAP Signaling Pathway,” Cell Death and Differentiation 30, no. 7 (2023): 1679–1694. 37173390 10.1038/s41418-023-01171-8 PMC10307875

[289] S. C. Liu , T. Hsu , Y. S. Chang , et al., “Cytoplasmic LIF Reprograms Invasive Mode to Enhance NPC Dissemination Through Modulating YAP1‐FAK/PXN Signaling,” Nature Communications 9, no. 1 (2018): 5105. 10.1038/s41467-018-07660-6 PMC6269507 30504771

[290] S. Beheshtirouy and A. Shayanfar , “A Review of Thyroid Cancer,” Jama 331, no. 21 (2024): 1862–1863. 38709522 10.1001/jama.2024.5998

[291] E. K. Alexander and E. S. Cibas , “Diagnosis of Thyroid Nodules,” The Lancet Diabetes & Endocrinology 10, no. 7 (2022): 533–539. 35752200 10.1016/S2213-8587(22)00101-2

[292] C. M. Kitahara and J. A. Sosa , “The Changing Incidence of Thyroid Cancer,” Nature Reviews Endocrinology 12, no. 11 (2016): 646–653. 10.1038/nrendo.2016.110 PMC10311569 27418023

[293] Y. E. Nikiforov and M. N. Nikiforova , “Molecular Genetics and Diagnosis of Thyroid Cancer,” Nature Reviews Endocrinology 7, no. 10 (2011): 569–580. 10.1038/nrendo.2011.142 21878896

[294] J. Tang , Q. Yang , C. Mao , et al., “The Deubiquitinating Enzyme UCHL3 Promotes Anaplastic Thyroid Cancer Progression and Metastasis Through Hippo Signaling Pathway,” Cell Death and Differentiation 30, no. 5 (2023): 1247–1259. 36813921 10.1038/s41418-023-01134-z PMC10154385

[295] M. E. Garcia‐Rendueles , J. C. Ricarte‐Filho , B. R. Untch , et al., “NF2 Loss Promotes Oncogenic RAS‐Induced Thyroid Cancers via YAP‐Dependent Transactivation of RAS Proteins and Sensitizes Them to MEK Inhibition,” Cancer Discovery 5, no. 11 (2015): 1178–1193. 26359368 10.1158/2159-8290.CD-15-0330 PMC4642441

[296] K. J. Wang , C. Wang , L. H. Dai , et al., “Targeting an Autocrine Regulatory Loop in Cancer Stem‐Like Cells Impairs the Progression and Chemotherapy Resistance of Bladder Cancer,” Clinical Cancer Research 25, no. 3 (2019): 1070–1086. 30397177 10.1158/1078-0432.CCR-18-0586

[297] B. C. Widemann and A. Italiano , “Biology and Management of Undifferentiated Pleomorphic Sarcoma, Myxofibrosarcoma, and Malignant Peripheral Nerve Sheath Tumors: State of the Art and Perspectives,” Journal of Clinical Oncology 36, no. 2 (2018): 160–167. 29220302 10.1200/JCO.2017.75.3467 PMC5759316

[298] W. Mo , J. Chen , A. Patel , et al., “CXCR4/CXCL12 mediate Autocrine Cell‐ cycle Progression in NF1‐associated Malignant Peripheral Nerve Sheath Tumors,” Cell 152, no. 5 (2013): 1077–1090. 23434321 10.1016/j.cell.2013.01.053 PMC3594500

[299] F. J. Rodriguez , A. L. Folpe , C. Giannini , and A. Perry , “Pathology of Peripheral Nerve Sheath Tumors: Diagnostic Overview and Update on Selected Diagnostic Problems,” Acta Neuropathologica 123, no. 3 (2012): 295–319. 22327363 10.1007/s00401-012-0954-z PMC3629555

[300] L. M. N. Wu , Y. Deng , J. Wang , et al., “Programming of Schwann Cells by Lats1/2‐TAZ/YAP Signaling Drives Malignant Peripheral Nerve Sheath Tumorigenesis,” Cancer Cell 33, no. 2 (2018): 292–308. e297. 29438698 10.1016/j.ccell.2018.01.005 PMC5813693

[301] M. L. Feltri and Y. Poitelon , “HIPPO Stampede in Nerve Sheath Tumors,” Cancer Cell 33, no. 2 (2018): 160–161. 29438691 10.1016/j.ccell.2018.01.016

[302] L. Laraba , L. Hillson , J. G. de Guibert , et al., “Inhibition of YAP/TAZ‐driven TEAD Activity Prevents Growth of NF2‐null Schwannoma and Meningioma,” Brain 146, no. 4 (2023): 1697–1713. 36148553 10.1093/brain/awac342 PMC10115179

[303] G. V. Long , S. M. Swetter , A. M. Menzies , J. E. Gershenwald , and R. A. Scolyer , “Cutaneous Melanoma,” Lancet 402, no. 10400 (2023): 485–502. 37499671 10.1016/S0140-6736(23)00821-8

[304] M. S. Carlino , J. Larkin , and G. V. Long , “Immune Checkpoint Inhibitors in Melanoma,” Lancet 398, no. 10304 (2021): 1002–1014. 34509219 10.1016/S0140-6736(21)01206-X

[305] R. D. Carvajal , J. J. Sacco , M. J. Jager , et al., “Advances in the Clinical Management of Uveal Melanoma,” Nature Reviews Clinical Oncology 20, no. 2 (2023): 99–115. 10.1038/s41571-022-00714-1 36600005

[306] B. D. Curti and M. B. Faries , “Recent Advances in the Treatment of Melanoma,” New England Journal of Medicine 384, no. 23 (2021): 2229–2240. 34107182 10.1056/NEJMra2034861

[307] I. A. M. Barbosa , R. Gopalakrishnan , S. Mercan , et al., “Cancer Lineage‐specific Regulation of YAP Responsive Elements Revealed Through Large‐scale Functional Epigenomic Screens,” Nature Communications 14, no. 1 (2023): 3907. 10.1038/s41467-023-39527-w PMC10317959 37400441

[308] X. Feng , M. S. Degese , R. Iglesias‐Bartolome , et al., “Hippo‐independent Activation of YAP by the GNAQ Uveal Melanoma Oncogene Through a Trio‐regulated Rho GTPase Signaling Circuitry,” Cancer Cell 25, no. 6 (2014): 831–845. 24882515 10.1016/j.ccr.2014.04.016 PMC4074519

[309] X. Feng , N. Arang , D. C. Rigiracciolo , et al., “A Platform of Synthetic Lethal Gene Interaction Networks Reveals That the GNAQ Uveal Melanoma Oncogene Controls the Hippo Pathway Through FAK,” Cancer Cell 35, no. 3 (2019): 457–472. e455. 30773340 10.1016/j.ccell.2019.01.009 PMC6737937

[310] F. X. Yu , J. Luo , J. S. Mo , et al., “Mutant Gq/11 Promote Uveal Melanoma Tumorigenesis by Activating YAP,” Cancer Cell 25, no. 6 (2014): 822–830. 24882516 10.1016/j.ccr.2014.04.017 PMC4075337

[311] A. Truong , J. H. Yoo , M. T. Scherzer , et al., “Chloroquine Sensitizes GNAQ/11‐mutated Melanoma to MEK1/2 Inhibition,” Clinical Cancer Research 26, no. 23 (2020): 6374–6386. 32933997 10.1158/1078-0432.CCR-20-1675 PMC7710560

[312] Y. Xiao , L. Zhou , T. Andl , and Y. Zhang , “YAP1 controls the N‐cadherin‐mediated Tumor‐stroma Interaction in Melanoma Progression,” Oncogene 43, no. 12 (2024): 884–898. 38308096 10.1038/s41388-024-02953-1 PMC10942861

[313] S. M. Swain , M. Shastry , and E. Hamilton , “Targeting HER2‐positive Breast Cancer: Advances and Future Directions,” Nature Reviews Drug Discovery 22, no. 2 (2023): 101–126. 36344672 10.1038/s41573-022-00579-0 PMC9640784

[314] E. Nolan , G. J. Lindeman , and J. E. Visvader , “Deciphering Breast Cancer: From Biology to the Clinic,” Cell 186, no. 8 (2023): 1708–1728. 36931265 10.1016/j.cell.2023.01.040

[315] N. Harbeck , “Breast Cancer Is a Systemic Disease Optimally Treated by a Multidisciplinary Team,” Nature Reviews Disease Primers 6, no. 1 (2020): 30. 10.1038/s41572-020-0167-z 32327646

[316] N. Harbeck , F. Penault‐Llorca , J. Cortes , et al., “Breast Cancer,” Nature Reviews Disease Primers 5, no. 1 (2019): 66. 10.1038/s41572-019-0111-2 31548545

[317] K. L. Britt , J. Cuzick , and K. A. Phillips , “Key Steps for Effective Breast Cancer Prevention,” Nature Reviews Cancer 20, no. 8 (2020): 417–436. 32528185 10.1038/s41568-020-0266-x

[318] M. Will , J. Liang , C. Metcalfe , and S. Chandarlapaty , “Therapeutic Resistance to Anti‐oestrogen Therapy in Breast Cancer,” Nature Reviews Cancer 23, no. 10 (2023): 673–685. 37500767 10.1038/s41568-023-00604-3 PMC10529099

[319] M. A. Harris , P. Savas , B. Virassamy , et al., “Towards Targeting the Breast Cancer Immune Microenvironment,” Nature Reviews Cancer 24, no. 8 (2024): 554–577. 38969810 10.1038/s41568-024-00714-6

[320] R. H. Li , T. Tian , Q. W. Ge , et al., “A Phosphatidic Acid‐binding lncRNA SNHG9 Facilitates LATS1 Liquid‐liquid Phase Separation to Promote Oncogenic YAP Signaling,” Cell Research 31, no. 10 (2021): 1088–1105. 34267352 10.1038/s41422-021-00530-9 PMC8486796

[321] C. Li , S. Wang , Z. Xing , et al., “A ROR1‐HER3‐lncRNA Signalling Axis Modulates the Hippo‐YAP Pathway to Regulate Bone Metastasis,” Nature Cell Biology 19, no. 2 (2017): 106–119. 28114269 10.1038/ncb3464 PMC5336186

[322] B. Ma , Y. Chen , L. Chen , et al., “Hypoxia Regulates Hippo Signalling Through the SIAH2 Ubiquitin E3 Ligase,” Nature Cell Biology 17, no. 1 (2015): 95–103. 25438054 10.1038/ncb3073

[323] S. Ma , Z. Wu , F. Yang , et al., “Hippo Signalling Maintains ER Expression and ER(+) Breast Cancer Growth,” Nature 591, no. 7848 (2021): E1–E10. 33658690 10.1038/s41586-020-03131-5 PMC8725601

[324] C. Zhu , L. Li , Z. Zhang , et al., “A Non‐canonical Role of YAP/TEAD Is Required for Activation of Estrogen‐Regulated Enhancers in Breast Cancer,” Molecular Cell 75, no. 4 (2019): 791–806. e798. 31303470 10.1016/j.molcel.2019.06.010 PMC6707877

[325] H. Han , R. Qi , J. J. Zhou , et al., “Regulation of the Hippo Pathway by Phosphatidic Acid‐Mediated Lipid‐Protein Interaction,” Molecular Cell 72, no. 2 (2018): 328–340. e328. 30293781 10.1016/j.molcel.2018.08.038 PMC6195446

[326] Y. Gao , Y. Yang , F. Yuan , et al., “TNFalpha‐YAP/p65‐HK2 Axis Mediates Breast Cancer Cell Migration,” Oncogenesis 6, no. 9 (2017): e383. 28945218 10.1038/oncsis.2017.83 PMC5623908

[327] S. Wang , E. Englund , P. Kjellman , et al., “CCM3 is a Gatekeeper in Focal Adhesions Regulating Mechanotransduction and YAP/TAZ Signalling,” Nature Cell Biology 23, no. 7 (2021): 758–770. 34226698 10.1038/s41556-021-00702-0

[328] G. Sorrentino , N. Ruggeri , V. Specchia , et al., “Metabolic Control of YAP and TAZ by the Mevalonate Pathway,” Nature Cell Biology 16, no. 4 (2014): 357–366. 24658687 10.1038/ncb2936

[329] J. Zhang , J. Y. Ji , M. Yu , et al., “YAP‐dependent Induction of Amphiregulin Identifies a Non‐Cell‐Autonomous Component of the Hippo Pathway,” Nature Cell Biology 11, no. 12 (2009): 1444–1450. 19935651 10.1038/ncb1993 PMC2819909

[330] J. Kim , H. L. Piao , B. J. Kim , et al., “Long Noncoding RNA MALAT1 Suppresses Breast Cancer Metastasis,” Nature Genetics 50, no. 12 (2018): 1705–1715. 30349115 10.1038/s41588-018-0252-3 PMC6265076

[331] B. von Eyss , L. A. Jaenicke , R. M. Kortlever , et al., “A MYC‐Driven Change in Mitochondrial Dynamics Limits YAP/TAZ Function in Mammary Epithelial Cells and Breast Cancer,” Cancer Cell 28, no. 6 (2015): 743–757. 26678338 10.1016/j.ccell.2015.10.013

[332] E. C. Smyth , M. Nilsson , H. I. Grabsch , N. C. van Grieken , and F. Lordick , “Gastric Cancer,” Lancet 396, no. 10251 (2020): 635–648. 32861308 10.1016/S0140-6736(20)31288-5

[333] E. Van Cutsem , X. Sagaert , B. Topal , K. Haustermans , and H. Prenen , “Gastric Cancer,” Lancet 388, no. 10060 (2016): 2654–2664. 27156933 10.1016/S0140-6736(16)30354-3

[334] S. S. Joshi and B. D. Badgwell , “Current Treatment and Recent Progress in Gastric Cancer,” CA: A Cancer Journal for Clinicians 71, no. 3 (2021): 264–279. 33592120 10.3322/caac.21657 PMC9927927

[335] M. Alsina , V. Arrazubi , M. Diez , and J. Tabernero , “Current Developments in Gastric Cancer: From Molecular Profiling to Treatment Strategy,” Nature Reviews Gastroenterology & Hepatology 20, no. 3 (2023): 155–170. 36344677 10.1038/s41575-022-00703-w

[336] M. Amieva , “Pathobiology of Helicobacter pylori‐Induced Gastric Cancer,” Gastroenterology 150, no. 1 (2016): 64–78. 26385073 10.1053/j.gastro.2015.09.004 PMC4691563

[337] M. H. McLean and E. M. El‐Omar , “Genetics of Gastric Cancer,” Nature Reviews Gastroenterology & Hepatology 11, no. 11 (2014): 664–674. 25134511 10.1038/nrgastro.2014.143

[338] T. Ushijima and M. Sasako , “Focus on Gastric Cancer,” Cancer Cell 5, no. 2 (2004): 121–125. 14998488 10.1016/s1535-6108(04)00033-9

[339] W. Kang , J. H. Tong , A. W. Chan , et al., “Yes‐associated Protein 1 Exhibits Oncogenic Property in Gastric Cancer and Its Nuclear Accumulation Associates With Poor Prognosis,” Clinical Cancer Research 17, no. 8 (2011): 2130–2139. 21346147 10.1158/1078-0432.CCR-10-2467

[340] Y. Tang , G. Fang , F. Guo , et al., “Selective Inhibition of STRN3‐Containing PP2A Phosphatase Restores Hippo Tumor‐Suppressor Activity in Gastric Cancer,” Cancer Cell 38, no. 1 (2020): 115–128. e119. 32589942 10.1016/j.ccell.2020.05.019

[341] L. An , P. Nie , M. Chen , et al., “MST4 kinase Suppresses Gastric Tumorigenesis by Limiting YAP Activation via a Non‐canonical Pathway,” Journal of Experimental Medicine 217, no. 6 (2020): e20191817. 32271880 10.1084/jem.20191817 PMC7971137

[342] N. Li , X. Xu , Y. Zhan , et al., “YAP and Beta‐catenin Cooperate to Drive H. pylori‐induced Gastric Tumorigenesis,” Gut Microbes 15, no. 1 (2023): 2192501. 36959122 10.1080/19490976.2023.2192501 PMC10044160

[343] F. Zhang , V. Sahu , K. Peng , et al., “Recurrent RhoGAP Gene Fusion CLDN18‐ARHGAP26 Promotes RHOA Activation and Focal Adhesion Kinase and YAP‐TEAD Signalling in Diffuse Gastric Cancer,” Gut 73, no. 8 (2024): 1280–1291. 38621923 10.1136/gutjnl-2023-329686 PMC11287566

[344] S. Jiao , J. Guan , M. Chen , et al., “Targeting IRF3 as a YAP Agonist Therapy Against Gastric Cancer,” Journal of Experimental Medicine 215, no. 2 (2018): 699–718. 29339449 10.1084/jem.20171116 PMC5789414

[345] M. Jang , J. An , S. W. Oh , et al., “Matrix Stiffness Epigenetically Regulates the Oncogenic Activation of the Yes‐associated Protein in Gastric Cancer,” Nature Biomedical Engineering 5, no. 1 (2021): 114–123. 10.1038/s41551-020-00657-x 33288878

[346] J. Ju , H. Zhang , M. Lin , et al., “The Alanyl‐tRNA Synthetase AARS1 Moonlights as a Lactyltransferase to Promote YAP Signaling in Gastric Cancer,” Journal of Clinical Investigation 134, no. 10 (2024): e174587. 38512451 10.1172/JCI174587 PMC11093599

[347] P. Nie , W. Zhang , Y. Meng , et al., “A YAP/TAZ‐CD54 Axis Is Required for CXCR2‐CD44‐ tumor‐specific Neutrophils to Suppress Gastric Cancer,” Protein Cell 14, no. 7 (2023): 513–531. 36921037 10.1093/procel/pwac045 PMC10305741

[348] W. Kang , J. H. Tong , R. W. Lung , et al., “Targeting of YAP1 by microRNA‐15a and microRNA‐16‐1 Exerts Tumor Suppressor Function in Gastric Adenocarcinoma,” Molecular Cancer 14 (2015): 52. 25743273 10.1186/s12943-015-0323-3 PMC4342823

[349] S. Jiao , H. Wang , Z. Shi , et al., “A Peptide Mimicking VGLL4 Function Acts as a YAP Antagonist Therapy Against Gastric Cancer,” Cancer Cell 25, no. 2 (2014): 166–180. 24525233 10.1016/j.ccr.2014.01.010

[350] M. A. van den Bosch and L. Defreyne , “Hepatocellular Carcinoma,” Lancet 380, no. 9840 (2012): 469–470. author reply 470‐461. 10.1016/S0140-6736(12)61284-7 22863044

[351] X. Li , P. Ramadori , D. Pfister , M. Seehawer , L. Zender , and M. Heikenwalder , “The Immunological and Metabolic Landscape in Primary and Metastatic Liver Cancer,” Nature Reviews Cancer 21, no. 9 (2021): 541–557. 34326518 10.1038/s41568-021-00383-9

[352] M. Ringelhan , D. Pfister , T. O'Connor , E. Pikarsky , and M. Heikenwalder , “The Immunology of Hepatocellular Carcinoma,” Nature Immunology 19, no. 3 (2018): 222–232. 29379119 10.1038/s41590-018-0044-z

[353] A. G. Singal , F. Kanwal , and J. M. Llovet , “Global Trends in Hepatocellular Carcinoma Epidemiology: Implications for Screening, Prevention and Therapy,” Nature Reviews Clinical Oncology 20, no. 12 (2023): 864–884. 10.1038/s41571-023-00825-3 37884736

[354] J. M. Llovet , R. Montal , D. Sia , and R. S. Finn , “Molecular Therapies and Precision Medicine for Hepatocellular Carcinoma,” Nature Reviews Clinical Oncology 15, no. 10 (2018): 599–616. 10.1038/s41571-018-0073-4 PMC12452113 30061739

[355] S. Abitbol , R. Dahmani , C. Coulouarn , et al., “AXIN Deficiency in human and Mouse Hepatocytes Induces Hepatocellular Carcinoma in the Absence of Beta‐catenin Activation,” Journal of Hepatology 68, no. 6 (2018): 1203–1213. 29525529 10.1016/j.jhep.2017.12.018

[356] L. Zender , M. S. Spector , W. Xue , et al., “Identification and Validation of Oncogenes in Liver Cancer Using an Integrative Oncogenomic Approach,” Cell 125, no. 7 (2006): 1253–1267. 16814713 10.1016/j.cell.2006.05.030 PMC3026384

[357] A. Perra , M. A. Kowalik , E. Ghiso , et al., “YAP Activation Is an Early Event and a Potential Therapeutic Target in Liver Cancer Development,” Journal of Hepatology 61, no. 5 (2014): 1088–1096. 25010260 10.1016/j.jhep.2014.06.033

[358] S. M. E. Weiler , F. Pinna , T. Wolf , et al., “Induction of Chromosome Instability by Activation of Yes‐Associated Protein and Forkhead Box M1 in Liver Cancer,” Gastroenterology 152, no. 8 (2017): 2037–2051. e2022. 28249813 10.1053/j.gastro.2017.02.018

[359] S. H. Patel , F. D. Camargo , and D. Yimlamai , “Hippo Signaling in the Liver Regulates Organ Size, Cell Fate, and Carcinogenesis,” Gastroenterology 152, no. 3 (2017): 533–545. 28003097 10.1053/j.gastro.2016.10.047 PMC5285449

[360] K. Cho , S. W. Ro , H. W. Lee , et al., “YAP/TAZ Suppress Drug Penetration into Hepatocellular Carcinoma through Stromal Activation,” Hepatology 74, no. 5 (2021): 2605–2621. 34101869 10.1002/hep.32000

[361] Y. Tian , B. Yang , W. Qiu , et al., “ER‐residential Nogo‐B Accelerates NAFLD‐associated HCC Mediated by Metabolic Reprogramming of oxLDL Lipophagy,” Nature Communications 10, no. 1 (2019): 3391. 10.1038/s41467-019-11274-x PMC6662851 31358770

[362] X. Sun , Y. Ding , M. Zhan , et al., “Usp7 regulates Hippo Pathway Through Deubiquitinating the Transcriptional Coactivator Yorkie,” Nature Communications 10, no. 1 (2019): 411. 10.1038/s41467-019-08334-7 PMC6345853 30679505

[363] M. A. Kowalik , C. Saliba , M. Pibiri , et al., “Yes‐associated Protein Regulation of Adaptive Liver Enlargement and Hepatocellular Carcinoma Development in Mice,” Hepatology 53, no. 6 (2011): 2086–2096. 21391223 10.1002/hep.24289

[364] H. Wang , S. Zhang , Y. Zhang , et al., “TAZ Is Indispensable for c‐MYC‐induced Hepatocarcinogenesis,” Journal of Hepatology 76, no. 1 (2022): 123–134. 34464659 10.1016/j.jhep.2021.08.021 PMC9569156

[365] R. Urtasun , M. U. Latasa , M. I. Demartis , et al., “Connective Tissue Growth Factor Autocriny in human Hepatocellular Carcinoma: Oncogenic Role and Regulation by Epidermal Growth Factor Receptor/Yes‐associated Protein‐mediated Activation,” Hepatology 54, no. 6 (2011): 2149–2158. 21800344 10.1002/hep.24587

[366] W. Ni , Y. Zhang , Z. Zhan , et al., “A Novel lncRNA Uc.134 Represses Hepatocellular Carcinoma Progression by Inhibiting CUL4A‐mediated Ubiquitination of LATS1,” Journal of Hematology & Oncology 10, no. 1 (2017): 91. 28420424 10.1186/s13045-017-0449-4 PMC5395742

[367] D. Su , Y. Li , W. Zhang , et al., “SPTAN1/NUMB Axis Senses Cell Density to Restrain Cell Growth and Oncogenesis Through Hippo Signaling,” Journal of Clinical Investigation 133, no. 20 (2023): e168888. 37843276 10.1172/JCI168888 PMC10575737

[368] A. Hermann , D. O. Wennmann , S. Gromnitza , et al., “WW and C2 Domain‐containing Proteins Regulate Hepatic Cell Differentiation and Tumorigenesis Through the Hippo Signaling Pathway,” Hepatology 67, no. 4 (2018): 1546–1559. 29116649 10.1002/hep.29647

[369] J. Hyun , M. Al Abo , R. K. Dutta , et al., “Dysregulation of the ESRP2‐NF2‐YAP/TAZ Axis Promotes Hepatobiliary Carcinogenesis in Non‐alcoholic Fatty Liver Disease,” Journal of Hepatology 75, no. 3 (2021): 623–633. 33964370 10.1016/j.jhep.2021.04.033 PMC8380690

[370] X. M. Yang , X. Y. Cao , P. He , et al., “Overexpression of Rac GTPase Activating Protein 1 Contributes to Proliferation of Cancer Cells by Reducing Hippo Signaling to Promote Cytokinesis,” Gastroenterology 155, no. 4 (2018): 1233–1249. e1222. 30009820 10.1053/j.gastro.2018.07.010

[371] Y. X. Xiong , X. C. Zhang , J. H. Zhu , et al., “Collagen I‐DDR1 Signaling Promotes Hepatocellular Carcinoma Cell Stemness via Hippo Signaling Repression,” Cell Death and Differentiation 30, no. 7 (2023): 1648–1665. 37117273 10.1038/s41418-023-01166-5 PMC10307904

[372] W. Kim , S. K. Khan , Y. Liu , et al., “Hepatic Hippo Signaling Inhibits Protumoural Microenvironment to Suppress Hepatocellular Carcinoma,” Gut 67, no. 9 (2018): 1692–1703. 28866620 10.1136/gutjnl-2017-314061 PMC6592016

[373] S. Zhang , Q. Chen , Q. Liu , et al., “Hippo Signaling Suppresses Cell Ploidy and Tumorigenesis Through Skp2,” Cancer Cell 31, no. 5 (2017): 669–684. e667. 28486106 10.1016/j.ccell.2017.04.004 PMC5863541

[374] D. Zhou , C. Conrad , F. Xia , et al., “Mst1 and Mst2 Maintain Hepatocyte Quiescence and Suppress Hepatocellular Carcinoma Development Through Inactivation of the Yap1 Oncogene,” Cancer Cell 16, no. 5 (2009): 425–438. 19878874 10.1016/j.ccr.2009.09.026 PMC3023165

[375] T. Zhang , J. Zhang , X. You , et al., “Hepatitis B Virus X Protein Modulates Oncogene Yes‐associated Protein by CREB to Promote Growth of Hepatoma Cells,” Hepatology 56, no. 6 (2012): 2051–2059. 22707013 10.1002/hep.25899

[376] T. J. Hagenbeek , J. D. Webster , N. M. Kljavin , et al., “The Hippo Pathway Effector TAZ Induces TEAD‐dependent Liver Inflammation and Tumors,” Science Signaling 11, no. 547 (2018): eaaj1757. 30206136 10.1126/scisignal.aaj1757

[377] B. Liang , Y. Zhou , M. Qian , et al., “TBX3 functions as a Tumor Suppressor Downstream of Activated CTNNB1 Mutants During Hepatocarcinogenesis,” Journal of Hepatology 75, no. 1 (2021): 120–131. 33577921 10.1016/j.jhep.2021.01.044 PMC8217095

[378] K. Tu , W. Yang , C. Li , et al., “Fbxw7 is an Independent Prognostic Marker and Induces Apoptosis and Growth Arrest by Regulating YAP Abundance in Hepatocellular Carcinoma,” Molecular Cancer 13 (2014): 110. 24884509 10.1186/1476-4598-13-110 PMC4035898

[379] H. Wang , X. Song , H. Liao , et al., “Overexpression of Mothers against Decapentaplegic Homolog 7 Activates the Yes‐Associated Protein/NOTCH Cascade and Promotes Liver Carcinogenesis in Mice and Humans,” Hepatology 74, no. 1 (2021): 248–263. 33368437 10.1002/hep.31692 PMC8222417

[380] R. Chen , S. Zhu , X. G. Fan , et al., “High Mobility Group Protein B1 Controls Liver Cancer Initiation Through Yes‐associated Protein ‐dependent Aerobic Glycolysis,” Hepatology 67, no. 5 (2018): 1823–1841. 29149457 10.1002/hep.29663 PMC5906197

[381] W. X. Ding and P. Sancho‐Bru , “SOX9 acts Downstream of YAP to Decide Liver Cell Fate and Tumor Types,” Journal of Hepatology 76, no. 3 (2022): 503–505. 34929213 10.1016/j.jhep.2021.12.008

[382] K. Li , J. Zhang , H. Lyu , et al., “CSN6‐SPOP‐HMGCS1 Axis Promotes Hepatocellular Carcinoma Progression via YAP1 Activation,” Advanced Science (Weinh) 11, no. 14 (2024): e2306827. 10.1002/advs.202306827 PMC11005689 38308184

[383] Y. Saito , D. Yin , N. Kubota , et al., “A Therapeutically Targetable TAZ‐TEAD2 Pathway Drives the Growth of Hepatocellular Carcinoma via ANLN and KIF23,” Gastroenterology 164, no. 7 (2023): 1279–1292. 36894036 10.1053/j.gastro.2023.02.043 PMC10335360

[384] J. Tao , D. F. Calvisi , S. Ranganathan , et al., “Activation of Beta‐catenin and Yap1 in human Hepatoblastoma and Induction of Hepatocarcinogenesis in Mice,” Gastroenterology 147, no. 3 (2014): 690–701. 24837480 10.1053/j.gastro.2014.05.004 PMC4143445

[385] M. Lin , X. Zheng , J. Yan , et al., “The RNF214‐TEAD‐YAP Signaling Axis Promotes Hepatocellular Carcinoma Progression via TEAD Ubiquitylation,” Nature Communications 15, no. 1 (2024): 4995. 10.1038/s41467-024-49045-y PMC11167002 38862474

[386] B. Liang , H. Wang , Y. Qiao , et al., “Differential Requirement of Hippo Cascade During CTNNB1 or AXIN1 Mutation‐driven Hepatocarcinogenesis,” Hepatology 77, no. 6 (2023): 1929–1942. 35921500 10.1002/hep.32693 PMC10572102

[387] T. Yuan , T. Zhou , M. Qian , et al., “SDHA/B Reduction Promotes Hepatocellular Carcinoma by Facilitating the deNEDDylation of cullin1 and Stabilizing YAP/TAZ,” Hepatology 78, no. 1 (2023): 103–119. 35713976 10.1002/hep.32621

[388] W. C. Yuan , B. Pepe‐Mooney , G. G. Galli , et al., “NUAK2 is a Critical YAP Target in Liver Cancer,” Nature Communications 9, no. 1 (2018): 4834. 10.1038/s41467-018-07394-5 PMC6240092 30446657

[389] W. Kim , S. K. Khan , J. Gvozdenovic‐Jeremic , et al., “Hippo Signaling Interactions With Wnt/Beta‐catenin and Notch Signaling Repress Liver Tumorigenesis,” Journal of Clinical Investigation 127, no. 1 (2017): 137–152. 27869648 10.1172/JCI88486 PMC5199712

[390] W. Y. Cai , L. Y. Lin , H. Hao , et al., “Yes‐associated Protein/TEA Domain family Member and Hepatocyte Nuclear Factor 4‐alpha (HNF4alpha) Repress Reciprocally to Regulate Hepatocarcinogenesis in Rats and Mice,” Hepatology 65, no. 4 (2017): 1206–1221. 27809333 10.1002/hep.28911

[391] J. Wang , L. Ma , W. Weng , et al., “Mutual Interaction Between YAP and CREB Promotes Tumorigenesis in Liver Cancer,” Hepatology 58, no. 3 (2013): 1011–1020. 23532963 10.1002/hep.26420

[392] J. Wang , J. S. Park , Y. Wei , et al., “TRIB2 acts Downstream of Wnt/TCF in Liver Cancer Cells to Regulate YAP and C/EBPalpha Function,” Molecular Cell 51, no. 2 (2013): 211–225. 23769673 10.1016/j.molcel.2013.05.013 PMC4007693

[393] X. Luo , R. Zhang , S. Schefczyk , et al., “Nuclear Translocation of YAP Drives BMI‐associated Hepatocarcinogenesis in hepatitis B Virus Infection,” Liver International 43, no. 9 (2023): 2002–2016. 37312627 10.1111/liv.15628

[394] D. Wu , G. Liu , Y. Liu , et al., “Zinc Finger Protein 191 Inhibits Hepatocellular Carcinoma Metastasis Through Discs Large 1‐mediated Yes‐associated Protein Inactivation,” Hepatology 64, no. 4 (2016): 1148–1162. 27358034 10.1002/hep.28708

[395] A. G. Cox , K. L. Hwang , K. K. Brown , et al., “Yap Reprograms Glutamine Metabolism to Increase Nucleotide Biosynthesis and Enable Liver Growth,” Nature Cell Biology 18, no. 8 (2016): 886–896. 27428308 10.1038/ncb3389 PMC4990146

[396] Y. Y. Park , B. H. Sohn , R. L. Johnson , et al., “Yes‐associated Protein 1 and Transcriptional Coactivator With PDZ‐binding Motif Activate the Mammalian Target of Rapamycin Complex 1 Pathway by Regulating Amino Acid Transporters in Hepatocellular Carcinoma,” Hepatology 63, no. 1 (2016): 159–172. 26389641 10.1002/hep.28223 PMC4881866

[397] K. Martin , J. Pritchett , J. Llewellyn , et al., “PAK Proteins and YAP‐1 Signalling Downstream of Integrin Beta‐1 in Myofibroblasts Promote Liver Fibrosis,” Nature Communications 7 (2016): 12502. 10.1038/ncomms12502 PMC4992158 27535340

[398] S. H. Jeong , H. B. Kim , M. C. Kim , et al., “Hippo‐mediated Suppression of IRS2/AKT Signaling Prevents Hepatic Steatosis and Liver Cancer,” Journal of Clinical Investigation 128, no. 3 (2018): 1010–1025. 29400692 10.1172/JCI95802 PMC5824861

[399] D. F. Tschaharganeh , X. Chen , P. Latzko , et al., “Yes‐associated Protein Up‐regulates Jagged‐1 and Activates the Notch Pathway in human Hepatocellular Carcinoma,” Gastroenterology 144, no. 7 (2013): 1530–1542. e1512. 23419361 10.1053/j.gastro.2013.02.009 PMC3665638

[400] N. Razumilava and G. J. Gores , “Cholangiocarcinoma,” Lancet 383, no. 9935 (2014): 2168–2179. 24581682 10.1016/S0140-6736(13)61903-0 PMC4069226

[401] P. J. Brindley , M. Bachini , S. I. Ilyas , et al., “Cholangiocarcinoma,” Nature Reviews Disease Primers 7, no. 1 (2021): 65. 10.1038/s41572-021-00300-2 PMC9246479 34504109

[402] S. I. Ilyas , S. Affo , L. Goyal , et al., “Cholangiocarcinoma—novel Biological Insights and Therapeutic Strategies,” Nature Reviews Clinical Oncology 20, no. 7 (2023): 470–486. 10.1038/s41571-023-00770-1 PMC10601496 37188899

[403] T. F. Greten , R. Schwabe , N. Bardeesy , et al., “Immunology and Immunotherapy of Cholangiocarcinoma,” Nature Reviews Gastroenterology & Hepatology 20, no. 6 (2023): 349–365. 36697706 10.1038/s41575-022-00741-4 PMC12468729

[404] R. K. Kelley , J. Bridgewater , G. J. Gores , and A. X. Zhu , “Systemic Therapies for Intrahepatic Cholangiocarcinoma,” Journal of Hepatology 72, no. 2 (2020): 353–363. 31954497 10.1016/j.jhep.2019.10.009

[405] S. Xu , Z. Yuan , C. Jiang , W. Chen , Q. Li , and T. Chen , “DNMT3A Cooperates With YAP/TAZ to Drive Gallbladder Cancer Metastasis,” Advanced Science (Weinh) 11, no. 16 (2024): e2308531. 10.1002/advs.202308531 PMC11040361 38380551

[406] J. Li , N. Razumilava , G. J. Gores , et al., “Biliary Repair and Carcinogenesis Are Mediated by IL‐33‐dependent Cholangiocyte Proliferation,” Journal of Clinical Investigation 124, no. 7 (2014): 3241–3251. 24892809 10.1172/JCI73742 PMC4071370

[407] Y. Zhang , H. Xu , G. Cui , et al., “beta‐Catenin Sustains and Is Required for YES‐associated Protein Oncogenic Activity in Cholangiocarcinoma,” Gastroenterology 163, no. 2 (2022): 481–494. 35489428 10.1053/j.gastro.2022.04.028 PMC9329198

[408] J. P. Neoptolemos , C. Springfeld , and T. Hackert , “A Review of Pancreatic Cancer,” Jama 326, no. 23 (2021): 2436. 10.1001/jama.2021.20065 34932083

[409] W. Park , A. Chawla , and E. M. O'Reilly , “Pancreatic Cancer: A Review,” Jama 326, no. 9 (2021): 851–862. 34547082 10.1001/jama.2021.13027 PMC9363152

[410] C. J. Halbrook , C. A. Lyssiotis , M. Pasca di Magliano , and A. Maitra , “Pancreatic Cancer: Advances and Challenges,” Cell 186, no. 8 (2023): 1729–1754. 37059070 10.1016/j.cell.2023.02.014 PMC10182830

[411] J. Kleeff , M. Korc , M. Apte , et al., “Pancreatic Cancer,” Nature Reviews Disease Primers 2 (2016): 16022. 10.1038/nrdp.2016.22 27158978

[412] A. P. Klein , “Pancreatic Cancer Epidemiology: Understanding the Role of Lifestyle and Inherited Risk Factors,” Nature Reviews Gastroenterology & Hepatology 18, no. 7 (2021): 493–502. 34002083 10.1038/s41575-021-00457-x PMC9265847

[413] Z. I. Hu and E. M. O'Reilly , “Therapeutic Developments in Pancreatic Cancer,” Nature Reviews Gastroenterology & Hepatology 21, no. 1 (2024): 7–24. 37798442 10.1038/s41575-023-00840-w

[414] J. P. Neoptolemos , J. Kleeff , P. Michl , E. Costello , W. Greenhalf , and D. H. Palmer , “Therapeutic Developments in Pancreatic Cancer: Current and Future Perspectives,” Nature Reviews Gastroenterology & Hepatology 15, no. 6 (2018): 333–348. 29717230 10.1038/s41575-018-0005-x

[415] M. Liu , Y. Zhang , J. Yang , et al., “Zinc‐Dependent Regulation of ZEB1 and YAP1 Coactivation Promotes Epithelial‐Mesenchymal Transition Plasticity and Metastasis in Pancreatic Cancer,” Gastroenterology 160, no. 5 (2021): 1771–1783. e1771. 33421513 10.1053/j.gastro.2020.12.077 PMC8035249

[416] A. Kapoor , W. Yao , H. Ying , et al., “Yap1 Activation Enables Bypass of Oncogenic Kras Addiction in Pancreatic Cancer,” Cell 158, no. 1 (2014): 185–197. 24954535 10.1016/j.cell.2014.06.003 PMC4109295

[417] I. Cebola , S. A. Rodriguez‐Segui , C. H. Cho , et al., “TEAD and YAP Regulate the Enhancer Network of human Embryonic Pancreatic Progenitors,” Nature Cell Biology 17, no. 5 (2015): 615–626. 25915126 10.1038/ncb3160 PMC4434585

[418] F. Li , V. Negi , P. Yang , et al., “TEAD1 regulates Cell Proliferation Through a Pocket‐independent Transcription Repression Mechanism,” Nucleic Acids Research 50, no. 22 (2022): 12723–12738. 36484096 10.1093/nar/gkac1063 PMC9825168

[419] R. Gruber , R. Panayiotou , E. Nye , B. Spencer‐Dene , G. Stamp , and A. Behrens , “YAP1 and TAZ Control Pancreatic Cancer Initiation in Mice by Direct Up‐regulation of JAK‐STAT3 Signaling,” Gastroenterology 151, no. 3 (2016): 526–539. 27215660 10.1053/j.gastro.2016.05.006 PMC5007286

[420] S. Shen , X. Guo , H. Yan , et al., “A miR‐130a‐YAP Positive Feedback Loop Promotes Organ Size and Tumorigenesis,” Cell Research 25, no. 9 (2015): 997–1012. 26272168 10.1038/cr.2015.98 PMC4559818

[421] J. Liu , W. Bai , T. Zhou , et al., “SDCBP Promotes Pancreatic Cancer Progression by Preventing YAP1 From Beta‐TrCP‐mediated Proteasomal Degradation,” Gut 72, no. 9 (2023): 1722–1737. 36828627 10.1136/gutjnl-2022-327492

[422] C. Zhao , J. Gong , Y. Bai , et al., “A Self‐amplifying USP14‐TAZ Loop Drives the Progression and Liver Metastasis of Pancreatic Ductal Adenocarcinoma,” Cell Death and Differentiation 30, no. 1 (2023): 1–15. 35906484 10.1038/s41418-022-01040-w PMC9883464

[423] X. Luo , N. A. Campbell , L. He , et al., “Sulfatase 2 (SULF2) Monoclonal Antibody 5D5 Suppresses Human Cholangiocarcinoma Xenograft Growth through Regulation of a SULF2‐Platelet‐Derived Growth Factor Receptor Beta‐Yes‐Associated Protein Signaling Axis,” Hepatology 74, no. 3 (2021): 1411–1428. 33735525 10.1002/hep.31817 PMC9075007

[424] N. Li , G. Yang , L. Luo , et al., “lncRNA THAP9‐AS1 Promotes Pancreatic Ductal Adenocarcinoma Growth and Leads to a Poor Clinical Outcome via Sponging miR‐484 and Interacting With YAP,” Clinical Cancer Research 26, no. 7 (2020): 1736–1748. 31831555 10.1158/1078-0432.CCR-19-0674

[425] E. Cortes , M. Sarper , B. Robinson , et al., “GPER Is a Mechanoregulator of Pancreatic Stellate Cells and the Tumor Microenvironment,” Embo Reports 20, no. 1 (2019): e46556. 30538117 10.15252/embr.201846556 PMC6322386

[426] S. S. Mello , L. J. Valente , N. Raj , et al., “A p53 Super‐tumor Suppressor Reveals a Tumor Suppressive p53‐Ptpn14‐Yap Axis in Pancreatic Cancer,” Cancer Cell 32, no. 4 (2017): 460–473. e466. 29017057 10.1016/j.ccell.2017.09.007 PMC5659188

[427] Y. Aylon and M. Oren , “Tumor Suppression by p53: Bring in the Hippo!,” Cancer Cell 32, no. 4 (2017): 397–399. 29017051 10.1016/j.ccell.2017.09.010

[428] E. Dekker , P. J. Tanis , J. L. A. Vleugels , P. M. Kasi , and M. B. Wallace , “Colorectal Cancer,” Lancet 394, no. 10207 (2019): 1467–1480. 31631858 10.1016/S0140-6736(19)32319-0

[429] L. H. Biller and D. Schrag , “Diagnosis and Treatment of Metastatic Colorectal Cancer: A Review,” Jama 325, no. 7 (2021): 669–685. 33591350 10.1001/jama.2021.0106

[430] M. C. W. Spaander , A. G. Zauber , S. Syngal , et al., “Young‐onset Colorectal Cancer,” Nature Reviews Disease Primers 9, no. 1 (2023): 21. 10.1038/s41572-023-00432-7 PMC10589420 37105987

[431] E. J. Kuipers , W. M. Grady , D. Lieberman , et al., “Colorectal Cancer,” Nature Reviews Disease Primers 1 (2015): 15065. 10.1038/nrdp.2015.65 PMC4874655 27189416

[432] N. Keum and E. Giovannucci , “Global Burden of Colorectal Cancer: Emerging Trends, Risk Factors and Prevention Strategies,” Nature Reviews Gastroenterology & Hepatology 16, no. 12 (2019): 713–732. 31455888 10.1038/s41575-019-0189-8

[433] C. Eng , T. Yoshino , E. Ruiz‐Garcia , et al., “Colorectal Cancer,” Lancet 404, no. 10449 (2024): 294–310. 38909621 10.1016/S0140-6736(24)00360-X

[434] J. Weitz , M. Koch , J. Debus , T. Hohler , P. R. Galle , and M. W. Buchler , “Colorectal Cancer,” Lancet 365, no. 9454 (2005): 153–165. 15639298 10.1016/S0140-6736(05)17706-X

[435] K. W. Lee , S. S. Lee , S. B. Kim , et al., “Significant Association of Oncogene YAP1 With Poor Prognosis and Cetuximab Resistance in Colorectal Cancer Patients,” Clinical Cancer Research 21, no. 2 (2015): 357–364. 25388162 10.1158/1078-0432.CCR-14-1374 PMC4513664

[436] E. R. Barry , T. Morikawa , B. L. Butler , et al., “Restriction of Intestinal Stem Cell Expansion and the Regenerative Response by YAP,” Nature 493, no. 7430 (2013): 106–110. 23178811 10.1038/nature11693 PMC3536889

[437] G. Della Chiara , F. Gervasoni , M. Fakiola , et al., “Epigenomic Landscape of human Colorectal Cancer Unveils an Aberrant Core of Pan‐cancer Enhancers Orchestrated by YAP/TAZ,” Nature Communications 12, no. 1 (2021): 2340. 10.1038/s41467-021-22544-y PMC8058065 33879786

[438] L. Fang , H. Teng , Y. Wang , et al., “SET1A‐Mediated Mono‐Methylation at K342 Regulates YAP Activation by Blocking Its Nuclear Export and Promotes Tumorigenesis,” Cancer Cell 34, no. 1 (2018): 103–118. e109. 30008322 10.1016/j.ccell.2018.06.002

[439] Q. Wang , X. Gao , T. Yu , et al., “REGgamma Controls Hippo Signaling and Reciprocal NF‐kappaB‐YAP Regulation to Promote Colon Cancer,” Clinical Cancer Research 24, no. 8 (2018): 2015–2025. 29437787 10.1158/1078-0432.CCR-17-2986

[440] J. Xu , Y. Tang , X. Sheng , et al., “Secreted Stromal Protein ISLR Promotes Intestinal Regeneration by Suppressing Epithelial Hippo Signaling,” Embo Journal 39, no. 7 (2020): e103255. 32128839 10.15252/embj.2019103255 PMC7110107

[441] Y. Pan , J. H. M. Tong , R. W. M. Lung , et al., “RASAL2 promotes Tumor Progression Through LATS2/YAP1 Axis of Hippo Signaling Pathway in Colorectal Cancer,” Molecular Cancer 17, no. 1 (2018): 102. 30037330 10.1186/s12943-018-0853-6 PMC6057036

[442] K. Zeng , J. Peng , Y. Xing , et al., “A Positive Feedback Circuit Driven by M(6)A‐modified Circular RNA Facilitates Colorectal Cancer Liver Metastasis,” Molecular Cancer 22, no. 1 (2023): 202. 38087322 10.1186/s12943-023-01848-1 PMC10717141

[443] H. B. Kim , M. Kim , Y. S. Park , et al., “Prostaglandin E(2) Activates YAP and a Positive‐Signaling Loop to Promote Colon Regeneration after Colitis but Also Carcinogenesis in Mice,” Gastroenterology 152, no. 3 (2017): 616–630. 27864128 10.1053/j.gastro.2016.11.005 PMC5285392

[444] Y. Touil , W. Igoudjil , M. Corvaisier , et al., “Colon Cancer Cells Escape 5FU Chemotherapy‐induced Cell Death by Entering Stemness and Quiescence Associated With the c‐Yes/YAP Axis,” Clinical Cancer Research 20, no. 4 (2014): 837–846. 24323901 10.1158/1078-0432.CCR-13-1854 PMC4387277

[445] N. Ferrari , R. Ranftl , I. Chicherova , et al., “Dickkopf‐3 Links HSF1 and YAP/TAZ Signalling to Control Aggressive Behaviours in Cancer‐associated Fibroblasts,” Nature Communications 10, no. 1 (2019): 130. 10.1038/s41467-018-07987-0 PMC6328607 30631061

[446] Y. Gu , Y. Chen , L. Wei , et al., “ABHD5 inhibits YAP‐induced c‐Met Overexpression and Colon Cancer Cell Stemness via Suppressing YAP Methylation,” Nature Communications 12, no. 1 (2021): 6711. 10.1038/s41467-021-26967-5 PMC8602706 34795238

[447] Y. Guo , Z. Zhu , Z. Huang , et al., “CK2‐induced Cooperation of HHEX With the YAP‐TEAD4 Complex Promotes Colorectal Tumorigenesis,” Nature Communications 13, no. 1 (2022): 4995. 10.1038/s41467-022-32674-6 PMC9411202 36008411

[448] Q. Pan , S. Zhong , H. Wang , et al., “The ZMYND8‐regulated Mevalonate Pathway Endows YAP‐high Intestinal Cancer With Metabolic Vulnerability,” Molecular Cell 81, no. 13 (2021): 2736–2751. e2738. 33932349 10.1016/j.molcel.2021.04.009

[449] S. Jiao , C. Li , Q. Hao , et al., “VGLL4 targets a TCF4‐TEAD4 Complex to Coregulate Wnt and Hippo Signalling in Colorectal Cancer,” Nature Communications 8 (2017): 14058. 10.1038/ncomms14058 PMC5216127 28051067

[450] H. Y. Wang , Q. Y. Long , S. B. Tang , et al., “Histone Demethylase KDM3A Is Required for Enhancer Activation of Hippo Target Genes in Colorectal Cancer,” Nucleic Acids Research 47, no. 5 (2019): 2349–2364. 30649550 10.1093/nar/gky1317 PMC6412006

[451] H. Xu , S. Zhou , H. Xia , H. Yu , Q. Tang , and F. Bi , “MEK Nuclear Localization Promotes YAP Stability via Sequestering Beta‐TrCP in KRAS Mutant Cancer Cells,” Cell Death and Differentiation 26, no. 11 (2019): 2400–2415. 30833665 10.1038/s41418-019-0309-6 PMC6889282

[452] B. Pan , Y. Yang , J. Li , et al., “USP47‐mediated Deubiquitination and Stabilization of YAP Contributes to the Progression of Colorectal Cancer,” Protein Cell 11, no. 2 (2020): 138–143. 31748975 10.1007/s13238-019-00674-w PMC6954888

[453] M. Sun , H. Song , S. Wang , et al., “Integrated Analysis Identifies microRNA‐195 as a Suppressor of Hippo‐YAP Pathway in Colorectal Cancer,” Journal of Hematology & Oncology 10, no. 1 (2017): 79. 28356122 10.1186/s13045-017-0445-8 PMC5372308

[454] Z. Diamantopoulou , G. White , M. Z. H. Fadlullah , et al., “TIAM1 Antagonizes TAZ/YAP both in the Destruction Complex in the Cytoplasm and in the Nucleus to Inhibit Invasion of Intestinal Epithelial Cells,” Cancer Cell 31, no. 5 (2017): 621–634. e626. 28416184 10.1016/j.ccell.2017.03.007 PMC5425402

[455] L. E. L. Hendriks , J. Remon , C. Faivre‐Finn , et al., “Non‐small‐cell Lung Cancer,” Nature Reviews Disease Primers 10, no. 1 (2024): 71. 10.1038/s41572-024-00551-9 39327441

[456] C. Gridelli , A. Rossi , D. P. Carbone , et al., “Non‐small‐cell Lung Cancer,” Nature Reviews Disease Primers 1 (2015): 15009. 10.1038/nrdp.2015.9 27188576

[457] A. A. Thai , B. J. Solomon , L. V. Sequist , J. F. Gainor , and R. S. Heist , “Lung Cancer,” Lancet 398, no. 10299 (2021): 535–554. 34273294 10.1016/S0140-6736(21)00312-3

[458] R. Barnett , “Lung Cancer,” Lancet 390, no. 10098 (2017): 928. 28872022 10.1016/S0140-6736(17)32243-2

[459] S. C. M. Lau , Y. Pan , V. Velcheti , and K. K. Wong , “Squamous Cell Lung Cancer: Current Landscape and Future Therapeutic Options,” Cancer Cell 40, no. 11 (2022): 1279–1293. 36270277 10.1016/j.ccell.2022.09.018

[460] F. R. Hirsch , G. V. Scagliotti , J. L. Mulshine , et al., “Lung Cancer: Current Therapies and New Targeted Treatments,” Lancet 389, no. 10066 (2017): 299–311. 27574741 10.1016/S0140-6736(16)30958-8

[461] R. S. Herbst , D. Morgensztern , and C. Boshoff , “The Biology and Management of Non‐small Cell Lung Cancer,” Nature 553, no. 7689 (2018): 446–454. 29364287 10.1038/nature25183

[462] A. Lahiri , A. Maji , P. D. Potdar , et al., “Lung Cancer Immunotherapy: Progress, Pitfalls, and Promises,” Molecular Cancer 22, no. 1 (2023): 40. 36810079 10.1186/s12943-023-01740-y PMC9942077

[463] S. M. Pearsall , S. Humphrey , M. Revill , et al., “The Rare YAP1 Subtype of SCLC Revisited in a Biobank of 39 Circulating Tumor Cell Patient Derived Explant Models: A Brief Report,” Journal of Thoracic Oncology 15, no. 12 (2020): 1836–1843. 32721553 10.1016/j.jtho.2020.07.008 PMC7718082

[464] Y. T. Shue , A. P. Drainas , N. Y. Li , et al., “A Conserved YAP/Notch/REST Network Controls the Neuroendocrine Cell Fate in the Lungs,” Nature Communications 13, no. 1 (2022): 2690. 10.1038/s41467-022-30416-2 PMC9110333 35577801

[465] B. Zhou , P. Flodby , J. Luo , et al., “Claudin‐18‐mediated YAP Activity Regulates Lung Stem and Progenitor Cell Homeostasis and Tumorigenesis,” Journal of Clinical Investigation 128, no. 3 (2018): 970–984. 29400695 10.1172/JCI90429 PMC5824875

[466] D. N. Kotton , “Claudin‐18: Unexpected Regulator of Lung Alveolar Epithelial Cell Proliferation,” Journal of Clinical Investigation 128, no. 3 (2018): 903–905. 29400691 10.1172/JCI99799 PMC5824920

[467] X. Rong , Y. Liang , Q. Han , et al., “Molecular Mechanisms of Tyrosine Kinase Inhibitor Resistance Induced by Membranous/Cytoplasmic/Nuclear Translocation of Epidermal Growth Factor Receptor,” Journal of Thoracic Oncology 14, no. 10 (2019): 1766–1783. 31228625 10.1016/j.jtho.2019.06.014

[468] J. Jin , L. Zhang , X. Li , et al., “Oxidative Stress‐CBP Axis Modulates MOB1 Acetylation and Activates the Hippo Signaling Pathway,” Nucleic Acids Research 50, no. 7 (2022): 3817–3834. 35349706 10.1093/nar/gkac189 PMC9023286

[469] C. Xu , G. Jin , H. Wu , et al., “SIRPgamma‐expressing Cancer Stem‐Like Cells Promote Immune Escape of Lung Cancer via Hippo Signaling,” Journal of Clinical Investigation 132, no. 5 (2022): e141797. 35229723 10.1172/JCI141797 PMC8884909

[470] D. Jin , J. Guo , Y. Wu , et al., “m(6)A Demethylase ALKBH5 Inhibits Tumor Growth and Metastasis by Reducing YTHDFs‐mediated YAP Expression and Inhibiting miR‐107/LATS2‐mediated YAP Activity in NSCLC,” Molecular Cancer 19, no. 1 (2020): 40. 32106857 10.1186/s12943-020-01161-1 PMC7045432

[471] I. Chaib , N. Karachaliou , S. Pilotto , et al., “Co‐activation of STAT3 and YES‐Associated Protein 1 (YAP1) Pathway in EGFR‐Mutant NSCLC,” JNCI: Journal of the National Cancer Institute 109, no. 9 (2017): djx014. 28376152 10.1093/jnci/djx014 PMC5409000

[472] K. J. Kurppa , Y. Liu , C. To , et al., “Treatment‐Induced Tumor Dormancy Through YAP‐Mediated Transcriptional Reprogramming of the Apoptotic Pathway,” Cancer Cell 37, no. 1 (2020): 104–122. e112. 31935369 10.1016/j.ccell.2019.12.006 PMC7146079

[473] Y. Gao , W. Zhang , X. Han , et al., “YAP Inhibits Squamous Transdifferentiation of Lkb1‐Deficient Lung Adenocarcinoma Through ZEB2‐dependent DNp63 Repression,” Nature Communications 5 (2014): 4629. 10.1038/ncomms5629 25115923

[474] T. Tsuji , H. Ozasa , W. Aoki , et al., “YAP1 mediates Survival of ALK‐rearranged Lung Cancer Cells Treated With alectinib via Pro‐apoptotic Protein Regulation,” Nature Communications 11, no. 1 (2020): 74. 10.1038/s41467-019-13771-5 PMC6941996 31900393

[475] U. Capitanio and F. Montorsi , “Renal Cancer,” Lancet 387, no. 10021 (2016): 894–906. 26318520 10.1016/S0140-6736(15)00046-X

[476] J. J. Hsieh , M. P. Purdue , S. Signoretti , et al., “Renal Cell Carcinoma,” Nature Reviews Disease Primers 3 (2017): 17009. 10.1038/nrdp.2017.9 PMC5936048 28276433

[477] P. C. Barata and B. I. Rini , “Treatment of Renal Cell Carcinoma: Current Status and Future Directions,” CA: A Cancer Journal for Clinicians 67, no. 6 (2017): 507–524. 28961310 10.3322/caac.21411

[478] S. Chevrier , J. H. Levine , V. R. T. Zanotelli , et al., “An Immune Atlas of Clear Cell Renal Cell Carcinoma,” Cell 169, no. 4 (2017): 736–749. e718. 28475899 10.1016/j.cell.2017.04.016 PMC5422211

[479] W. K. Rathmell , R. B. Rumble , P. J. Van Veldhuizen , et al., “Management of Metastatic Clear Cell Renal Cell Carcinoma: ASCO Guideline,” Journal of Clinical Oncology 40, no. 25 (2022): 2957–2995. 35728020 10.1200/JCO.22.00868

[480] L. Qu , Z. Wu , Y. Li , et al., “A Feed‐forward Loop Between lncARSR and YAP Activity Promotes Expansion of Renal Tumour‐initiating Cells,” Nature Communications 7 (2016): 12692. 10.1038/ncomms12692 PMC5133634 27886176

[481] C. Wang , Y. Wang , T. Hong , et al., “Targeting a Positive Regulatory Loop in the Tumor‐macrophage Interaction Impairs the Progression of Clear Cell Renal Cell Carcinoma,” Cell Death and Differentiation 28, no. 3 (2021): 932–951. 33009518 10.1038/s41418-020-00626-6 PMC7937678

[482] T. He , Q. Zhang , P. Xu , et al., “Extracellular Vesicle‐circEHD2 Promotes the Progression of Renal Cell Carcinoma by Activating Cancer‐associated Fibroblasts,” Molecular Cancer 22, no. 1 (2023): 117. 37481520 10.1186/s12943-023-01824-9 PMC10362694

[483] P. Carter , U. Schnell , C. Chaney , et al., “Deletion of Lats1/2 in Adult Kidney Epithelia Leads to Renal Cell Carcinoma,” Journal of Clinical Investigation 131, no. 11 (2021): e144108. 34060480 10.1172/JCI144108 PMC8159698

[484] A. T. Lenis , P. M. Lec , K. Chamie , and M. D. Mshs , “Bladder Cancer: A Review,” Jama 324, no. 19 (2020): 1980–1991. 33201207 10.1001/jama.2020.17598

[485] L. Dyrskjot , D. E. Hansel , J. A. Efstathiou , et al., “Bladder Cancer,” Nature Reviews Disease Primers 9, no. 1 (2023): 58. 10.1038/s41572-023-00468-9 PMC11218610 37884563

[486] O. Sanli , J. Dobruch , M. A. Knowles , et al., “Bladder Cancer,” Nature Reviews Disease Primers 3 (2017): 17022. 10.1038/nrdp.2017.22 28406148

[487] E. Comperat , M. B. Amin , R. Cathomas , et al., “Current Best Practice for Bladder Cancer: A Narrative Review of Diagnostics and Treatments,” Lancet 400, no. 10364 (2022): 1712–1721. 36174585 10.1016/S0140-6736(22)01188-6

[488] M. A. Knowles and C. D. Hurst , “Molecular Biology of Bladder Cancer: New Insights Into Pathogenesis and Clinical Diversity,” Nature Reviews Cancer 15, no. 1 (2015): 25–41. 25533674 10.1038/nrc3817

[489] C. P. Dinney , D. J. McConkey , R. E. Millikan , et al., “Focus on Bladder Cancer,” Cancer Cell 6, no. 2 (2004): 111–116. 15324694 10.1016/j.ccr.2004.08.002

[490] D. J. Parekh , B. H. Bochner , and G. Dalbagni , “Superficial and Muscle‐invasive Bladder Cancer: Principles of Management for Outcomes Assessments,” Journal of Clinical Oncology 24, no. 35 (2006): 5519–5527. 17158537 10.1200/JCO.2006.08.5431

[491] F. M. Torti and B. L. Lum , “The Biology and Treatment of Superficial Bladder Cancer,” Journal of Clinical Oncology 2, no. 5 (1984): 505–531. 6427417 10.1200/JCO.1984.2.5.505

[492] B. Xie , J. Lin , X. Chen , et al., “CircXRN2 suppresses Tumor Progression Driven by Histone Lactylation Through Activating the Hippo Pathway in human Bladder Cancer,” Molecular Cancer 22, no. 1 (2023): 151. 37684641 10.1186/s12943-023-01856-1 PMC10486081

[493] M. K. Gill , T. Christova , Y. Y. Zhang , et al., “A Feed Forward Loop Enforces YAP/TAZ Signaling During Tumorigenesis,” Nature Communications 9, no. 1 (2018): 3510. 10.1038/s41467-018-05939-2 PMC6115388 30158528

[494] R. J. Rebello , C. Oing , K. E. Knudsen , et al., “Prostate Cancer,” Nature Reviews Disease Primers 7, no. 1 (2021): 9. 10.1038/s41572-020-00243-0 33542230

[495] J. F. Rodriguez‑Moreno and J. Garcia‑Donas , “Metastatic Prostate Cancer,” New England Journal of Medicine 378, no. 17 (2018): 1653. 10.1056/NEJMc1803343 29697920

[496] O. Sartor and J. S. Bono , “Metastatic Prostate Cancer,” New England Journal of Medicine 378, no. 17 (2018): 1653–1654. 10.1056/NEJMc1803343 29694820

[497] H. I. Scher , “Defining New Standards of Care for Men With Prostate Cancer,” Lancet 387, no. 10024 (2016): 1135–1137. 27025318 10.1016/S0140-6736(15)01235-0

[498] G. Attard , C. Parker , R. A. Eeles , et al., “Prostate Cancer,” Lancet 387, no. 10013 (2016): 70–82. 26074382 10.1016/S0140-6736(14)61947-4

[499] L. T. Nguyen , M. S. Tretiakova , M. R. Silvis , et al., “ERG Activates the YAP1 Transcriptional Program and Induces the Development of Age‐Related Prostate Tumors,” Cancer Cell 27, no. 6 (2015): 797–808. 26058078 10.1016/j.ccell.2015.05.005 PMC4461839

[500] T. D. Kim , F. Jin , S. Shin , et al., “Histone Demethylase JMJD2A Drives Prostate Tumorigenesis Through Transcription Factor ETV1,” Journal of Clinical Investigation 126, no. 2 (2016): 706–720. 26731476 10.1172/JCI78132 PMC4731184

[501] G. Wang , X. Lu , P. Dey , et al., “Targeting YAP‐Dependent MDSC Infiltration Impairs Tumor Progression,” Cancer Discovery 6, no. 1 (2016): 80–95. 26701088 10.1158/2159-8290.CD-15-0224 PMC4707102

[502] J. Xie , Y. Fan , R. Jia , F. Yang , L. Ma , and L. Li , “Yes‐associated Protein Regulates the Hepatoprotective Effect of Vitamin D Receptor Activation Through Promoting Adaptive Bile Duct Remodeling in Cholestatic Mice,” Journal of Pathology 255, no. 1 (2021): 95–106. 34156701 10.1002/path.5750

[503] H. Kagawa , A. Javali , H. H. Khoei , et al., “Human Blastoids Model Blastocyst Development and Implantation,” Nature 601, no. 7894 (2022): 600–605. 34856602 10.1038/s41586-021-04267-8 PMC8791832

[504] J. H. Won , J. S. Choi , and J. I. Jun , “CCN1 interacts With Integrins to Regulate Intestinal Stem Cell Proliferation and Differentiation,” Nature Communications 13, no. 1 (2022): 3117. 10.1038/s41467-022-30851-1 PMC9166801 35660741

[505] S. Ma , Z. Meng , R. Chen , and K. L. Guan , “The Hippo Pathway: Biology and Pathophysiology,” Annual Review of Biochemistry 88 (2019): 577–604. 10.1146/annurev-biochem-013118-111829 30566373

[506] A. W. Hong , Z. Meng , and K. L. Guan , “The Hippo Pathway in Intestinal Regeneration and Disease,” Nature Reviews Gastroenterology & Hepatology 13, no. 6 (2016): 324–337. 27147489 10.1038/nrgastro.2016.59 PMC5642988

[507] Q. Liu , J. Li , W. Zhang , et al., “Glycogen Accumulation and Phase Separation Drives Liver Tumor Initiation,” Cell 184, no. 22 (2021): 5559–5576. e5519. 34678143 10.1016/j.cell.2021.10.001

[508] F. X. Yu , B. Zhao , and K. L. Guan , “Hippo Pathway in Organ Size Control, Tissue Homeostasis, and Cancer,” Cell 163, no. 4 (2015): 811–828. 26544935 10.1016/j.cell.2015.10.044 PMC4638384

[509] Q. Zeng and W. Hong , “The Emerging Role of the Hippo Pathway in Cell Contact Inhibition, Organ Size Control, and Cancer Development in Mammals,” Cancer Cell 13, no. 3 (2008): 188–192. 18328423 10.1016/j.ccr.2008.02.011

[510] P. Yadav , B. Bhatt , and K. N. Balaji , “Selective Activation of MST1/2 Kinases by Retinoid Agonist Adapalene Abrogates AURKA‐Regulated Septic Arthritis,” Journal of Immunology 206, no. 12 (2021): 2888–2899. 10.4049/jimmunol.2001360 34031150

[511] T. Moroishi , T. Hayashi , W. W. Pan , et al., “The Hippo Pathway Kinases LATS1/2 Suppress Cancer Immunity,” Cell 167, no. 6 (2016): 1525–1539. e1517. 27912060 10.1016/j.cell.2016.11.005 PMC5512418

[512] A. Britschgi , S. Duss , S. Kim , et al., “The Hippo Kinases LATS1 and 2 Control human Breast Cell Fate via Crosstalk With ERalpha,” Nature 541, no. 7638 (2017): 541–545. 28068668 10.1038/nature20829 PMC6726477

[513] M. Park , J. Jin , D. Y. An , et al., “Targeting YAP Activity and Glutamine Metabolism Cooperatively Suppresses Tumor Progression by Preventing Extracellular Matrix Accumulation,” Cancer Research 84, no. 20 (2024): 3388–3401. 39073839 10.1158/0008-5472.CAN-23-3933

