# Supplementary material for: The Role of Yes‐Associated Protein in Inflammatory Diseases and Cancer
Source: MedComm (2020). 2025 Mar 10;6(3):e70128. doi: 10.1002/mco2.70128 (PMC11892025; doi:10.1002/mco2.70128)
Supplement: Supplementary file 1 — Supporting Information [file MCO2-6-e70128-s001.docx]

The role of Yes-associated protein in inflammatory diseases and cancer

Bing Zhong^1^, Jintao Du^1*^, Feng Liu^1*^, Silu Sun^2*^

^1^ Department of Otolaryngology-Head and Neck Surgery, West China Hospital, Sichuan University, Chengdu, Sichuan, China

^2^ State Key Laboratory of Oral Diseases, National Clinical Research Center for Oral Diseases, Chinese Academy of Medical Sciences Research Unit of Oral Carcinogenesis and Management, West China Hospital of Stomatology, Sichuan University, Chengdu, Sichuan, China

**This file includes:**

**Figures. S1 to S4**


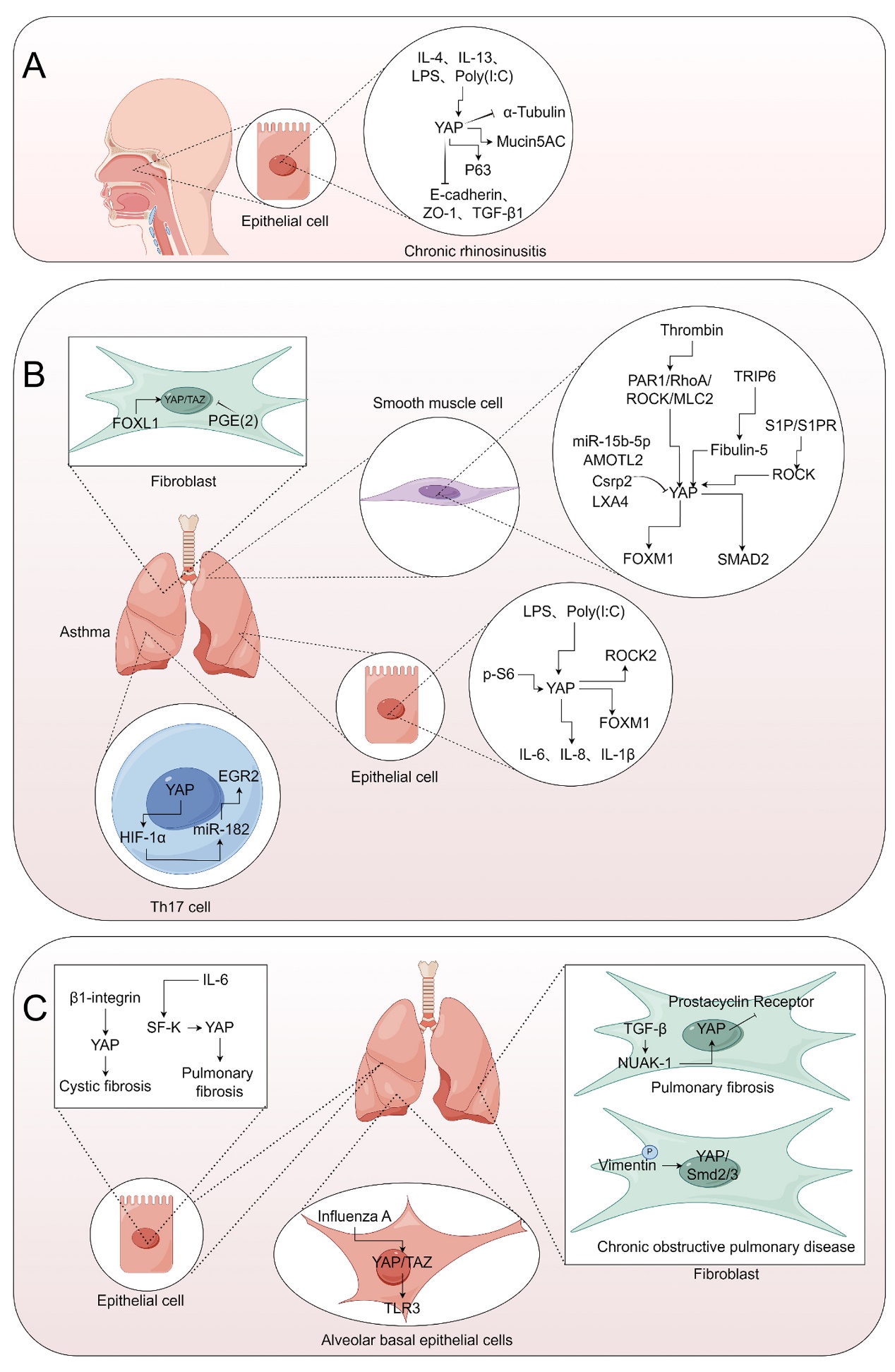


**Figure S1.** **Molecular mechanism of YAP in respiratory tract.** A. Mechanism of YAP in epithelial cells of chronic rhinosinusitis. B. Mechanism of YAP in Epithelial cells, Fibroblasts, Smooth muscle cells and Th17 cells of asthma. C. Mechanism of YAP in Epithelial cells and Fibroblasts of Cystic fibrosis and Pulmonary fibrosis; YAP mechanism of Fibroblasts in Chronic obstructive pulmonary disease; Mechanism of YAP expression of Influenza A after infection with Human alveolar basal epithelial cells. IL: Interleukin; LPS: Lipopolysaccharide; YAP: Yes-associated protein; ZO-1: Zonula occludens-1; TAZ: Transcriptional coactivator with PDZ-binding motif; TGF-β1: Transforming Growth Factor-β1; FOLX1: Folate hydrolase 1; PGE(2): Prostaglandin E2; EGR2: Early Growth Response Protein 2; HIF-1α: Hypoxia-inducible factor 1α; PAR1: Protease Activated Receptor 1 Protein; Ras homolog gene family, member A; ROCK: Rho Associated Coiled Coil Containing Protein Kinase 1; MLC2: Myosin Light Chain 2 Protein; AMOTL2: Angiomotin-like protein 2; CSRP2: Cysteine and glycine-rich protein 2; LXA4: Lipoxin A4; TRIP6: Thyroid Hormone Receptor Interactor 6; S1P: Sphingosine-1-Phosphate; ROCK: Rho-associated coiled-coil forming protein kinase; FOXM1: Forkhead box protein M1; SMAD2: SMAD family member 2; SFK: Src family kinases; TLR3: Toll-like receptor 3; NUAK-1: AMPK-related protein kinase 1; Smd2/3: Small Mothers Against Decapentaplegic 2. The Figure is created by Figdraw.


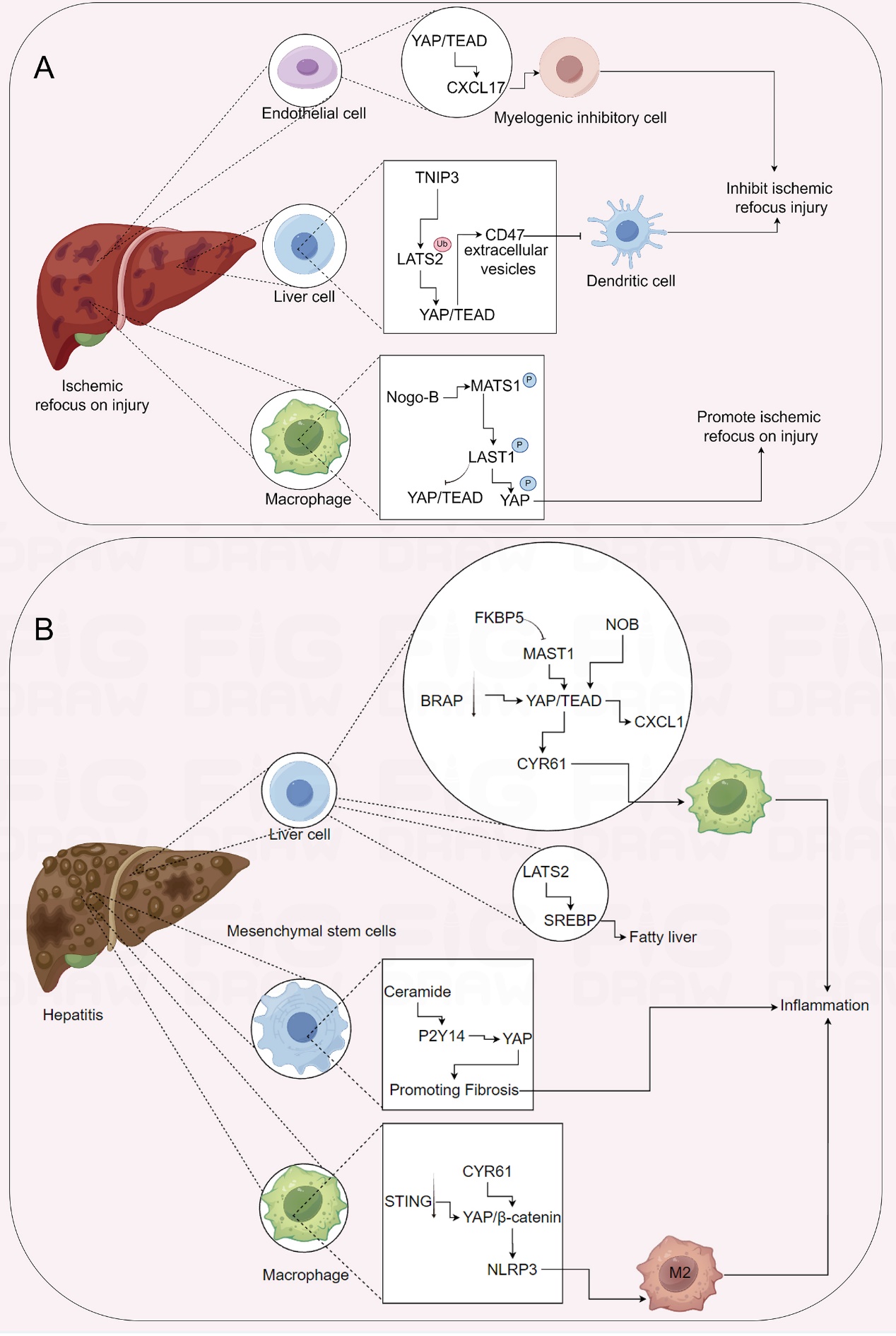


**Figure S2.** **Molecular mechanism of YAP in liver disease. A.** Mechanism of YAP in Epithelial cells, Liver cells and Macrophage of Liver ischemia-reperfusion injury. B. Mechanism of YAP in Liver cells, Mesenchymal stem cells and Macrophage of Hepatitis. YAP: Yes-associated protein; TEADs: TEA domain transcription factors; LATS2: Large tumor suppressor homolog 2; CXCL17: Chemokine (C-X-C motif) ligand 17; TNIP3: Tumor necrosis factor alpha-induced protein 3 interacting protein 3; MST1: Mammalian sterile 20-like protein kinase 1; Nogo-B: Reticulon-4B; FKBP5: FK506 binding protein 5; NOB: NIN1/RPN12 binding protein 1 homolog; BRAP: BRCA1-associated protein; CYR61: Cysteine-rich protein 61; SREBP: Sterol Regulatory Element Binding Protein; P2Y14: Purinergic Receptor P2Y, G Protein Coupled 14; NLRP3: Nucleotide-binding oligomerization domain-like receptor protein 3. The Figure is created by Figdraw.


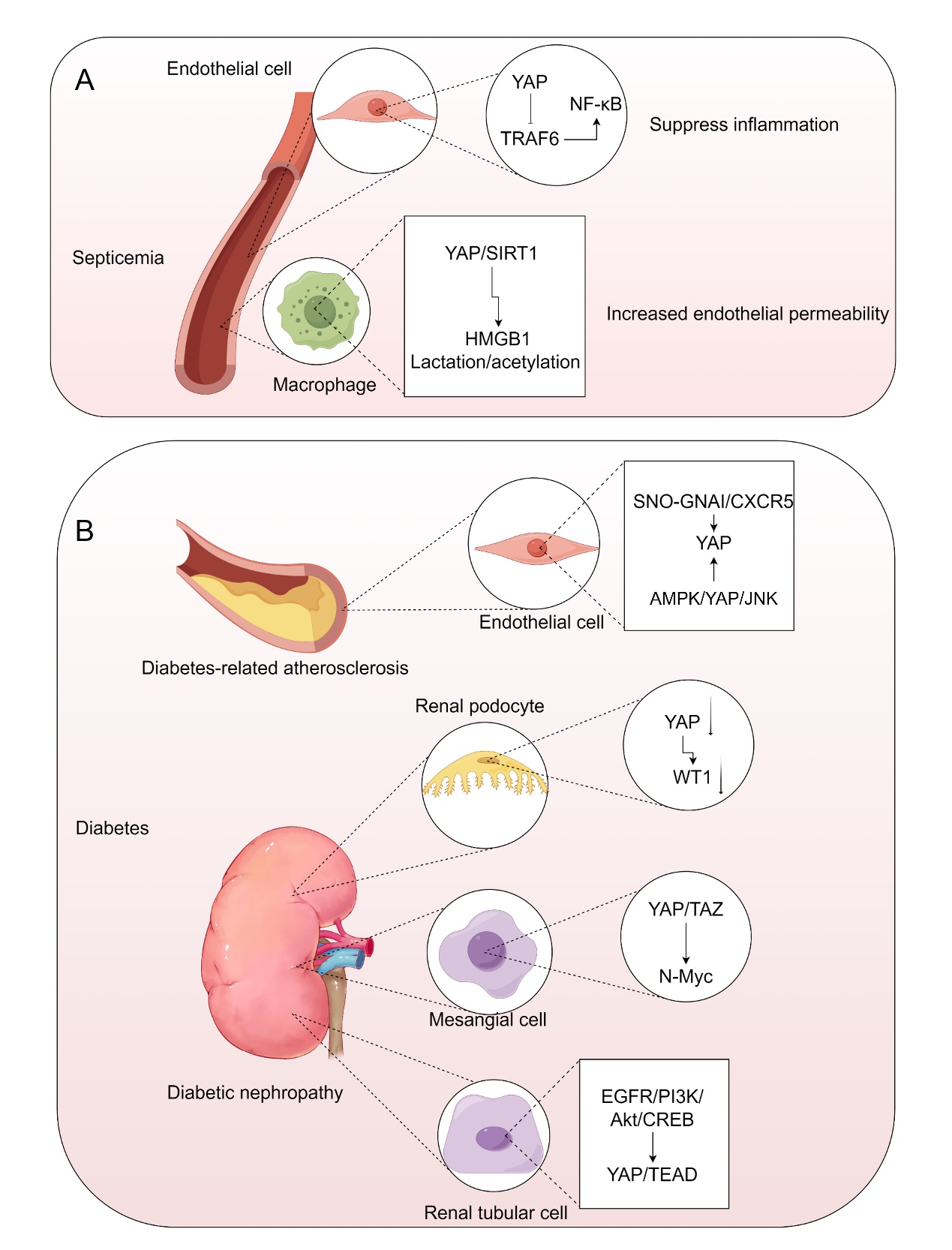
 **Figure S3.** **Molecular mechanism of YAP in Sepsis and Diabetes.** A. Mechanism of YAP in Endothelial cells and Macrophage of Sepsis. B. Mechanism of YAP in Endothelial cells of Diabetes-associated Atherosclerosis. C. Mechanism of YAP in Renal podocyte, Mesangial cells and Renal tubular cells of Diabetic nephropathy. YAP: Yes-associated protein; TAZ: Transcriptional coactivator with PDZ-binding motif; TRAF6: Tumor Necrosis Factor Receptor-Associated Factor 6; NF-κB: Nuclear Factor κB; SIRT1: Silent Information Regulator 2-Related Enzyme 1; HMGB1: High Mobility Group Box 1; SNO-GNAI: S-nitrosylated Guanine nucleotide-binding protein G (i) subunit alpha; CXCR5: Chemokine C-X-C-Motif Receptor 5; AMPK: 5' adenosine monophosphate-activated protein kinase; JNK: c-Jun N-terminal kinase; WT1: Wilms tumor 1 protein; N-myc: Neuroblastoma oncogene; EGFR: Epidermal Growth Factor Receptor; PI3K: Phosphatidylinositol 3-Kinase; Akt: Protein Kinase B; CREB: Cyclic Adenosine Monophosphate Response Element Binding Protein; TEADs: TEA domain transcription factors. The Figure is created by Figdraw.


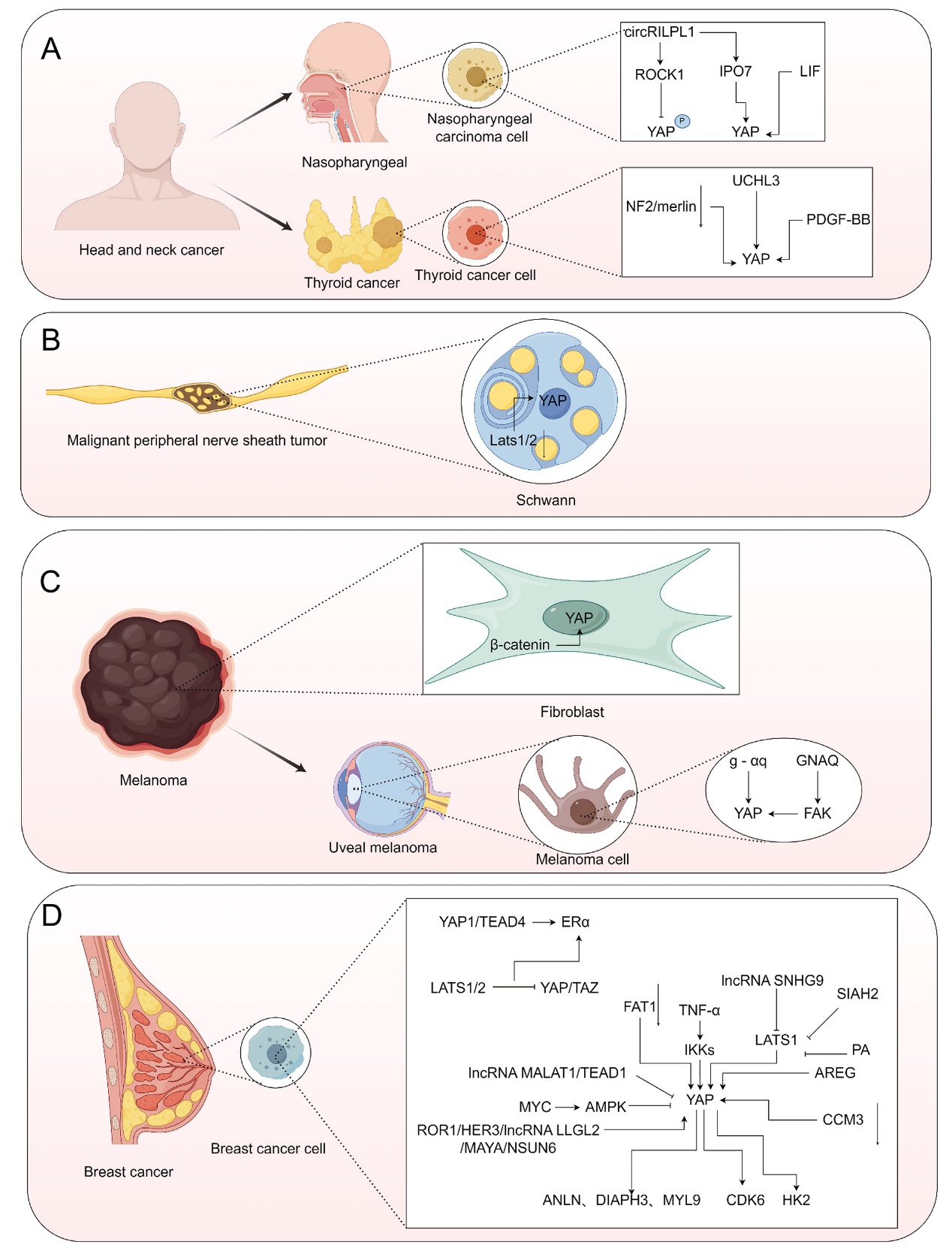


**Figure S4.** **Molecular mechanism of YAP in Head and neck cancer，Malignant peripheral nerve sheath tumors，Melanoma and Breast cancer.** A. Mechanism of YAP in Nasopharyngeal carcinoma and Thyroid cancer. B. Mechanism of YAP in Malignant peripheral nerve sheath tumors. C. Mechanism of YAP in Melanoma. D. Mechanism of YAP in Breast cancer. ROCK1: Rho-associated, coiled-coil-containing protein kinase 1; IPO7: Importin 7; LIF: Leukemia Inhibitory Factor; YAP: Yes-associated protein; NF2: Neurofibromatosis type 2; UCHL3: Ubiquitin Carboxyl Terminal Hydrolase L3; PDGF-BB: Platelet-Derived Growth Factor-BB; LATS1/2: Large tumor suppressor homolog 1/2; G-αq: G protein alpha q subunit; GNAQ: Guanine nucleotide binding protein alpha q; FAK: Focal Adhesion Kinase; ERα: Estrogen Receptor Alpha; SIAH2：Seven In Absentia Homolog 2; FAT1: FAT tumor suppressor 1; TNF-α: Tumor Necrosis Factor-alpha; IKKs: Inhibitor of Nuclear Factor κB Kinase; PA: Prealbumin; AREG: Amphiregulin; MYC: MYC Proto-Oncogene Protein; AMPK: 5' AMP-Activated Protein Kinase; CCM3: Cerebral Cavernous Malformation 3; ROR1: Receptor Tyrosine Kinase-Like Orphan Receptor 1; HER3: Human Epidermal Growth Factor Receptor 3; MAYA: Mdm2 and p53 associated protein; NSUN6: NOP2/Sun RNA Methyltransferase 6; ANLN: Anillin; DIAPH3: Diaphanous Related Formin 3; MYL9: Myosin Light Chain 9; CDK6: Cyclin-Dependent Kinase 6; HK2: Hexokinase 2. The Figure is created by Figdraw.
